# Supplementary material for: Predicting Ovarian/Breast Cancer Pathogenic Risks of Human BRCA1 Gene Variants of Unknown Significance
Source: Biomed Res Int. 2021 Apr 14;2021:6667201. doi: 10.1155/2021/6667201 (PMC8062186; doi:10.1155/2021/6667201)
Supplement: Supplementary Materials — Supplementary Data S1 is available. The predictive results of pathogenic risks of BRCA1 VUSs. [file 6667201.f1.pdf]

ID	BRCA1 VUS(Variation)	refSeq ID	Prediction	Clinical significance (
1	c.5573T>C	rs755427809	Benign	Conflicting interpretat
2	c.5511G>T	rs80356914	Benign	Conflicting interpretat
3	c.5454C>T	rs1555574705	Benign	Conflicting interpretat
4	c.5453A>G	rs80357477	Benign	Conflicting interpretat
5	c.5432A>G	rs80357040	Pathogenic	Conflicting interpretat
6	c.5419A>G	rs786202721	Benign	Conflicting interpretat
7	c.5407-25T>A	rs758780152	Benign	Conflicting interpretat
8	c.5382G>A		Pathogenic	Conflicting interpretat
9	c.5365G>T	rs80357078	Benign	Conflicting interpretat
10	c.5365G>A	rs80357078	Pathogenic	Conflicting interpretat
11	c.5359T>A	rs80357065	Benign	Conflicting interpretat
12	c.5357T>C	rs398122697	Benign	Conflicting interpretat
13	c.5348T>C	rs55808233	Benign	Conflicting interpretat
14	c.5347A>C	rs80357012	Benign	Conflicting interpretat
15	c.5333-8C>T	rs80358084	Benign	Conflicting interpretat
16	c.5332G>A	rs80357112	Pathogenic	Conflicting interpretat
17	c.5327C>A	rs398122695	Pathogenic	Conflicting interpretat
18	c.5317A>T	rs80357324	Benign	Conflicting interpretat
19	c.5306A>G	rs397509257	Pathogenic	Conflicting interpretat
20	c.5285G>A	rs398122694	Pathogenic	Conflicting interpretat
21	c.5282T>C	rs80356905	Benign	Conflicting interpretat
22	c.5277G>A	rs80356854	Pathogenic	Conflicting interpretat
23	c.5258G>C	rs397509246	Benign	Conflicting interpretat
24	c.5257A>G		Benign	Conflicting interpretat
25	c.5242G>C	rs397507245	Benign	Conflicting interpretat
26	c.5242G>T	rs397507245	Benign	Conflicting interpretat
27	c.5216A>T	rs80357227	Benign	Conflicting interpretat
28	c.5216A>G	rs80357227	Benign	Conflicting interpretat
29	c.5211A>G	rs1555576963	Pathogenic	Conflicting interpretat
30	c.5189A>G	rs80357171	Benign	Conflicting interpretat
31	c.5152+15A>G	rs750905289	Pathogenic	Conflicting interpretat
32	c.5143A>T	rs80357222	Pathogenic	Conflicting interpretat
33	c.5138T>C	rs80357132	Benign	Conflicting interpretat
34	c.5108A>G	rs876660071	Pathogenic	Conflicting interpretat
35	c.5106A>G	rs1060504574	Benign	Conflicting interpretat
36	c.5090G>A	rs397507241	Pathogenic	Conflicting interpretat
37	c.5068A>C	rs397507239	Benign	Conflicting interpretat
38	c.5014C>T	rs587781477	Benign	Conflicting interpretat
39	c.5005G>T	rs80357087	Benign	Conflicting interpretat
40	c.4993G>A	rs80357169	Benign	Conflicting interpretat
41	c.4987-5T>C	rs397509214	Benign	Conflicting interpretat
42	c.4970T>C		Benign	Conflicting interpretat
43	c.4963T>C	rs1057518639	Benign	Conflicting interpretat
44	c.4954A>T	rs1348949389	Benign	Conflicting interpretat
45	c.4941C>A	rs80357302	Benign	Conflicting interpretat
46	c.4935G>C	rs80357373	Pathogenic	Conflicting interpretat
47	c.4934G>C	rs70953661	Benign	Conflicting interpretat
48	c.4882A>G	rs80357465	Benign	Conflicting interpretat
49	c.4858A>G	rs8176219	Pathogenic	Conflicting interpretat
50	c.4837A>T	rs1799966	Pathogenic	Conflicting interpretat
51	c.4776C>G	rs761925468	Benign	Conflicting interpretat
52	c.4775A>G	rs786203699	Benign	Conflicting interpretat
53	c.4766G>A	rs80357341	Benign	Conflicting interpretat

54	c.4765C>T	rs80357002	Benign	Conflicting interpretat
55	c.4735C>G	rs145466894	Pathogenic	Conflicting interpretat
56	c.4729T>C	rs80356909	Benign	Conflicting interpretat
57	c.4683C>G	rs878853265	Benign	Conflicting interpretat
58	c.4657T>A	rs80357431	Benign	Conflicting interpretat
59	c.4654T>C	rs1265352633	Benign	Conflicting interpretat
60	c.4643C>T	rs273900737	Benign	Conflicting interpretat
61	c.4638T>G	rs397507235	Benign	Conflicting interpretat
62	c.4635C>G	rs373686790	Benign	Conflicting interpretat
63	c.4625C>G	rs41293457	Benign	Conflicting interpretat
64	c.4610A>G	rs70953659	Benign	Conflicting interpretat
65	c.4585A>G	rs80357095	Pathogenic	Conflicting interpretat
66	c.4505C>A	rs56335406	Benign	Conflicting interpretat
67	c.4410A>T	rs80357075	Pathogenic	Conflicting interpretat
68	c.4361T>C	rs587782606	Benign	Conflicting interpretat
69	c.4358-2725T>C	rs374519494	Benign	Conflicting interpretat
70	c.4352A>G	rs949793708	Benign	Conflicting interpretat
71	c.4344C>T	rs1250691798	Benign	Conflicting interpretat
72	c.4343G>A	rs80357354	Benign	Conflicting interpretat
73	c.4342A>G	rs80357486	Benign	Conflicting interpretat
74	c.4315C>T	rs781260818	Benign	Conflicting interpretat
75	c.4262A>G	rs80357079	Benign	Conflicting interpretat
76	c.4261C>T	rs80357013	Benign	Conflicting interpretat
77	c.4213A>G	rs80357353	Benign	Conflicting interpretat
78	c.4185+14G>C	rs762153716	Pathogenic	Conflicting interpretat
79	c.4184A>G	rs80356972	Pathogenic	Conflicting interpretat
80	c.4181C>T	rs397507226	Benign	Conflicting interpretat
81	c.4166G>A	rs78951648	Pathogenic	Conflicting interpretat
82	c.4127C>G	rs80356986	Benign	Conflicting interpretat
83	c.4115G>A	rs55848034	Pathogenic	Conflicting interpretat
84	c.4111G>C	rs774593602	Benign	Conflicting interpretat
85	c.4036G>A	rs80357407	Pathogenic	Conflicting interpretat
86	c.3963C>G	rs1567789440	Benign	Conflicting interpretat
87	c.3956G>A	rs587782634	Pathogenic	Conflicting interpretat
88	c.3944C>G	rs80357500	Benign	Conflicting interpretat
89	c.3929C>A	rs80357257	Benign	Conflicting interpretat
90	c.3848A>G	rs80357047	Benign	Conflicting interpretat
91	c.3845A>T	rs80357217	Pathogenic	Conflicting interpretat
92	c.3835G>A	rs80357036	Benign	Conflicting interpretat
93	c.3818A>G	rs431825400	Pathogenic	Conflicting interpretat
94	c.3815A>G	rs772703445	Benign	Conflicting interpretat
95	c.3776A>C	rs483353090	Benign	Conflicting interpretat
96	c.3750G>C	rs145903082	Benign	Conflicting interpretat
97	c.3737C>A	rs878854949	Benign	Conflicting interpretat
98	c.3722C>A	rs80357143	Pathogenic	Conflicting interpretat
99	c.3712C>T	rs876659772	Benign	Conflicting interpretat
100	c.3711A>G	rs80357388	Pathogenic	Conflicting interpretat
101	c.3707A>G	rs863224760	Benign	Conflicting interpretat
102	c.3691T>C	rs41293451	Benign	Conflicting interpretat
103	c.3596C>T	rs587782458	Benign	Conflicting interpretat
104	c.3560G>A	rs80356975	Pathogenic	Conflicting interpretat
105	c.3454G>A	rs80357175	Pathogenic	Conflicting interpretat
106	c.3410T>C	rs80357297	Benign	Conflicting interpretat
107	c.3367G>T	rs80356867	Pathogenic	Conflicting interpretat

108	c.3362A>G	rs80356919	Benign	Conflicting interpretat
109	c.3355A>T	rs80356949	Pathogenic	Conflicting interpretat
110	c.3346G>C	rs55909400	Benign	Conflicting interpretat
111	c.3270A>T	rs369925993	Benign	Conflicting interpretat
112	c.3247A>G	rs397507213	Pathogenic	Conflicting interpretat
113	c.3211G>A	rs41293445	Pathogenic	Conflicting interpretat
114	c.3206A>C	rs879254151	Benign	Conflicting interpretat
115	c.3181A>G	rs876658975	Benign	Conflicting interpretat
116	c.3179A>C	rs80357184	Benign	Conflicting interpretat
117	c.3113A>C	rs16941	Benign	Conflicting interpretat
118	c.3055A>G	rs80357311	Benign	Conflicting interpretat
119	c.3041T>C	rs80357020	Benign	Conflicting interpretat
120	c.3027A>C	rs1555588512	Benign	Conflicting interpretat
121	c.3008T>C		Benign	Conflicting interpretat
122	c.2987A>G	rs786202898	Benign	Conflicting interpretat
123	c.2964A>G	rs1064796061	Pathogenic	Conflicting interpretat
124	c.2963C>T	rs397507206	Benign	Conflicting interpretat
125	c.2936G>A	rs80356985	Benign	Conflicting interpretat
126	c.2935C>T	rs80356970	Benign	Conflicting interpretat
127	c.2910A>C	rs431825394	Benign	Conflicting interpretat
128	c.2773A>G	rs4986847	Pathogenic	Conflicting interpretat
129	c.2735A>G	rs397507204	Benign	Conflicting interpretat
130	c.2726A>T	rs80357127	Benign	Conflicting interpretat
131	c.2668G>A	rs80357200	Pathogenic	Conflicting interpretat
132	c.2666C>T	rs769712441	Benign	Conflicting interpretat
133	c.2662C>T	rs80357480	Benign	Conflicting interpretat
134	c.2657C>G	rs587782134	Benign	Conflicting interpretat
135	c.2630A>G	rs786203689	Benign	Conflicting interpretat
136	c.2612C>G	rs799917	Benign	Conflicting interpretat
137	c.2518A>T	rs377475866	Pathogenic	Conflicting interpretat
138	c.2500G>C	rs786202215	Benign	Conflicting interpretat
139	c.2481A>G	rs397508970	Benign	Conflicting interpretat
140	c.2481A>C	rs397508970	Benign	Conflicting interpretat
141	c.2473G>T	rs80357328	Pathogenic	Conflicting interpretat
142	c.2447A>G	rs80357108	Pathogenic	Conflicting interpretat
143	c.2426A>G	rs397507201	Benign	Conflicting interpretat
144	c.2416G>A	rs80357144	Pathogenic	Conflicting interpretat
145	c.2393C>T	rs876660005	Benign	Conflicting interpretat
146	c.2368A>G	rs41286298	Benign	Conflicting interpretat
147	c.2347A>G	rs80356948	Pathogenic	Conflicting interpretat
148	c.2329T>G	rs397507199	Pathogenic	Conflicting interpretat
149	c.2268G>T	rs80356884	Benign	Conflicting interpretat
150	c.2268G>C	rs80356884	Pathogenic	Conflicting interpretat
151	c.2264A>G	rs922908090	Benign	Conflicting interpretat
152	c.2252T>C	rs587781684	Benign	Conflicting interpretat
153	c.2224A>G	rs876658733	Pathogenic	Conflicting interpretat
154	c.2222C>T	rs80357051	Benign	Conflicting interpretat
155	c.2207A>C	rs397507196	Benign	Conflicting interpretat
156	c.2155A>G	rs80357147	Pathogenic	Conflicting interpretat
157	c.2123C>T	rs80357182	Benign	Conflicting interpretat
158	c.2123C>A	rs80357182	Pathogenic	Conflicting interpretat
159	c.2086A>G	rs80357441	Pathogenic	Conflicting interpretat
160	c.2083G>A	rs28897681	Benign	Conflicting interpretat
161	c.2060A>C	rs28897680	Benign	Conflicting interpretat

162	c.2043T>G	rs143920945	Benign	Conflicting interpretat
163	c.2006T>C	rs80356895	Benign	Conflicting interpretat
164	c.1974G>C	rs55678461	Benign	Conflicting interpretat
165	c.1960A>C	rs80357355	Benign	Conflicting interpretat
166	c.1920A>G	rs587782843	Benign	Conflicting interpretat
167	c.1907G>A	rs398122649	Pathogenic	Conflicting interpretat
168	c.1897C>T	rs80356902	Benign	Conflicting interpretat
169	c.1879G>A	rs80357425	Pathogenic	Conflicting interpretat
170	c.1849A>G	rs45564238	Pathogenic	Conflicting interpretat
171	c.1837A>G	rs863224753	Pathogenic	Conflicting interpretat
172	c.1805A>G	rs1567798189	Benign	Conflicting interpretat
173	c.1802A>G	rs371631805	Benign	Conflicting interpretat
174	c.1775G>A	rs786203044	Pathogenic	Conflicting interpretat
175	c.1772T>C	rs80356859	Benign	Conflicting interpretat
176	c.1747A>G	rs80356928	Benign	Conflicting interpretat
177	c.1745C>T	rs786202386	Benign	Conflicting interpretat
178	c.1724A>G	rs111539978	Benign	Conflicting interpretat
179	c.1713A>G	rs552505690	Pathogenic	Conflicting interpretat
180	c.1712T>C	rs80357159	Benign	Conflicting interpretat
181	c.1609A>G	rs398122639	Pathogenic	Conflicting interpretat
182	c.1601A>G	rs80357173	Pathogenic	Conflicting interpretat
183	c.1581G>C	rs80357493	Benign	Conflicting interpretat
184	c.1573G>A	rs80357273	Benign	Conflicting interpretat
185	c.1561G>A	rs80357122	Pathogenic	Conflicting interpretat
186	c.1560A>G	rs1555591570	Benign	Conflicting interpretat
187	c.1508A>G	rs62625304	Benign	Conflicting interpretat
188	c.1459G>T	rs369588942	Benign	Conflicting interpretat
189	c.1457T>C		Benign	Conflicting interpretat
190	c.1456T>A	rs55906931	Benign	Conflicting interpretat
191	c.1441C>G	rs1397842308	Pathogenic	Conflicting interpretat
192	c.1405G>A	rs397507187	Benign	Conflicting interpretat
193	c.1400A>G	rs876659316	Benign	Conflicting interpretat
194	c.1397G>A	rs199540030	Benign	Conflicting interpretat
195	c.1396C>T	rs80356964	Benign	Conflicting interpretat
196	c.1357G>C	rs768054411	Benign	Conflicting interpretat
197	c.1258G>T	rs80357488	Pathogenic	Conflicting interpretat
198	c.1253A>C	rs1555592050	Benign	Conflicting interpretat
199	c.1250A>G	rs80357113	Benign	Conflicting interpretat
200	c.1243G>A	rs587782770	Benign	Conflicting interpretat
201	c.1233T>C	rs80357024	Benign	Conflicting interpretat
202	c.1222A>G	rs80357253	Benign	Conflicting interpretat
203	c.1202G>A	rs397507184	Benign	Conflicting interpretat
204	c.1081T>C	rs80356946	Benign	Conflicting interpretat
205	c.1026G>A	rs1171571879	Pathogenic	Conflicting interpretat
206	c.1016A>G	rs587781737	Benign	Conflicting interpretat
207	c.1015A>G	rs55842957	Benign	Conflicting interpretat
208	c.1011A>G	rs1555592670	Benign	Conflicting interpretat
209	c.1008A>G	rs1060504568	Pathogenic	Conflicting interpretat
210	c.995G>A	rs80357464	Benign	Conflicting interpretat
211	c.994C>T	rs80357176	Benign	Conflicting interpretat
212	c.964G>C	rs80357252	Benign	Conflicting interpretat
213	c.964G>A	rs80357252	Pathogenic	Conflicting interpretat
214	c.889A>C	rs80357196	Benign	Conflicting interpretat
215	c.886A>G	rs748675395	Pathogenic	Conflicting interpretat

216	c.811G>A	rs80357244	Pathogenic	Conflicting interpretat
217	c.755G>A	rs80357138	Benign	Conflicting interpretat
218	c.754C>T	rs273902786	Benign	Conflicting interpretat
219	c.742A>C	rs879255288	Benign	Conflicting interpretat
220	c.707C>T		Benign	Conflicting interpretat
221	c.697G>A	rs959797914	Pathogenic	Conflicting interpretat
222	c.694G>A	rs55975699	Benign	Conflicting interpretat
223	c.692C>T	rs80357001	Benign	Conflicting interpretat
224	c.672T>G	rs1064794486	Benign	Conflicting interpretat
225	c.661G>T	rs80357088	Benign	Conflicting interpretat
226	c.655G>A	rs273902779	Pathogenic	Conflicting interpretat
227	c.630A>G	rs1555593567	Benign	Conflicting interpretat
228	c.612G>C	rs80357394	Benign	Conflicting interpretat
229	c.572T>A	rs80357142	Benign	Conflicting interpretat
230	c.512T>C		Benign	Conflicting interpretat
231	c.509G>A	rs80357264	Benign	Conflicting interpretat
232	c.486G>A	rs769213707	Pathogenic	Conflicting interpretat
233	c.484G>C	rs55816927	Benign	Conflicting interpretat
234	c.482C>T	rs876660138	Benign	Conflicting interpretat
235	c.457A>C	rs28897674	Benign	Conflicting interpretat
236	c.453T>C	rs1555594986	Benign	Conflicting interpretat
237	c.446A>C	rs397507233	Benign	Conflicting interpretat
238	c.441G>C	rs748876625	Benign	Conflicting interpretat
239	c.423A>G	rs777102216	Benign	Conflicting interpretat
240	c.398G>A	rs80357357	Benign	Conflicting interpretat
241	c.389A>T	rs56055578	Benign	Conflicting interpretat
242	c.301+1G>A	rs587782173	Pathogenic	Conflicting interpretat
243	c.154C>A	rs80357084	Pathogenic	Conflicting interpretat
244	c.154C>T	rs80357084	Benign	Conflicting interpretat
245	c.144G>A	rs587783040	Pathogenic	Conflicting interpretat
246	c.135-5T>C	rs587781916	Benign	Conflicting interpretat
247	c.132C>T	rs876658362	Pathogenic	Conflicting interpretat
248	c.124A>G	rs80357163	Benign	Conflicting interpretat
249	c.122A>T	rs80357276	Benign	Conflicting interpretat
250	c.121C>A	rs1060502353	Benign	Conflicting interpretat
251	c.93C>G	rs80357000	Pathogenic	Conflicting interpretat
252	c.81T>C	rs587780805	Benign	Conflicting interpretat
253	c.81-6T>C	rs80358179	Benign	Conflicting interpretat
254	c.80G>A		Benign	Conflicting interpretat
255	c.80G>T	rs1064793052	Benign	Conflicting interpretat
256	c.70T>A		Benign	Conflicting interpretat
257	c.43A>C	rs80357031	Benign	Conflicting interpretat
258	c.22G>A	rs528902306	Benign	Conflicting interpretat
259	c.20G>A	rs144792613	Benign	Conflicting interpretat
260	c.19C>T	rs80356994	Benign	Conflicting interpretat
261	c.10T>C	rs876658707	Benign	Conflicting interpretat
262	c.-10A>C	rs748057929	Benign	Conflicting interpretat
263	c.-16A>G	rs777262055	Pathogenic	Conflicting interpretat
264	c.-20+5T>C	rs1057524628	Benign	Conflicting interpretat
265	c.*1286C>T	rs548275991	Benign	Likely benign
266	c.*750A>G	rs138782023	Pathogenic	Likely benign
267	c.*528G>C	rs1060504556	Pathogenic	Likely benign
268	c.*20C>T	rs375042815	Benign	Likely benign
269	c.*4C>T	rs1057520246	Pathogenic	Likely benign

270	c.*3G>T	rs1057521525	Benign	Likely benign
271	c.*1C>T	rs587782097	Benign	Likely benign
272	c.5586C>T	rs774127304	Benign	Likely benign
273	c.5574C>T	rs876659941	Benign	Likely benign
274	c.5568C>G	rs876659994	Benign	Likely benign
275	c.5568C>A	rs876659994	Pathogenic	Likely benign
276	c.5566C>T	rs80357274	Benign	Likely benign
277	c.5565A>C	rs758449088	Benign	Likely benign
278	c.5562G>A	rs786201648	Pathogenic	Likely benign
279	c.5556C>G	rs80356841	Benign	Likely benign
280	c.5553C>T	rs80357326	Benign	Likely benign
281	c.5550G>A	rs786201502	Pathogenic	Likely benign
282	c.5550G>C	rs786201502	Benign	Likely benign
283	c.5547G>A	rs1426388214	Pathogenic	Likely benign
284	c.5541C>T	rs397509295	Benign	Likely benign
285	c.5538G>A	rs80356849	Pathogenic	Likely benign
286	c.5532C>T	rs80356829	Benign	Likely benign
287	c.5529A>G	rs1160863525	Benign	Likely benign
288	c.5529A>C	rs1160863525	Benign	Likely benign
289	c.5526A>G	rs1555574397	Pathogenic	Likely benign
290	c.5523T>C	rs878854960	Benign	Likely benign
291	c.5514G>T	rs786201248	Benign	Likely benign
292	c.5502C>T	rs1057520459	Benign	Likely benign
293	c.5496G>A	rs1131692070	Pathogenic	Likely benign
294	c.5478G>A	rs80357332	Pathogenic	Likely benign
295	c.5475G>T	rs1057520941	Benign	Likely benign
296	c.5475G>A	rs1057520941	Benign	Likely benign
297	c.5469A>C		Benign	Likely benign
298	c.5468-8G>T		Benign	Likely benign
299	c.5468-49C>T		Benign	Likely benign
300	c.5466T>C	rs886052975	Benign	Likely benign
301	c.5457T>C		Benign	Likely benign
302	c.5456A>G	rs80357286	Benign	Likely benign
303	c.5451G>A	rs1057523707	Pathogenic	Likely benign
304	c.5448A>G	rs397509285	Pathogenic	Likely benign
305	c.5439T>C	rs760396669	Benign	Likely benign
306	c.5430G>A	rs786201582	Pathogenic	Likely benign
307	c.5424G>C	rs1555574748	Benign	Likely benign
308	c.5418A>C	rs1555574766	Benign	Likely benign
309	c.5416C>G	rs80357241	Benign	Likely benign
310	c.5415C>T	rs1060504559	Benign	Likely benign
311	c.5412C>T	rs730881456	Benign	Likely benign
312	c.5407-17C>G		Benign	Likely benign
313	c.5406A>C	rs879255493	Benign	Likely benign
314	c.5402G>A	rs531210457	Benign	Likely benign
315	c.5397C>T	rs1131692096	Benign	Likely benign
316	c.5394C>T	rs777371808	Benign	Likely benign
317	c.5390C>T	rs879255492	Benign	Likely benign
318	c.5388A>G	rs373810778	Pathogenic	Likely benign
319	c.5382G>C	rs397509275	Benign	Likely benign
320	c.5376G>A	rs1060504569	Pathogenic	Likely benign
321	c.5367T>C		Benign	Likely benign
322	c.5358G>C	rs1057522271	Benign	Likely benign
323	c.5356C>T	rs1555575167	Benign	Likely benign

324	c.5352A>T	rs767459025	Benign	Likely benign
325	c.5352A>G	rs767459025	Pathogenic	Likely benign
326	c.5340G>T		Benign	Likely benign
327	c.5340G>A	rs766700840	Pathogenic	Likely benign
328	c.5337A>G	rs876659718	Benign	Likely benign
329	c.5334T>C	rs754152768	Benign	Likely benign
330	c.5333A>G	rs80357041	Benign	Likely benign
331	c.5333-12T>C		Benign	Likely benign
332	c.5333-873T>C		Benign	Likely benign
333	c.5332+13G>T	rs372391060	Pathogenic	Likely benign
334	c.5332+11G>A		Pathogenic	Likely benign
335	c.5328C>G		Benign	Likely benign
336	c.5328C>T	rs759867616	Benign	Likely benign
337	c.5319C>G	rs1555575689	Benign	Likely benign
338	c.5313C>T	rs1131692076	Benign	Likely benign
339	c.5310G>C	rs273901761	Benign	Likely benign
340	c.5310G>A	rs273901761	Benign	Likely benign
341	c.5304C>T	rs138493864	Benign	Likely benign
342	c.5289G>A	rs1022076404	Pathogenic	Likely benign
343	c.5289G>C	rs1022076404	Benign	Likely benign
344	c.5280C>T	rs750040616	Benign	Likely benign
345	c.5280C>A	rs750040616	Pathogenic	Likely benign
346	c.5278-19T>A		Benign	Likely benign
347	c.5277+98T>C		Benign	Likely benign
348	c.5274A>G	rs758739620	Benign	Likely benign
349	c.5268G>A	rs571834423	Benign	Likely benign
350	c.5259A>G	rs771577266	Pathogenic	Likely benign
351	c.5256A>C	rs80356844	Benign	Likely benign
352	c.5251C>A	rs80357123	Benign	Likely benign
353	c.5250G>A		Benign	Likely benign
354	c.5202T>C		Benign	Likely benign
355	c.5196T>C	rs1567764670	Benign	Likely benign
356	c.5194-34C>T		Benign	Likely benign
357	c.5193+22C>T	rs8176260	Benign	Likely benign
358	c.5193+8C>A		Benign	Likely benign
359	c.5193G>A	rs876660702	Pathogenic	Likely benign
360	c.5185C>T	rs1207677103	Benign	Likely benign
361	c.5176A>G	rs80357501	Benign	Likely benign
362	c.5175A>G	rs191373374	Benign	Likely benign
363	c.5163G>A	rs1403122031	Benign	Likely benign
364	c.5160C>G	rs376736915	Benign	Likely benign
365	c.5157G>T	rs28897697	Pathogenic	Likely benign
366	c.5157G>A	rs28897697	Pathogenic	Likely benign
367	c.5153-31A>T		Benign	Likely benign
368	c.5152+13C>T		Benign	Likely benign
369	c.5142T>G	rs749319480	Benign	Likely benign
370	c.5139A>G	rs1555578550	Pathogenic	Likely benign
371	c.5124G>A	rs1057520432	Pathogenic	Likely benign
372	c.5115A>C	rs772885662	Benign	Likely benign
373	c.5113C>T	rs80356858	Benign	Likely benign
374	c.5109T>C	rs80356974	Benign	Likely benign
375	c.5103G>A	rs1060504591	Pathogenic	Likely benign
376	c.5100A>G	rs45519437	Benign	Likely benign
377	c.5095C>A	rs55770810	Benign	Likely benign

378	c.5094A>G	rs764891781	Benign	Likely benign
379	c.5088G>A	rs878854956	Pathogenic	Likely benign
380	c.5075-18G>T		Benign	Likely benign
381	c.5074+20A>G		Pathogenic	Likely benign
382	c.5061T>C	rs1555579625	Benign	Likely benign
383	c.5058T>C	rs397509218	Benign	Likely benign
384	c.5055T>G	rs1060504555	Benign	Likely benign
385	c.5052T>G		Benign	Likely benign
386	c.5052T>A	rs760922019	Benign	Likely benign
387	c.5052T>C	rs760922019	Benign	Likely benign
388	c.5037A>G	rs754256578	Pathogenic	Likely benign
389	c.5034T>C	rs898511378	Benign	Likely benign
390	c.5031T>A	rs765241963	Benign	Likely benign
391	c.5028A>G	rs1064793596	Pathogenic	Likely benign
392	c.5026T>C	rs1567772244	Benign	Likely benign
393	c.5025T>C	rs876658226	Benign	Likely benign
394	c.5023A>G	rs774452090	Pathogenic	Likely benign
395	c.5022C>T	rs786203868	Benign	Likely benign
396	c.5010A>G	rs777828258	Benign	Likely benign
397	c.5008A>C	rs1057523976	Benign	Likely benign
398	c.5007C>T	rs751856943	Benign	Likely benign
399	c.5001G>A	rs889937720	Benign	Likely benign
400	c.4998C>T	rs730882165	Benign	Likely benign
401	c.4987-8A>G		Pathogenic	Likely benign
402	c.4987-20A>C		Benign	Likely benign
403	c.4977A>C	rs1131692087	Benign	Likely benign
404	c.4974C>T	rs1555580615	Benign	Likely benign
405	c.4971G>C	rs786202058	Benign	Likely benign
406	c.4971G>A	rs786202058	Pathogenic	Likely benign
407	c.4962G>A	rs549640262	Pathogenic	Likely benign
408	c.4959G>A	rs878854955	Pathogenic	Likely benign
409	c.4930G>C		Benign	Likely benign
410	c.4929A>C	rs786202022	Benign	Likely benign
411	c.4923T>G	rs876658258	Benign	Likely benign
412	c.4917G>A	rs1060504558	Pathogenic	Likely benign
413	c.4915T>C		Benign	Likely benign
414	c.4914A>G	rs786201216	Benign	Likely benign
415	c.4908G>A	rs1567774705	Pathogenic	Likely benign
416	c.4902G>A	rs746199881	Benign	Likely benign
417	c.4899C>T		Benign	Likely benign
418	c.4893T>C	rs80356850	Benign	Likely benign
419	c.4881A>G	rs1425743425	Benign	Likely benign
420	c.4872G>T		Benign	Likely benign
421	c.4872G>A	rs11555992	Pathogenic	Likely benign
422	c.4860T>C	rs750938749	Benign	Likely benign
423	c.4854T>C	rs750718476	Benign	Likely benign
424	c.4851T>C	rs786202627	Benign	Likely benign
425	c.4848T>C		Benign	Likely benign
426	c.4845T>C	rs144588397	Benign	Likely benign
427	c.4833C>T	rs80356842	Benign	Likely benign
428	c.4830T>G	rs1555580852	Benign	Likely benign
429	c.4824A>G	rs1555580860	Pathogenic	Likely benign
430	c.4821T>C	rs1260740618	Benign	Likely benign
431	c.4815G>A	rs1060504589	Pathogenic	Likely benign

432	c.4813T>C	rs80356833	Benign	Likely benign
433	c.4803A>G	rs886037794	Benign	Likely benign
434	c.4798T>C	rs775837744	Benign	Likely benign
435	c.4791C>T	rs1555580935	Benign	Likely benign
436	c.4782A>G	rs876659455	Benign	Likely benign
437	c.4767T>A		Benign	Likely benign
438	c.4767T>G	rs587780864	Benign	Likely benign
439	c.4764T>A	rs753651115	Pathogenic	Likely benign
440	c.4764T>C	rs753651115	Benign	Likely benign
441	c.4752C>T	rs786201422	Benign	Likely benign
442	c.4750G>T	rs80357070	Benign	Likely benign
443	c.4749A>G		Pathogenic	Likely benign
444	c.4743A>G	rs397509194	Benign	Likely benign
445	c.4740T>C	rs777297026	Benign	Likely benign
446	c.4737T>C	rs878854954	Benign	Likely benign
447	c.4731T>A	rs1555581028	Benign	Likely benign
448	c.4726G>C	rs1060502355	Benign	Likely benign
449	c.4725T>G	rs878854953	Benign	Likely benign
450	c.4719T>C	rs1057520436	Benign	Likely benign
451	c.4710C>T	rs786201839	Benign	Likely benign
452	c.4690C>T		Benign	Likely benign
453	c.4689C>T	rs80357433	Benign	Likely benign
454	c.4683C>A	rs878853265	Pathogenic	Likely benign
455	c.4683C>T	rs878853265	Benign	Likely benign
456	c.4682C>T	rs56158747	Benign	Likely benign
457	c.4676-44A>C		Benign	Likely benign
458	c.4675+7T>A		Benign	Likely benign
459	c.4665G>A	rs878854952	Benign	Likely benign
460	c.4657T>C	rs80357431	Benign	Likely benign
461	c.4653T>C	rs587780863	Benign	Likely benign
462	c.4650A>T	rs876658608	Benign	Likely benign
463	c.4650A>G	rs876658608	Benign	Likely benign
464	c.4644G>A	rs28897692	Benign	Likely benign
465	c.4635C>T	rs373686790	Benign	Likely benign
466	c.4629G>A	rs1555581858	Benign	Likely benign
467	c.4626T>C	rs786202425	Benign	Likely benign
468	c.4620A>G	rs769566412	Benign	Likely benign
469	c.4617G>A		Pathogenic	Likely benign
470	c.4614G>A	rs1555581876	Pathogenic	Likely benign
471	c.4599T>C	rs1131692095	Benign	Likely benign
472	c.4596T>C	rs1567777953	Benign	Likely benign
473	c.4584C>T	rs1131692080	Benign	Likely benign
474	c.4581G>A	rs1060504560	Pathogenic	Likely benign
475	c.4565A>G	rs80357379	Pathogenic	Likely benign
476	c.4560A>G	rs761293595	Benign	Likely benign
477	c.4557T>C	rs876659243	Benign	Likely benign
478	c.4554G>A	rs786202635	Pathogenic	Likely benign
479	c.4545G>A	rs755731300	Pathogenic	Likely benign
480	c.4545G>T	rs755731300	Pathogenic	Likely benign
481	c.4544G>A	rs398122688	Pathogenic	Likely benign
482	c.4527C>T	rs886040233	Benign	Likely benign
483	c.4521G>A	rs1435385135	Pathogenic	Likely benign
484	c.4518T>C	rs73983787	Benign	Likely benign
485	c.4515T>C	rs1057522167	Benign	Likely benign

486	c.4512A>G	rs1555582056	Pathogenic	Likely benign
487	c.4506A>G	rs778400777	Benign	Likely benign
488	c.4503C>T	rs747539984	Benign	Likely benign
489	c.4491C>T		Benign	Likely benign
490	c.4485-11T>A		Benign	Likely benign
491	c.4485-14A>T		Benign	Likely benign
492	c.4484+32A>G		Pathogenic	Likely benign
493	c.4443A>C	rs1555582589	Benign	Likely benign
494	c.4437G>A	rs1232117701	Pathogenic	Likely benign
495	c.4428G>A	rs1057522527	Benign	Likely benign
496	c.4422T>C	rs756281673	Benign	Likely benign
497	c.4419T>A	rs730881455	Benign	Likely benign
498	c.4416T>C	rs780239567	Benign	Likely benign
499	c.4413C>T	rs749522068	Benign	Likely benign
500	c.4392T>A	rs794727102	Benign	Likely benign
501	c.4389C>T		Benign	Likely benign
502	c.4380T>C	rs786203100	Benign	Likely benign
503	c.4368T>G	rs1171220106	Benign	Likely benign
504	c.4362A>G		Pathogenic	Likely benign
505	c.4358-10C>T	rs80358111	Benign	Likely benign
506	c.4358-20T>G		Benign	Likely benign
507	c.4358-37G>A		Pathogenic	Likely benign
508	c.4358-48A>G		Pathogenic	Likely benign
509	c.4357+20C>T		Benign	Likely benign
510	c.4353A>G	rs786202387	Benign	Likely benign
511	c.4347A>G	rs80356840	Benign	Likely benign
512	c.4338A>G		Benign	Likely benign
513	c.4335A>C	rs1555584033	Benign	Likely benign
514	c.4332T>C	rs752824502	Benign	Likely benign
515	c.4328G>A	rs4986849	Benign	Likely benign
516	c.4323C>T	rs1057523168	Benign	Likely benign
517	c.4314C>G	rs80356856	Benign	Likely benign
518	c.4311T>C	rs1131692092	Benign	Likely benign
519	c.4308T>G		Benign	Likely benign
520	c.4305C>T	rs730881445	Benign	Likely benign
521	c.4294A>C	rs80357157	Benign	Likely benign
522	c.4287C>T	rs397509160	Benign	Likely benign
523	c.4272G>A	rs398122684	Pathogenic	Likely benign
524	c.4269C>T	rs786202278	Benign	Likely benign
525	c.4266G>A	rs1555584142	Pathogenic	Likely benign
526	c.4263T>C	rs1131692074	Benign	Likely benign
527	c.4251G>A	rs777057839	Pathogenic	Likely benign
528	c.4245A>G	rs41293453	Benign	Likely benign
529	c.4242A>G	rs1057521982	Pathogenic	Likely benign
530	c.4230A>G	rs1555584217	Benign	Likely benign
531	c.4227G>A	rs786201618	Benign	Likely benign
532	c.4218G>A	rs1800707	Benign	Likely benign
533	c.4215A>T	rs1555584242	Benign	Likely benign
534	c.4212G>C		Benign	Likely benign
535	c.4212G>A		Pathogenic	Likely benign
536	c.4209C>T	rs786201224	Benign	Likely benign
537	c.4197C>G	rs876659552	Benign	Likely benign
538	c.4191G>A	rs1555584270	Benign	Likely benign
539	c.4186-128A>T		Pathogenic	Likely benign

540	c.4185+40T>G		Benign	Likely benign
541	c.4185+20G>A		Pathogenic	Likely benign
542	c.4182T>C	rs864622540	Benign	Likely benign
543	c.4179C>G	rs753735698	Benign	Likely benign
544	c.4179C>T	rs753735698	Benign	Likely benign
545	c.4155A>G	rs1057520245	Pathogenic	Likely benign
546	c.4146C>T	rs1057517574	Benign	Likely benign
547	c.4137T>C	rs1057521866	Benign	Likely benign
548	c.4134C>T		Benign	Likely benign
549	c.4131C>T	rs80356871	Benign	Likely benign
550	c.4113G>T	rs147448807	Benign	Likely benign
551	c.4113G>A	rs147448807	Pathogenic	Likely benign
552	c.4110T>C	rs1131692067	Benign	Likely benign
553	c.4107A>G	rs1555586263	Benign	Likely benign
554	c.4097-11T>G		Benign	Likely benign
555	c.4096+43G>A		Pathogenic	Likely benign
556	c.4092C>T	rs786201566	Benign	Likely benign
557	c.4081A>G	rs80357218	Benign	Likely benign
558	c.4074G>A	rs80356846	Pathogenic	Likely benign
559	c.4071A>G	rs786201475	Benign	Likely benign
560	c.4051T>C	rs139858874	Benign	Likely benign
561	c.4050C>T	rs779507799	Benign	Likely benign
562	c.4047G>T	rs758515222	Benign	Likely benign
563	c.4047G>A	rs758515222	Benign	Likely benign
564	c.4026A>C	rs80356828	Benign	Likely benign
565	c.4026A>G	rs80356828	Pathogenic	Likely benign
566	c.4020G>A		Pathogenic	Likely benign
567	c.4018T>C	rs786201646	Benign	Likely benign
568	c.4014G>A	rs1555586655	Benign	Likely benign
569	c.4002T>C	rs1131692083	Benign	Likely benign
570	c.3995G>A		Pathogenic	Likely benign
571	c.3993G>A	rs70953658	Pathogenic	Likely benign
572	c.3990C>T	rs876660808	Benign	Likely benign
573	c.3975G>A	rs761424661	Benign	Likely benign
574	c.3960T>C		Benign	Likely benign
575	c.3951G>A	rs1060504566	Pathogenic	Likely benign
576	c.3936C>T	rs753210219	Benign	Likely benign
577	c.3935C>G	rs1555586769	Benign	Likely benign
578	c.3930A>C	rs1555586780	Benign	Likely benign
579	c.3918G>A	rs786202068	Pathogenic	Likely benign
580	c.3909G>A		Pathogenic	Likely benign
581	c.3900C>T	rs730881454	Pathogenic	Likely benign
582	c.3897G>A	rs398122678	Pathogenic	Likely benign
583	c.3891T>C	rs1555586847	Benign	Likely benign
584	c.3885G>A	rs1057522176	Pathogenic	Likely benign
585	c.3879T>C	rs1057523579	Benign	Likely benign
586	c.3867A>G		Benign	Likely benign
587	c.3858T>C	rs794726998	Benign	Likely benign
588	c.3852C>T	rs776070899	Benign	Likely benign
589	c.3849T>C	rs1567789838	Benign	Likely benign
590	c.3846A>G	rs1555586947	Benign	Likely benign
591	c.3834G>A	rs876660942	Benign	Likely benign
592	c.3826T>G	rs876659178	Benign	Likely benign
593	c.3826T>C	rs876659178	Benign	Likely benign

594	c.3822A>C	rs372396487	Benign	Likely benign
595	c.3810C>T	rs886040166	Pathogenic	Likely benign
596	c.3807C>T	rs786202569	Benign	Likely benign
597	c.3804T>C	rs140588714	Benign	Likely benign
598	c.3799T>C		Benign	Likely benign
599	c.3798C>T	rs200648498	Benign	Likely benign
600	c.3792G>A	rs894781955	Pathogenic	Likely benign
601	c.3789G>A		Pathogenic	Likely benign
602	c.3787T>C	rs1057523961	Benign	Likely benign
603	c.3783A>G	rs80356831	Pathogenic	Likely benign
604	c.3780A>G	rs753081589	Pathogenic	Likely benign
605	c.3774G>A	rs431825399	Pathogenic	Likely benign
606	c.3771G>A		Pathogenic	Likely benign
607	c.3768A>G	rs786202803	Pathogenic	Likely benign
608	c.3759T>G	rs80356852	Benign	Likely benign
609	c.3756G>C	rs752122039	Benign	Likely benign
610	c.3756G>A	rs752122039	Pathogenic	Likely benign
611	c.3750G>A	rs145903082	Pathogenic	Likely benign
612	c.3747C>A		Benign	Likely benign
613	c.3747C>G	rs587780801	Benign	Likely benign
614	c.3747C>T	rs587780801	Benign	Likely benign
615	c.3744T>A	rs1555587190	Pathogenic	Likely benign
616	c.3738C>T	rs778655093	Benign	Likely benign
617	c.3735C>T	rs1567790391	Benign	Likely benign
618	c.3726T>C	rs1060504567	Benign	Likely benign
619	c.3723T>C	rs772759939	Benign	Likely benign
620	c.3720G>A	rs876658341	Benign	Likely benign
621	c.3717T>C		Benign	Likely benign
622	c.3717T>A	rs730881453	Benign	Likely benign
623	c.3714T>G		Benign	Likely benign
624	c.3714T>C	rs140777892	Benign	Likely benign
625	c.3708T>C	rs28897687	Benign	Likely benign
626	c.3702A>G	rs587780862	Pathogenic	Likely benign
627	c.3699A>G	rs368690455	Benign	Likely benign
628	c.3690A>G		Pathogenic	Likely benign
629	c.3688T>C	rs786201581	Benign	Likely benign
630	c.3685T>C	rs767958299	Benign	Likely benign
631	c.3672C>T		Benign	Likely benign
632	c.3672C>G	rs1555587298	Benign	Likely benign
633	c.3657G>A	rs80356876	Pathogenic	Likely benign
634	c.3645C>T	rs758329415	Benign	Likely benign
635	c.3642G>A	rs398122675	Pathogenic	Likely benign
636	c.3636A>T	rs148038877	Pathogenic	Likely benign
637	c.3636A>G	rs148038877	Pathogenic	Likely benign
638	c.3627A>G	rs770579978	Pathogenic	Likely benign
639	c.3621G>A		Pathogenic	Likely benign
640	c.3615G>A	rs750113197	Benign	Likely benign
641	c.3612A>G	rs537737635	Pathogenic	Likely benign
642	c.3606C>T		Benign	Likely benign
643	c.3603T>C	rs80356830	Benign	Likely benign
644	c.3603T>G	rs80356830	Benign	Likely benign
645	c.3601G>A	rs55725337	Pathogenic	Likely benign
646	c.3600G>C	rs56214134	Benign	Likely benign
647	c.3597T>A	rs1555587434	Pathogenic	Likely benign

648	c.3588A>C	rs876658595	Benign	Likely benign
649	c.3588A>G	rs876658595	Benign	Likely benign
650	c.3582C>T	rs786202722	Benign	Likely benign
651	c.3576T>C	rs766447664	Benign	Likely benign
652	c.3573C>T	rs864622080	Benign	Likely benign
653	c.3561C>T	rs1555587501	Benign	Likely benign
654	c.3551G>A	rs1567791181	Pathogenic	Likely benign
655	c.3540C>T	rs928545955	Benign	Likely benign
656	c.3537A>G	rs1131692090	Benign	Likely benign
657	c.3531T>C		Benign	Likely benign
658	c.3525T>G	rs1131692086	Benign	Likely benign
659	c.3519T>C		Benign	Likely benign
660	c.3513G>A	rs786202844	Benign	Likely benign
661	c.3498T>C	rs1298897645	Benign	Likely benign
662	c.3495T>C	rs1555587585	Benign	Likely benign
663	c.3486T>C	rs1555587617	Benign	Likely benign
664	c.3474A>G	rs1567791508	Benign	Likely benign
665	c.3468T>C	rs864622146	Benign	Likely benign
666	c.3465T>C	rs1567791539	Benign	Likely benign
667	c.3462A>G	rs876659048	Pathogenic	Likely benign
668	c.3438T>C	rs1555587693	Benign	Likely benign
669	c.3435T>C	rs786201222	Benign	Likely benign
670	c.3426A>G	rs80356843	Benign	Likely benign
671	c.3423T>C	rs863224419	Benign	Likely benign
672	c.3402A>G	rs752875919	Benign	Likely benign
673	c.3396C>T	rs764013144	Benign	Likely benign
674	c.3390A>G	rs757237039	Pathogenic	Likely benign
675	c.3381T>C	rs781319410	Benign	Likely benign
676	c.3378A>G	rs1057521325	Benign	Likely benign
677	c.3372C>T	rs786203431	Benign	Likely benign
678	c.3357T>C	rs772383323	Benign	Likely benign
679	c.3357T>A	rs772383323	Benign	Likely benign
680	c.3354G>A	rs80357334	Pathogenic	Likely benign
681	c.3348A>G	rs1131692073	Pathogenic	Likely benign
682	c.3339T>C	rs80357421	Benign	Likely benign
683	c.3327A>G	rs41293449	Benign	Likely benign
684	c.3315T>C		Benign	Likely benign
685	c.3312G>A	rs876659024	Pathogenic	Likely benign
686	c.3306T>C	rs876658664	Benign	Likely benign
687	c.3279C>G	rs1567792257	Benign	Likely benign
688	c.3270A>G	rs369925993	Benign	Likely benign
689	c.3267G>A	rs767544239	Pathogenic	Likely benign
690	c.3252T>G	rs1567792354	Benign	Likely benign
691	c.3246T>A		Pathogenic	Likely benign
692	c.3243T>C	rs876659805	Benign	Likely benign
693	c.3238T>C	rs754597283	Benign	Likely benign
694	c.3237A>G	rs1555588126	Benign	Likely benign
695	c.3234A>G	rs876660522	Benign	Likely benign
696	c.3231G>A	rs1567792423	Benign	Likely benign
697	c.3213A>G	rs528254652	Benign	Likely benign
698	c.3210A>T	rs1131692068	Pathogenic	Likely benign
699	c.3198A>G		Benign	Likely benign
700	c.3189C>T		Benign	Likely benign
701	c.3171T>C	rs746394738	Benign	Likely benign

702	c.3165C>T	rs1399306338	Benign	Likely benign
703	c.3153T>C	rs1057521053	Benign	Likely benign
704	c.3144T>C	rs80356837	Benign	Likely benign
705	c.3141A>G		Pathogenic	Likely benign
706	c.3129T>C	rs1555588339	Benign	Likely benign
707	c.3093T>A	rs786204265	Benign	Likely benign
708	c.3090C>T	rs1221088119	Benign	Likely benign
709	c.3084T>C		Benign	Likely benign
710	c.3075A>C	rs786201258	Benign	Likely benign
711	c.3072C>T		Benign	Likely benign
712	c.3069G>A	rs1131692091	Pathogenic	Likely benign
713	c.3066A>T	rs1555588441	Pathogenic	Likely benign
714	c.3063T>C	rs1364539273	Benign	Likely benign
715	c.3060A>G	rs781435355	Benign	Likely benign
716	c.3054C>T	rs1429701835	Benign	Likely benign
717	c.3045A>C	rs1131692079	Benign	Likely benign
718	c.3033A>G		Benign	Likely benign
719	c.3030T>G	rs876660048	Benign	Likely benign
720	c.3021A>G	rs1057521025	Pathogenic	Likely benign
721	c.3018T>C	rs1567793232	Benign	Likely benign
722	c.3012G>A	rs786201784	Pathogenic	Likely benign
723	c.3009T>C	rs786201587	Benign	Likely benign
724	c.3003A>G	rs774644946	Benign	Likely benign
725	c.2995C>T	rs80356848	Benign	Likely benign
726	c.2985G>A	rs1555588591	Pathogenic	Likely benign
727	c.2979A>G	rs772854836	Benign	Likely benign
728	c.2943A>T	rs587780799	Benign	Likely benign
729	c.2937T>C		Benign	Likely benign
730	c.2931A>C	rs273899691	Benign	Likely benign
731	c.2931A>G	rs273899691	Benign	Likely benign
732	c.2920T>C	rs763845063	Benign	Likely benign
733	c.2913T>C	rs786203804	Benign	Likely benign
734	c.2910A>G	rs431825394	Benign	Likely benign
735	c.2900C>T		Benign	Likely benign
736	c.2898T>C	rs786202249	Benign	Likely benign
737	c.2895C>T	rs1555588774	Benign	Likely benign
738	c.2892A>G	rs1060504553	Benign	Likely benign
739	c.2891G>A		Pathogenic	Likely benign
740	c.2889T>C	rs876659125	Benign	Likely benign
741	c.2883C>T	rs201190540	Benign	Likely benign
742	c.2877A>G	rs587782743	Pathogenic	Likely benign
743	c.2874C>T	rs1029207537	Benign	Likely benign
744	c.2865A>G	rs748285767	Pathogenic	Likely benign
745	c.2865A>T	rs748285767	Benign	Likely benign
746	c.2862A>C	rs559190752	Benign	Likely benign
747	c.2862A>G	rs559190752	Pathogenic	Likely benign
748	c.2860C>T	rs730881452	Benign	Likely benign
749	c.2859T>C		Benign	Likely benign
750	c.2851A>C	rs1347533994	Benign	Likely benign
751	c.2847C>T	rs1555588850	Benign	Likely benign
752	c.2829A>G	rs1131692088	Benign	Likely benign
753	c.2823T>C		Benign	Likely benign
754	c.2814A>C	rs80356851	Benign	Likely benign
755	c.2811G>A	rs876659271	Pathogenic	Likely benign

756	c.2793G>A	rs876660555	Pathogenic	Likely benign
757	c.2790T>C	rs876659151	Benign	Likely benign
758	c.2775C>T	rs786201104	Benign	Likely benign
759	c.2763G>A	rs1057522511	Pathogenic	Likely benign
760	c.2757T>C	rs755516286	Benign	Likely benign
761	c.2745T>G	rs876658748	Benign	Likely benign
762	c.2742G>A	rs961042365	Benign	Likely benign
763	c.2739T>C	rs273899688	Benign	Likely benign
764	c.2733A>G	rs1800740	Benign	Likely benign
765	c.2727T>C	rs1057520470	Benign	Likely benign
766	c.2721A>G	rs1567794510	Benign	Likely benign
767	c.2712A>G	rs1057522242	Benign	Likely benign
768	c.2706A>G	rs398122665	Benign	Likely benign
769	c.2682A>G	rs1131692093	Benign	Likely benign
770	c.2679G>A	rs587781771	Pathogenic	Likely benign
771	c.2674T>C	rs137998759	Benign	Likely benign
772	c.2670G>T	rs786201677	Benign	Likely benign
773	c.2667T>C	rs1057521536	Benign	Likely benign
774	c.2664C>T		Benign	Likely benign
775	c.2661C>G		Benign	Likely benign
776	c.2658T>G	rs1555589207	Benign	Likely benign
777	c.2634A>G	rs730881451	Pathogenic	Likely benign
778	c.2625A>G	rs754222140	Pathogenic	Likely benign
779	c.2625A>T	rs754222140	Pathogenic	Likely benign
780	c.2622T>C		Benign	Likely benign
781	c.2619A>T		Pathogenic	Likely benign
782	c.2613G>A	rs587782608	Benign	Likely benign
783	c.2604A>C	rs864622491	Benign	Likely benign
784	c.2601G>A	rs876659166	Benign	Likely benign
785	c.2595G>A		Pathogenic	Likely benign
786	c.2583C>T	rs1555589334	Pathogenic	Likely benign
787	c.2580A>C	rs556684572	Benign	Likely benign
788	c.2580A>G	rs556684572	Benign	Likely benign
789	c.2577T>C	rs1555589344	Benign	Likely benign
790	c.2574G>A	rs1555589357	Pathogenic	Likely benign
791	c.2571G>A		Pathogenic	Likely benign
792	c.2569T>C	rs779895958	Benign	Likely benign
793	c.2568T>C	rs80356832	Benign	Likely benign
794	c.2565G>A		Pathogenic	Likely benign
795	c.2556T>C	rs1555589414	Benign	Likely benign
796	c.2535A>C	rs876660248	Benign	Likely benign
797	c.2523G>C	rs773013395	Benign	Likely benign
798	c.2523G>A	rs773013395	Benign	Likely benign
799	c.2522G>A	rs80357337	Benign	Likely benign
800	c.2497T>C	rs887578121	Benign	Likely benign
801	c.2496A>T	rs767666029	Benign	Likely benign
802	c.2475C>T	rs1060504581	Benign	Likely benign
803	c.2472T>C	rs786201415	Benign	Likely benign
804	c.2466T>C	rs1064794701	Benign	Likely benign
805	c.2440C>T	rs786202054	Benign	Likely benign
806	c.2433C>T	rs1555589666	Benign	Likely benign
807	c.2421A>G	rs772960140	Pathogenic	Likely benign
808	c.2412G>A	rs55746541	Pathogenic	Likely benign
809	c.2409T>C	rs1555589731	Benign	Likely benign

810	c.2376G>A		Pathogenic	Likely benign
811	c.2374G>A	rs1555589778	Pathogenic	Likely benign
812	c.2352G>C		Benign	Likely benign
813	c.2352G>A	rs372017932	Benign	Likely benign
814	c.2340G>A	rs1555589856	Pathogenic	Likely benign
815	c.2334C>T	rs777404687	Benign	Likely benign
816	c.2322T>A	rs397508958	Pathogenic	Likely benign
817	c.2316A>C	rs876658590	Benign	Likely benign
818	c.2310A>G		Pathogenic	Likely benign
819	c.2295G>A	rs201875054	Pathogenic	Likely benign
820	c.2292A>G	rs1555589969	Pathogenic	Likely benign
821	c.2289T>C	rs1567796270	Benign	Likely benign
822	c.2284A>C	rs1555589988	Benign	Likely benign
823	c.2274G>A	rs876660823	Pathogenic	Likely benign
824	c.2247T>C	rs1289739423	Benign	Likely benign
825	c.2241C>T		Benign	Likely benign
826	c.2232T>G	rs4986846	Benign	Likely benign
827	c.2230G>A	rs786203435	Pathogenic	Likely benign
828	c.2223T>C	rs1555590129	Benign	Likely benign
829	c.2217A>G	rs200521980	Benign	Likely benign
830	c.2214T>C		Benign	Likely benign
831	c.2207A>G	rs397507196	Benign	Likely benign
832	c.2205A>G	rs1555590208	Pathogenic	Likely benign
833	c.2203C>T	rs587781781	Benign	Likely benign
834	c.2190A>G	rs1555590252	Benign	Likely benign
835	c.2187A>G		Benign	Likely benign
836	c.2178T>A	rs1555590270	Benign	Likely benign
837	c.2177T>C	rs1135401827	Benign	Likely benign
838	c.2175C>T	rs273898680	Benign	Likely benign
839	c.2157A>G		Benign	Likely benign
840	c.2154T>G	rs779227326	Benign	Likely benign
841	c.2142T>C	rs1057522371	Benign	Likely benign
842	c.2139A>G		Pathogenic	Likely benign
843	c.2139A>C	rs1131692089	Benign	Likely benign
844	c.2133G>A	rs1567796898	Pathogenic	Likely benign
845	c.2130T>G	rs273898678	Benign	Likely benign
846	c.2127T>C		Benign	Likely benign
847	c.2121T>G	rs786201649	Benign	Likely benign
848	c.2115A>G	rs1131692099	Benign	Likely benign
849	c.2115A>T	rs1131692099	Benign	Likely benign
850	c.2109A>C	rs4986844	Benign	Likely benign
851	c.2103G>A	rs273898677	Benign	Likely benign
852	c.2094A>T	rs1131692098	Pathogenic	Likely benign
853	c.2079C>T	rs80356835	Benign	Likely benign
854	c.2076T>C	rs587782595	Benign	Likely benign
855	c.2064A>T		Pathogenic	Likely benign
856	c.2064A>G	rs1131692077	Benign	Likely benign
857	c.2055T>C	rs1555590600	Benign	Likely benign
858	c.2049G>A	rs778215185	Pathogenic	Likely benign
859	c.2037G>A	rs572835027	Pathogenic	Likely benign
860	c.2028T>G		Benign	Likely benign
861	c.2022T>G	rs771519405	Benign	Likely benign
862	c.2016A>G	rs776542749	Benign	Likely benign
863	c.2004C>T	rs1057520832	Benign	Likely benign

864	c.2004C>A	rs1057520832	Pathogenic	Likely benign
865	c.2001A>G	rs878854937	Benign	Likely benign
866	c.1998A>G	rs864622452	Pathogenic	Likely benign
867	c.1996C>T		Benign	Likely benign
868	c.1989C>T		Benign	Likely benign
869	c.1986C>T	rs1555590719	Benign	Likely benign
870	c.1983G>A	rs869320788	Benign	Likely benign
871	c.1968C>T	rs1131692075	Benign	Likely benign
872	c.1966A>G	rs786203455	Benign	Likely benign
873	c.1962G>A		Pathogenic	Likely benign
874	c.1959A>G	rs767530204	Benign	Likely benign
875	c.1950A>T	rs1060504579	Benign	Likely benign
876	c.1947G>A	rs755706172	Pathogenic	Likely benign
877	c.1944A>G	rs876660781	Benign	Likely benign
878	c.1926T>C	rs786203720	Benign	Likely benign
879	c.1917G>A	rs786202103	Pathogenic	Likely benign
880	c.1912G>A	rs80357005	Pathogenic	Likely benign
881	c.1911T>C	rs62625305	Benign	Likely benign
882	c.1905T>C	rs369373293	Benign	Likely benign
883	c.1893A>C	rs80356834	Benign	Likely benign
884	c.1893A>G	rs80356834	Pathogenic	Likely benign
885	c.1884T>C		Benign	Likely benign
886	c.1881C>T	rs80356838	Benign	Likely benign
887	c.1878A>G	rs8176154	Pathogenic	Likely benign
888	c.1875A>G	rs786201429	Pathogenic	Likely benign
889	c.1873C>T	rs769044421	Benign	Likely benign
890	c.1866G>A	rs1800064	Benign	Likely benign
891	c.1866G>T	rs1800064	Benign	Likely benign
892	c.1863T>C	rs786201460	Benign	Likely benign
893	c.1854G>A	rs1060504585	Benign	Likely benign
894	c.1851C>G	rs1555591097	Benign	Likely benign
895	c.1842G>A	rs760109939	Benign	Likely benign
896	c.1839G>A	rs759157605	Benign	Likely benign
897	c.1833G>A	rs786201548	Pathogenic	Likely benign
898	c.1830G>A	rs587780796	Benign	Likely benign
899	c.1827T>C	rs757657445	Benign	Likely benign
900	c.1809A>C		Benign	Likely benign
901	c.1803C>T	rs1057523373	Benign	Likely benign
902	c.1797T>C	rs756211343	Benign	Likely benign
903	c.1794A>G	rs876659644	Pathogenic	Likely benign
904	c.1792T>C	rs1060504554	Benign	Likely benign
905	c.1788C>T	rs779253414	Benign	Likely benign
906	c.1779T>C	rs1060504563	Benign	Likely benign
907	c.1776C>T	rs876658911	Pathogenic	Likely benign
908	c.1773A>C	rs1555591259	Benign	Likely benign
909	c.1764C>T		Benign	Likely benign
910	c.1761A>C	rs1057523389	Benign	Likely benign
911	c.1758T>C	rs1567798401	Benign	Likely benign
912	c.1752T>A		Benign	Likely benign
913	c.1752T>C		Benign	Likely benign
914	c.1749A>G	rs876659580	Benign	Likely benign
915	c.1746G>A	rs776115545	Benign	Likely benign
916	c.1740C>T	rs1057520244	Pathogenic	Likely benign
917	c.1737T>G	rs1567798463	Benign	Likely benign

918	c.1731A>G	rs28897678	Benign	Likely benign
919	c.1728A>G	rs786201232	Benign	Likely benign
920	c.1722C>T	rs530914551	Benign	Likely benign
921	c.1710A>G	rs876659901	Benign	Likely benign
922	c.1707C>T	rs876659110	Benign	Likely benign
923	c.1704T>C	rs587780795	Benign	Likely benign
924	c.1704T>G	rs587780795	Benign	Likely benign
925	c.1650T>C	rs777595821	Benign	Likely benign
926	c.1635G>A	rs770842236	Pathogenic	Likely benign
927	c.1632A>G	rs876658401	Benign	Likely benign
928	c.1617G>A	rs372002119	Benign	Likely benign
929	c.1608T>G	rs1555591482	Benign	Likely benign
930	c.1599T>C		Benign	Likely benign
931	c.1596A>T	rs1555591508	Benign	Likely benign
932	c.1587T>C		Benign	Likely benign
933	c.1578A>G	rs762642319	Benign	Likely benign
934	c.1569G>A	rs766934857	Pathogenic	Likely benign
935	c.1567T>C	rs754398271	Benign	Likely benign
936	c.1563A>G	rs754970915	Pathogenic	Likely benign
937	c.1536T>C		Benign	Likely benign
938	c.1533C>G		Benign	Likely benign
939	c.1533C>A	rs1280391272	Benign	Likely benign
940	c.1530A>T	rs1555591599	Pathogenic	Likely benign
941	c.1527A>G	rs1131692082	Benign	Likely benign
942	c.1524T>C	rs200616937	Benign	Likely benign
943	c.1524T>G	rs200616937	Benign	Likely benign
944	c.1522C>G	rs1555591622	Benign	Likely benign
945	c.1506A>G	rs786203671	Pathogenic	Likely benign
946	c.1500T>C		Benign	Likely benign
947	c.1497A>T		Pathogenic	Likely benign
948	c.1491C>G	rs786202374	Benign	Likely benign
949	c.1487G>T	rs28897677	Benign	Likely benign
950	c.1470A>G	rs775032066	Benign	Likely benign
951	c.1467G>A	rs794726997	Pathogenic	Likely benign
952	c.1464T>C	rs1555591722	Benign	Likely benign
953	c.1458T>A	rs80357400	Benign	Likely benign
954	c.1446T>C		Benign	Likely benign
955	c.1434T>G	rs876658280	Benign	Likely benign
956	c.1428T>C	rs1060504572	Benign	Likely benign
957	c.1427A>G	rs55720177	Benign	Likely benign
958	c.1421T>C		Benign	Likely benign
959	c.1419C>T	rs777228325	Benign	Likely benign
960	c.1416C>T	rs752808917	Benign	Likely benign
961	c.1404G>A	rs1555591782	Pathogenic	Likely benign
962	c.1401G>A	rs786201323	Pathogenic	Likely benign
963	c.1398G>A	rs1060504571	Benign	Likely benign
964	c.1392C>G	rs533802049	Benign	Likely benign
965	c.1390A>G	rs869320787	Benign	Likely benign
966	c.1386G>A	rs876659749	Pathogenic	Likely benign
967	c.1374C>T	rs879253999	Benign	Likely benign
968	c.1362T>C	rs1555591854	Benign	Likely benign
969	c.1353A>G	rs1178888270	Pathogenic	Likely benign
970	c.1344C>T	rs1395644015	Benign	Likely benign
971	c.1336A>C		Benign	Likely benign

972	c.1335A>G	rs1131692094	Benign	Likely benign
973	c.1329A>G	rs771892131	Benign	Likely benign
974	c.1317T>C	rs1555591949	Benign	Likely benign
975	c.1308T>C	rs770279083	Benign	Likely benign
976	c.1302T>C		Benign	Likely benign
977	c.1294C>T	rs864622454	Benign	Likely benign
978	c.1284A>G		Benign	Likely benign
979	c.1278A>C	rs1442003131	Benign	Likely benign
980	c.1278A>G	rs1442003131	Pathogenic	Likely benign
981	c.1275T>A	rs786201160	Benign	Likely benign
982	c.1260T>C		Benign	Likely benign
983	c.1257A>G	rs751690840	Pathogenic	Likely benign
984	c.1257A>T	rs751690840	Pathogenic	Likely benign
985	c.1254G>A	rs786201948	Pathogenic	Likely benign
986	c.1251T>C	rs80357197	Benign	Likely benign
987	c.1248A>G	rs1057522369	Pathogenic	Likely benign
988	c.1242C>T	rs372400428	Benign	Likely benign
989	c.1237T>G	rs574008372	Benign	Likely benign
990	c.1237T>C	rs574008372	Benign	Likely benign
991	c.1236A>G	rs1555592094	Pathogenic	Likely benign
992	c.1233T>G	rs80357024	Benign	Likely benign
993	c.1227A>G	rs149349675	Pathogenic	Likely benign
994	c.1215A>G	rs786201517	Pathogenic	Likely benign
995	c.1212A>G	rs1057521863	Benign	Likely benign
996	c.1209T>C	rs369363742	Benign	Likely benign
997	c.1203G>A	rs1567800556	Benign	Likely benign
998	c.1197T>C	rs1555592250	Benign	Likely benign
999	c.1173A>G	rs1131692097	Benign	Likely benign
1000	c.1161C>T		Benign	Likely benign
1001	c.1152G>A	rs1057523704	Pathogenic	Likely benign
1002	c.1149T>C	rs979531844	Benign	Likely benign
1003	c.1137T>G	rs56128296	Pathogenic	Likely benign
1004	c.1128T>C	rs1555592423	Benign	Likely benign
1005	c.1125A>G	rs1060504578	Pathogenic	Likely benign
1006	c.1123C>T	rs1131692084	Benign	Likely benign
1007	c.1113T>C	rs876658619	Benign	Likely benign
1008	c.1098T>C	rs876658148	Benign	Likely benign
1009	c.1092T>C	rs1555592521	Benign	Likely benign
1010	c.1072C>T	rs377310179	Benign	Likely benign
1011	c.1071A>G	rs786202159	Benign	Likely benign
1012	c.1065G>A	rs41286292	Pathogenic	Likely benign
1013	c.1041G>T		Benign	Likely benign
1014	c.1041G>A	rs1555592609	Pathogenic	Likely benign
1015	c.1039C>T	rs1378561919	Benign	Likely benign
1016	c.1038C>T	rs1555592618	Benign	Likely benign
1017	c.1017G>A	rs863224416	Pathogenic	Likely benign
1018	c.1014A>G	rs876660793	Benign	Likely benign
1019	c.1002C>T	rs1555592693	Benign	Likely benign
1020	c.1001C>A	rs41286290	Pathogenic	Likely benign
1021	c.999T>A		Benign	Likely benign
1022	c.996G>T	rs80356836	Pathogenic	Likely benign
1023	c.990T>C	rs978690648	Benign	Likely benign
1024	c.987T>C	rs774849810	Benign	Likely benign
1025	c.981A>C	rs1800063	Benign	Likely benign

1026	c.975G>A	rs786201624	Benign	Likely benign
1027	c.969A>T	rs45586033	Pathogenic	Likely benign
1028	c.957C>T	rs1057524333	Benign	Likely benign
1029	c.956A>G	rs397507258	Benign	Likely benign
1030	c.951A>G	rs759419385	Benign	Likely benign
1031	c.945G>A	rs1555592789	Pathogenic	Likely benign
1032	c.937T>C	rs1131692072	Benign	Likely benign
1033	c.936C>T		Benign	Likely benign
1034	c.927A>G	rs757936216	Benign	Likely benign
1035	c.918T>C	rs1555592835	Benign	Likely benign
1036	c.888A>G	rs1555592914	Benign	Likely benign
1037	c.885C>T	rs1060504557	Benign	Likely benign
1038	c.879T>G	rs139433219	Benign	Likely benign
1039	c.876C>T	rs771001707	Benign	Likely benign
1040	c.843C>T		Benign	Likely benign
1041	c.837T>C	rs775477245	Benign	Likely benign
1042	c.834T>A		Pathogenic	Likely benign
1043	c.834T>G	rs762956862	Benign	Likely benign
1044	c.828A>G	rs186274774	Benign	Likely benign
1045	c.825C>T	rs397509328	Benign	Likely benign
1046	c.819A>G	rs779225364	Benign	Likely benign
1047	c.810T>C	rs778359104	Benign	Likely benign
1048	c.804C>T	rs771076131	Benign	Likely benign
1049	c.789T>C	rs397509320	Benign	Likely benign
1050	c.786G>A	rs397509317	Pathogenic	Likely benign
1051	c.768G>A	rs746067447	Benign	Likely benign
1052	c.766A>C	rs587781833	Benign	Likely benign
1053	c.765G>A	rs62625299	Pathogenic	Likely benign
1054	c.762T>C	rs1555593173	Benign	Likely benign
1055	c.759A>T	rs1131692069	Pathogenic	Likely benign
1056	c.756T>C	rs786201338	Benign	Likely benign
1057	c.750G>A	rs762867923	Pathogenic	Likely benign
1058	c.744C>T	rs886037791	Benign	Likely benign
1059	c.744C>G	rs886037791	Benign	Likely benign
1060	c.741C>T		Benign	Likely benign
1061	c.739A>G	rs767720128	Benign	Likely benign
1062	c.738G>A	rs768416164	Pathogenic	Likely benign
1063	c.736T>C	rs28897675	Benign	Likely benign
1064	c.705T>C	rs1165432483	Benign	Likely benign
1065	c.699A>G	rs786202162	Pathogenic	Likely benign
1066	c.696T>C	rs1057523563	Benign	Likely benign
1067	c.693G>T	rs62625298	Benign	Likely benign
1068	c.693G>A	rs62625298	Benign	Likely benign
1069	c.675T>G	rs1555593332	Benign	Likely benign
1070	c.671-11T>C		Benign	Likely benign
1071	c.670+18C>A		Benign	Likely benign
1072	c.670+8C>T	rs80358050	Pathogenic	Likely benign
1073	c.669G>A	rs1131692066	Pathogenic	Likely benign
1074	c.654G>A	rs1555593548	Pathogenic	Likely benign
1075	c.652T>C	rs765950064	Benign	Likely benign
1076	c.639G>A	rs1131692078	Benign	Likely benign
1077	c.621C>T	rs1555593586	Benign	Likely benign
1078	c.618A>G		Benign	Likely benign
1079	c.615A>G		Pathogenic	Likely benign

1080	c.612G>A		Pathogenic	Likely benign
1081	c.603T>C	rs786201512	Benign	Likely benign
1082	c.600A>G	rs1555593617	Pathogenic	Likely benign
1083	c.588T>C	rs1131692071	Benign	Likely benign
1084	c.579G>A	rs759197544	Benign	Likely benign
1085	c.576T>C	rs1555594046	Benign	Likely benign
1086	c.570C>A	rs201536070	Benign	Likely benign
1087	c.570C>T	rs201536070	Benign	Likely benign
1088	c.567T>C	rs80356845	Benign	Likely benign
1089	c.564A>G	rs768065826	Benign	Likely benign
1090	c.549A>C	rs1555594077	Benign	Likely benign
1091	c.547+16T>G		Benign	Likely benign
1092	c.546G>A		Benign	Likely benign
1093	c.543A>G	rs397507250	Benign	Likely benign
1094	c.528G>A	rs34545365	Benign	Likely benign
1095	c.525G>A	rs1555594837	Pathogenic	Likely benign
1096	c.522A>G	rs765432756	Benign	Likely benign
1097	c.519T>A	rs876659179	Benign	Likely benign
1098	c.516A>G	rs752940034	Benign	Likely benign
1099	c.511A>G	rs777515082	Benign	Likely benign
1100	c.508C>A		Benign	Likely benign
1101	c.507G>A	rs759882045	Benign	Likely benign
1102	c.495G>A	rs745321499	Pathogenic	Likely benign
1103	c.486G>T	rs769213707	Pathogenic	Likely benign
1104	c.483T>C	rs1060504575	Benign	Likely benign
1105	c.477T>C	rs779704727	Benign	Likely benign
1106	c.468C>G	rs748923729	Benign	Likely benign
1107	c.459T>C	rs1555594962	Benign	Likely benign
1108	c.439T>C	rs794727800	Benign	Likely benign
1109	c.438C>T	rs1555596287	Benign	Likely benign
1110	c.426C>G		Benign	Likely benign
1111	c.426C>T	rs542687218	Benign	Likely benign
1112	c.420T>C	rs730881448	Benign	Likely benign
1113	c.414A>G		Pathogenic	Likely benign
1114	c.411T>C	rs1258746087	Benign	Likely benign
1115	c.399T>C	rs1555596341	Benign	Likely benign
1116	c.397C>A	rs80357457	Benign	Likely benign
1117	c.390C>T	rs80356888	Benign	Likely benign
1118	c.378A>G	rs786201256	Benign	Likely benign
1119	c.375C>T	rs1311672121	Benign	Likely benign
1120	c.372C>A	rs273900715	Pathogenic	Likely benign
1121	c.369T>A	rs774583925	Benign	Likely benign
1122	c.366T>G	rs190900046	Benign	Likely benign
1123	c.363A>G	rs1060504552	Benign	Likely benign
1124	c.354A>G	rs1320340280	Pathogenic	Likely benign
1125	c.342T>C		Benign	Likely benign
1126	c.339C>T	rs587779367	Benign	Likely benign
1127	c.330G>A	rs878854947	Benign	Likely benign
1128	c.309C>T	rs876659814	Benign	Likely benign
1129	c.303T>C	rs80356936	Benign	Likely benign
1130	c.301+13T>A		Benign	Likely benign
1131	c.300G>A	rs1555596638	Benign	Likely benign
1132	c.291A>C		Benign	Likely benign
1133	c.291A>G	rs1131692085	Pathogenic	Likely benign

1134	c.288C>T	rs146085503	Benign	Likely benign
1135	c.282G>T		Benign	Likely benign
1136	c.270T>C	rs1555596670	Benign	Likely benign
1137	c.267C>G	rs80356963	Pathogenic	Likely benign
1138	c.261G>A	rs757971617	Pathogenic	Likely benign
1139	c.259T>C	rs80357091	Benign	Likely benign
1140	c.258A>T	rs777491912	Benign	Likely benign
1141	c.258A>G	rs777491912	Pathogenic	Likely benign
1142	c.255G>A	rs756499058	Benign	Likely benign
1143	c.249T>C	rs780485347	Benign	Likely benign
1144	c.243A>G	rs863224418	Benign	Likely benign
1145	c.231G>A	rs80356847	Benign	Likely benign
1146	c.231G>C	rs80356847	Benign	Likely benign
1147	c.231G>T	rs80356847	Benign	Likely benign
1148	c.222A>G	rs730881465	Benign	Likely benign
1149	c.219A>G	rs876659123	Pathogenic	Likely benign
1150	c.217C>T	rs786201203	Benign	Likely benign
1151	c.216C>T		Benign	Likely benign
1152	c.212+14T>G		Benign	Likely benign
1153	c.212+10T>G	rs80358174	Benign	Likely benign
1154	c.183T>C	rs895070717	Benign	Likely benign
1155	c.171G>T	rs1057523606	Pathogenic	Likely benign
1156	c.159C>T	rs1060504588	Benign	Likely benign
1157	c.153T>G	rs1131692081	Pathogenic	Likely benign
1158	c.150A>G	rs1555597289	Benign	Likely benign
1159	c.147G>A	rs1555597297	Pathogenic	Likely benign
1160	c.141C>T	rs398122635	Benign	Likely benign
1161	c.134+9G>A		Pathogenic	Likely benign
1162	c.123C>T	rs786202211	Benign	Likely benign
1163	c.120C>T	rs1060504580	Benign	Likely benign
1164	c.111A>C		Benign	Likely benign
1165	c.111A>G	rs1293537445	Benign	Likely benign
1166	c.102T>G	rs1555599260	Benign	Likely benign
1167	c.87G>A		Benign	Likely benign
1168	c.84G>A	rs1555599278	Benign	Likely benign
1169	c.82C>T		Benign	Likely benign
1170	c.81-4C>T		Benign	Likely benign
1171	c.81-14C>A		Benign	Likely benign
1172	c.81-16C>T		Benign	Likely benign
1173	c.78C>T	rs1555600862	Benign	Likely benign
1174	c.75C>T	rs80356839	Benign	Likely benign
1175	c.69G>A	rs766004110	Benign	Likely benign
1176	c.66A>G	rs786202533	Pathogenic	Likely benign
1177	c.64T>C		Benign	Likely benign
1178	c.63C>T	rs1555600921	Benign	Likely benign
1179	c.61A>G	rs80357406	Benign	Likely benign
1180	c.60A>G	rs202168814	Benign	Likely benign
1181	c.48T>C		Benign	Likely benign
1182	c.42C>T	rs80356827	Benign	Likely benign
1183	c.42C>A	rs80356827	Benign	Likely benign
1184	c.36A>G	rs763230080	Benign	Likely benign
1185	c.33A>G	rs1555601010	Pathogenic	Likely benign
1186	c.15T>C		Benign	Likely benign
1187	c.9A>G		Pathogenic	Likely benign

1188	c.6T>C	rs754763517	Benign	Likely benign
1189	c.-17C>T		Benign	Likely benign
1190	c.-19-10T>C	rs201866997	Benign	Likely benign
1191	c.-20+57G>A	rs556032779	Benign	Likely benign
1192	c.-20+55G>A	rs772215665	Benign	Likely benign
1193	c.-20+28G>A		Benign	Likely benign
1194	c.-20+13C>G		Benign	Likely benign
1195	c.-20+13C>T	rs770368390	Benign	Likely benign
1196	c.-20+11C>T	rs273898672	Benign	Likely benign
1197	c.-25G>A	rs1057520890	Benign	Likely benign
1198	c.-26G>T	rs1057522752	Benign	Likely benign
1199	c.-29C>G	rs774839252	Benign	Likely benign
1200	c.-37C>T	rs1057524754	Benign	Likely benign
1201	c.-38C>T	rs1555601360	Benign	Likely benign
1202	c.-45G>C	rs1057521869	Benign	Likely benign
1203	c.-49G>A	rs1057523081	Benign	Likely benign
1204	c.-53C>T		Benign	Likely benign
1205	c.5558A>G	rs80357258	Pathogenic	Likely pathogenic
1206	c.5527G>C	rs80357019	Pathogenic	Likely pathogenic
1207	c.5521A>C	rs80357299	Benign	Likely pathogenic
1208	c.5513T>G	rs80357107	Benign	Likely pathogenic
1209	c.5509T>G	rs80356959	Benign	Likely pathogenic
1210	c.5497G>A	rs80357268	Pathogenic	Likely Pathogenic
1211	c.5434C>G	rs1800751	Benign	Likely Pathogenic
1212	c.5426T>A		Benign	Likely pathogenic
1213	c.5425G>T	rs28897698	Benign	Likely pathogenic
1214	c.5408G>C	rs80357149	Benign	Likely Pathogenic
1215	c.5407-1G>A	rs80358029	Pathogenic	Likely Pathogenic
1216	c.5363G>C	rs80357069	Benign	Likely pathogenic
1217	c.5362G>T	rs397509271	Benign	Likely pathogenic
1218	c.5339T>C	rs80357474	Benign	Likely Pathogenic
1219	c.5307T>G	rs397509258	Benign	Likely pathogenic
1220	c.5278-2A>G	rs397509253	Pathogenic	Likely Pathogenic
1221	c.5277G>T	rs80356854	Benign	Likely pathogenic
1222	c.5254G>C	rs80357074	Benign	Likely pathogenic
1223	c.5252G>C	rs80357442	Benign	Likely pathogenic
1224	c.5243G>A	rs397509243	Pathogenic	Likely pathogenic
1225	c.5213G>A	rs80357450	Pathogenic	Likely Pathogenic
1226	c.5207T>G	rs45553935	Benign	Likely Pathogenic
1227	c.5194-2A>G	rs80358069	Pathogenic	Likely Pathogenic
1228	c.5193+1G>T	rs80358004	Pathogenic	Likely Pathogenic
1229	c.5165C>T	rs80357104	Benign	Likely Pathogenic
1230	c.5164T>C	rs483353100	Benign	Likely pathogenic
1231	c.5154G>T	rs80357239	Benign	Likely pathogenic
1232	c.5153G>C	rs41293461	Pathogenic	Likely pathogenic
1233	c.5141T>G	rs80357243	Benign	Likely pathogenic
1234	c.5116G>A	rs886040864	Pathogenic	Likely Pathogenic
1235	c.5114T>C	rs397507242	Benign	Likely Pathogenic
1236	c.5098A>G	rs397509227	Benign	Likely pathogenic
1237	c.5075-1G>A	rs1800747	Pathogenic	Likely Pathogenic
1238	c.5075-3C>G	rs398122690	Benign	Likely pathogenic
1239	c.5074+3A>G	rs80358181	Pathogenic	Likely Pathogenic
1240	c.5074G>A	rs80187739	Pathogenic	Likely Pathogenic
1241	c.5072C>T	rs80357034	Benign	Likely pathogenic

1242	c.5072C>A	rs80357034	Benign	Likely pathogenic
1243	c.5066T>G	rs80357061	Benign	Likely Pathogenic
1244	c.5057A>G	rs730882166	Pathogenic	Likely Pathogenic
1245	c.4986+6T>C	rs80358086	Benign	Likely Pathogenic
1246	c.4986+4A>C	rs80358087	Benign	Likely Pathogenic
1247	c.4986+3G>C	rs80358023	Benign	Likely Pathogenic
1248	c.4964C>T	rs80357390	Benign	Likely Pathogenic
1249	c.4888G>T	rs1173155015	Pathogenic	Likely pathogenic
1250	c.4868C>G	rs80356862	Benign	Likely Pathogenic
1251	c.4675G>C	rs80356988	Benign	Likely Pathogenic
1252	c.4485-1G>A	rs80358189	Pathogenic	Likely Pathogenic
1253	c.4485-2A>G	rs80358054	Pathogenic	Likely Pathogenic
1254	c.4484G>A	rs80357389	Pathogenic	Likely Pathogenic
1255	c.4358-2786G>A	rs374435098	Pathogenic	Likely pathogenic
1256	c.4232T>C	rs273900729	Benign	Likely pathogenic
1257	c.4224G>C		Benign	Likely pathogenic
1258	c.4185G>C	rs80356857	Benign	Likely pathogenic
1259	c.4093T>G	rs1567788936	Benign	Likely pathogenic
1260	c.2351C>A	rs55914168	Benign	Likely pathogenic
1261	c.869T>A	rs730881468	Benign	Likely Pathogenic
1262	c.688G>T	rs1555593310	Pathogenic	Likely Pathogenic
1263	c.671-1G>T	rs80358020	Benign	Likely Pathogenic
1264	c.614T>G	rs1555593598	Benign	Likely pathogenic
1265	c.441+2T>A	rs397509173	Pathogenic	Likely Pathogenic
1266	c.287A>G	rs864622444	Benign	Likely pathogenic
1267	c.212+3A>T		Benign	Likely pathogenic
1268	c.212G>T	rs80356913	Benign	Likely Pathogenic
1269	c.192T>G	rs587781632	Benign	Likely Pathogenic
1270	c.182G>A	rs80357093	Pathogenic	Likely Pathogenic
1271	c.181T>C	rs28897672	Benign	Likely Pathogenic
1272	c.140G>T	rs80357150	Benign	Likely Pathogenic
1273	c.139T>G	rs80357370	Benign	Likely pathogenic
1274	c.134+5G>T	rs80358038	Benign	Likely pathogenic
1275	c.134+5G>A	rs80358038	Benign	Likely pathogenic
1276	c.134+5G>C	rs80358038	Benign	Likely pathogenic
1277	c.132C>G		Benign	Likely pathogenic
1278	c.116G>T	rs80357498	Benign	Likely Pathogenic
1279	c.115T>G	rs80357164	Benign	Likely Pathogenic
1280	c.115T>A	rs80357164	Benign	Likely Pathogenic
1281	c.110C>G	rs80356880	Benign	Likely pathogenic
1282	c.72T>G		Benign	Likely pathogenic
1283	c.70T>C	rs80357410	Benign	Likely Pathogenic
1284	c.[-1A>G;3G>A]	rs80357475|rs58	Pathogenic	Likely pathogenic
1285	c.3G>A	rs80357475	Pathogenic	Likely Pathogenic
1286	c.2T>C	rs80357111	Benign	Likely Pathogenic
1287	c.5565A>T		Benign	not provided
1288	c.5565A>G		Pathogenic	not provided
1289	c.5564T>G		Benign	not provided
1290	c.5564T>A		Benign	not provided
1291	c.5563A>T		Benign	not provided
1292	c.5563A>G		Benign	not provided
1293	c.5563A>C		Benign	not provided
1294	c.5562G>T		Pathogenic	not provided
1295	c.5562G>C		Benign	not provided

1296	c.5561T>G	Benign	not provided
1297	c.5561T>A	Benign	not provided
1298	c.5560C>T	Benign	not provided
1299	c.5560C>G	Benign	not provided
1300	c.5560C>A	Pathogenic	not provided
1301	c.5559C>T	Benign	not provided
1302	c.5558A>T	Benign	not provided
1303	c.5558A>C	Benign	not provided
1304	c.5557T>G	Benign	not provided
1305	c.5557T>C	Benign	not provided
1306	c.5557T>A	Benign	not provided
1307	c.5556C>A	Pathogenic	not provided
1308	c.5556C>T	Benign	not provided
1309	c.5555C>G	Benign	not provided
1310	c.5555C>A	Benign	not provided
1311	c.5554A>T	Benign	not provided
1312	c.5554A>C	Benign	not provided
1313	c.5553C>G	Benign	not provided
1314	c.5552A>T	Benign	not provided
1315	c.5552A>G	Benign	not provided
1316	c.5552A>C	Benign	not provided
1317	c.5551G>T	Pathogenic	not provided
1318	c.5551G>C	Benign	not provided
1319	c.5550G>T	Benign	not provided
1320	c.5549T>G	Benign	not provided
1321	c.5549T>C	Benign	not provided
1322	c.5549T>A	Benign	not provided
1323	c.5548C>T	Benign	not provided
1324	c.5548C>G	Benign	not provided
1325	c.5548C>A	Benign	not provided
1326	c.5547G>T	Benign	not provided
1327	c.5547G>C	Pathogenic	not provided
1328	c.5546A>T	Pathogenic	not provided
1329	c.5546A>G	Pathogenic	not provided
1330	c.5546A>C	Benign	not provided
1331	c.5545G>T	Pathogenic	not provided
1332	c.5545G>C	Benign	not provided
1333	c.5545G>A	Pathogenic	not provided
1334	c.5544G>T	Pathogenic	not provided
1335	c.5544G>C	Benign	not provided
1336	c.5544G>A	Pathogenic	not provided
1337	c.5543A>T	Pathogenic	not provided
1338	c.5543A>G	Pathogenic	not provided
1339	c.5543A>C	Benign	not provided
1340	c.5542C>G	Benign	not provided
1341	c.5542C>A	Pathogenic	not provided
1342	c.5541C>G	Benign	not provided
1343	c.5540G>C	Benign	not provided
1344	c.5540G>A	Pathogenic	not provided
1345	c.5539T>G	Benign	not provided
1346	c.5539T>C	Benign	not provided
1347	c.5539T>A	Benign	not provided
1348	c.5538G>T	Benign	not provided
1349	c.5537A>T	Pathogenic	not provided

1350	c.5537A>G	Pathogenic	not provided
1351	c.5537A>C	Benign	not provided
1352	c.5536C>G	Pathogenic	not provided
1353	c.5535C>T	Benign	not provided
1354	c.5534A>T	Benign	not provided
1355	c.5534A>C	Benign	not provided
1356	c.5533T>G	Benign	not provided
1357	c.5533T>C	Benign	not provided
1358	c.5533T>A	Benign	not provided
1359	c.5532C>G	Benign	not provided
1360	c.5532C>A	Pathogenic	not provided
1361	c.5531T>A	Benign	not provided
1362	c.5530C>T	Benign	not provided
1363	c.5530C>G	Benign	not provided
1364	c.5529A>T	Benign	not provided
1365	c.5528C>G	Benign	not provided
1366	c.5527G>T	Benign	not provided
1367	c.5527G>A	Pathogenic	not provided
1368	c.5526A>T	Pathogenic	not provided
1369	c.5526A>C	Benign	not provided
1370	c.5525T>G	Benign	not provided
1371	c.5525T>C	Benign	not provided
1372	c.5524G>C	Benign	not provided
1373	c.5524G>A	Pathogenic	not provided
1374	c.5524G>T	Benign	not provided
1375	c.5523T>G	Benign	not provided
1376	c.5523T>A	Benign	not provided
1377	c.5522G>T	Benign	not provided
1378	c.5521A>T	Pathogenic	not provided
1379	c.5520C>T	Benign	not provided
1380	c.5520C>G	Benign	not provided
1381	c.5520C>A	Pathogenic	not provided
1382	c.5519A>T	Benign	not provided
1383	c.5519A>G	Benign	not provided
1384	c.5518G>C	Benign	not provided
1385	c.5517G>T	Benign	not provided
1386	c.5517G>C	Pathogenic	not provided
1387	c.5516T>A	Pathogenic	not provided
1388	c.5515T>G	Benign	not provided
1389	c.5515T>C	Benign	not provided
1390	c.5514G>C	Benign	not provided
1391	c.5514G>A	Pathogenic	not provided
1392	c.5513T>C	Benign	not provided
1393	c.5512G>C	Benign	not provided
1394	c.5510G>T	Benign	not provided
1395	c.5510G>C	Pathogenic	not provided
1396	c.5508G>T	Benign	not provided
1397	c.5508G>C	Benign	not provided
1398	c.5508G>A	Pathogenic	not provided
1399	c.5507A>T	Pathogenic	not provided
1400	c.5507A>G	Pathogenic	not provided
1401	c.5507A>C	Benign	not provided
1402	c.5506G>C	Benign	not provided
1403	c.5505A>T	Pathogenic	not provided

1404	c.5505A>G	Pathogenic	not provided
1405	c.5505A>C	Benign	not provided
1406	c.5504G>T	Benign	not provided
1407	c.5503C>G	Benign	not provided
1408	c.5503C>A	Benign	not provided
1409	c.5502C>G	Pathogenic	not provided
1410	c.5502C>A	Pathogenic	not provided
1411	c.5501C>G	Benign	not provided
1412	c.5500A>T	Benign	not provided
1413	c.5500A>G	Benign	not provided
1414	c.5500A>C	Benign	not provided
1415	c.5499G>T	Pathogenic	not provided
1416	c.5499G>C	Benign	not provided
1417	c.5499G>A	Pathogenic	not provided
1418	c.5498T>A	Benign	not provided
1419	c.5497G>C	Benign	not provided
1420	c.5495T>G	Benign	not provided
1421	c.5495T>C	Benign	not provided
1422	c.5494G>C	Benign	not provided
1423	c.5493T>G	Benign	not provided
1424	c.5493T>C	Benign	not provided
1425	c.5493T>A	Benign	not provided
1426	c.5492C>T	Benign	not provided
1427	c.5492C>A	Pathogenic	not provided
1428	c.5491C>T	Benign	not provided
1429	c.5491C>G	Benign	not provided
1430	c.5491C>A	Benign	not provided
1431	c.5490A>T	Benign	not provided
1432	c.5490A>G	Benign	not provided
1433	c.5490A>C	Benign	not provided
1434	c.5489C>T	Benign	not provided
1435	c.5489C>G	Benign	not provided
1436	c.5489C>A	Benign	not provided
1437	c.5488G>C	Benign	not provided
1438	c.5488G>T	Benign	not provided
1439	c.5487G>T	Benign	not provided
1440	c.5487G>C	Benign	not provided
1441	c.5487G>A	Pathogenic	not provided
1442	c.5486A>T	Pathogenic	not provided
1443	c.5486A>G	Pathogenic	not provided
1444	c.5486A>C	Benign	not provided
1445	c.5485G>T	Pathogenic	not provided
1446	c.5485G>C	Benign	not provided
1447	c.5484T>G	Benign	not provided
1448	c.5484T>C	Benign	not provided
1449	c.5484T>A	Benign	not provided
1450	c.5483G>T	Benign	not provided
1451	c.5483G>C	Pathogenic	not provided
1452	c.5483G>A	Pathogenic	not provided
1453	c.5482T>C	Benign	not provided
1454	c.5482T>A	Benign	not provided
1455	c.5481G>T	Benign	not provided
1456	c.5481G>C	Benign	not provided
1457	c.5480T>G	Benign	not provided

1458	c.5480T>C	Benign	not provided
1459	c.5480T>A	Benign	not provided
1460	c.5479A>T	Benign	not provided
1461	c.5479A>C	Benign	not provided
1462	c.5478G>C	Benign	not provided
1463	c.5477A>G	Pathogenic	not provided
1464	c.5477A>C	Benign	not provided
1465	c.5476C>T	Benign	not provided
1466	c.5476C>G	Benign	not provided
1467	c.5475G>C	Benign	not provided
1468	c.5474G>T	Benign	not provided
1469	c.5474G>C	Pathogenic	not provided
1470	c.5474G>A	Pathogenic	not provided
1471	c.5473G>T	Benign	not provided
1472	c.5473G>C	Pathogenic	not provided
1473	c.5472T>G	Benign	not provided
1474	c.5472T>C	Benign	not provided
1475	c.5472T>A	Pathogenic	not provided
1476	c.5471T>G	Benign	not provided
1477	c.5471T>C	Benign	not provided
1478	c.5471T>A	Benign	not provided
1479	c.5470A>T	Benign	not provided
1480	c.5470A>C	Benign	not provided
1481	c.5469A>T	Pathogenic	not provided
1482	c.5469A>G	Benign	not provided
1483	c.5468C>T	Pathogenic	not provided
1484	c.5468C>G	Benign	not provided
1485	c.5468C>A	Benign	not provided
1486	c.5468-1G>T	Benign	not provided
1487	c.5468-1G>C	Benign	not provided
1488	c.5468-2A>C	Benign	not provided
1489	c.5468-3C>T	Benign	not provided
1490	c.5468-3C>G	Benign	not provided
1491	c.5468-3C>A	Pathogenic	not provided
1492	c.5468-4C>T	Benign	not provided
1493	c.5468-4C>G	Benign	not provided
1494	c.5468-5T>C	Benign	not provided
1495	c.5468-5T>A	Benign	not provided
1496	c.5468-6C>T	Benign	not provided
1497	c.5468-6C>G	Benign	not provided
1498	c.5468-6C>A	Pathogenic	not provided
1499	c.5468-7T>G	Benign	not provided
1500	c.5468-7T>C	Benign	not provided
1501	c.5468-7T>A	Benign	not provided
1502	c.5468-8G>C	Benign	not provided
1503	c.5468-8G>A	Pathogenic	not provided
1504	c.5468-9T>G	Benign	not provided
1505	c.5468-9T>C	Benign	not provided
1506	c.5468-9T>A	Benign	not provided
1507	c.5468-10C>G	Benign	not provided
1508	c.5467+25C>T	Benign	not provided
1509	c.5467+25C>G	Benign	not provided
1510	c.5467+24G>A	Pathogenic	not provided
1511	c.5467+24G>T	Benign	not provided

1512	c.5467+24G>C	Benign	not provided
1513	c.5467+23T>G	Benign	not provided
1514	c.5467+23T>C	Benign	not provided
1515	c.5467+23T>A	Benign	not provided
1516	c.5467+22G>T	Benign	not provided
1517	c.5467+22G>A	Pathogenic	not provided
1518	c.5467+21T>G	Benign	not provided
1519	c.5467+21T>C	Benign	not provided
1520	c.5467+21T>A	Benign	not provided
1521	c.5467+20C>T	Benign	not provided
1522	c.5467+20C>G	Benign	not provided
1523	c.5467+20C>A	Pathogenic	not provided
1524	c.5467+19C>T	Benign	not provided
1525	c.5467+19C>G	Benign	not provided
1526	c.5467+19C>A	Pathogenic	not provided
1527	c.5467+18A>T	Benign	not provided
1528	c.5467+18A>G	Pathogenic	not provided
1529	c.5467+18A>C	Benign	not provided
1530	c.5467+17T>G	Benign	not provided
1531	c.5467+17T>C	Benign	not provided
1532	c.5467+17T>A	Benign	not provided
1533	c.5467+16G>T	Benign	not provided
1534	c.5467+16G>C	Benign	not provided
1535	c.5467+16G>A	Pathogenic	not provided
1536	c.5467+15T>G	Benign	not provided
1537	c.5467+15T>A	Benign	not provided
1538	c.5467+14A>T	Benign	not provided
1539	c.5467+14A>C	Benign	not provided
1540	c.5467+13C>G	Benign	not provided
1541	c.5467+13C>A	Benign	not provided
1542	c.5467+13C>T	Benign	not provided
1543	c.5467+12G>T	Benign	not provided
1544	c.5467+12G>C	Benign	not provided
1545	c.5467+11T>G	Benign	not provided
1546	c.5467+11T>C	Benign	not provided
1547	c.5467+11T>A	Benign	not provided
1548	c.5467+10C>T	Benign	not provided
1549	c.5467+10C>G	Benign	not provided
1550	c.5467+10C>A	Benign	not provided
1551	c.5467+9C>G	Benign	not provided
1552	c.5467+8G>C	Benign	not provided
1553	c.5467+7T>G	Benign	not provided
1554	c.5467+7T>C	Benign	not provided
1555	c.5467+7T>A	Benign	not provided
1556	c.5467+6G>T	Pathogenic	not provided
1557	c.5467+6G>C	Benign	not provided
1558	c.5467+6G>A	Pathogenic	not provided
1559	c.5467+5G>A	Pathogenic	not provided
1560	c.5467+4A>T	Pathogenic	not provided
1561	c.5467+4A>C	Benign	not provided
1562	c.5467+3A>T	Benign	not provided
1563	c.5467+3A>G	Pathogenic	not provided
1564	c.5467+2T>A	Pathogenic	not provided
1565	c.5467+1G>T	Pathogenic	not provided

1566	c.5467+1G>C	Pathogenic	not provided
1567	c.5467G>T	Benign	not provided
1568	c.5467G>C	Benign	not provided
1569	c.5466T>G	Benign	not provided
1570	c.5466T>A	Benign	not provided
1571	c.5465A>T	Benign	not provided
1572	c.5465A>G	Benign	not provided
1573	c.5465A>C	Benign	not provided
1574	c.5464C>T	Benign	not provided
1575	c.5464C>G	Benign	not provided
1576	c.5464C>A	Benign	not provided
1577	c.5463C>T	Benign	not provided
1578	c.5463C>G	Benign	not provided
1579	c.5463C>A	Benign	not provided
1580	c.5462T>G	Benign	not provided
1581	c.5462T>C	Benign	not provided
1582	c.5462T>A	Benign	not provided
1583	c.5461T>G	Benign	not provided
1584	c.5461T>C	Benign	not provided
1585	c.5461T>A	Pathogenic	not provided
1586	c.5460C>T	Benign	not provided
1587	c.5460C>G	Benign	not provided
1588	c.5460C>A	Benign	not provided
1589	c.5459G>T	Benign	not provided
1590	c.5459G>C	Benign	not provided
1591	c.5459G>A	Benign	not provided
1592	c.5458G>C	Benign	not provided
1593	c.5458G>T	Benign	not provided
1594	c.5457T>G	Benign	not provided
1595	c.5457T>A	Benign	not provided
1596	c.5456A>T	Benign	not provided
1597	c.5456A>C	Benign	not provided
1598	c.5455A>T	Pathogenic	not provided
1599	c.5455A>G	Benign	not provided
1600	c.5455A>C	Benign	not provided
1601	c.5454C>G	Benign	not provided
1602	c.5454C>A	Benign	not provided
1603	c.5453A>T	Benign	not provided
1604	c.5453A>C	Benign	not provided
1605	c.5452G>T	Pathogenic	not provided
1606	c.5452G>C	Benign	not provided
1607	c.5452G>A	Pathogenic	not provided
1608	c.5451G>T	Benign	not provided
1609	c.5451G>C	Benign	not provided
1610	c.5450A>G	Pathogenic	not provided
1611	c.5450A>C	Benign	not provided
1612	c.5450A>T	Pathogenic	not provided
1613	c.5449G>C	Benign	not provided
1614	c.5449G>A	Pathogenic	not provided
1615	c.5448A>T	Pathogenic	not provided
1616	c.5448A>C	Benign	not provided
1617	c.5447C>T	Benign	not provided
1618	c.5446A>T	Benign	not provided
1619	c.5446A>G	Benign	not provided

1620	c.5446A>C	Benign	not provided
1621	c.5445G>T	Pathogenic	not provided
1622	c.5445G>C	Benign	not provided
1623	c.5444G>T	Benign	not provided
1624	c.5443T>G	Benign	not provided
1625	c.5443T>C	Benign	not provided
1626	c.5443T>A	Benign	not provided
1627	c.5442C>T	Benign	not provided
1628	c.5442C>G	Benign	not provided
1629	c.5442C>A	Benign	not provided
1630	c.5441C>T	Benign	not provided
1631	c.5441C>G	Benign	not provided
1632	c.5440G>T	Benign	not provided
1633	c.5440G>C	Benign	not provided
1634	c.5440G>A	Pathogenic	not provided
1635	c.5439T>G	Benign	not provided
1636	c.5439T>A	Benign	not provided
1637	c.5438A>G	Benign	not provided
1638	c.5438A>C	Benign	not provided
1639	c.5437G>C	Benign	not provided
1640	c.5437G>A	Pathogenic	not provided
1641	c.5436A>T	Pathogenic	not provided
1642	c.5436A>G	Pathogenic	not provided
1643	c.5436A>C	Benign	not provided
1644	c.5435C>T	Benign	not provided
1645	c.5435C>G	Benign	not provided
1646	c.5435C>A	Pathogenic	not provided
1647	c.5434C>A	Benign	not provided
1648	c.5434C>T	Benign	not provided
1649	c.5433G>T	Benign	not provided
1650	c.5433G>A	Pathogenic	not provided
1651	c.5432A>T	Pathogenic	not provided
1652	c.5432A>C	Benign	not provided
1653	c.5431C>G	Benign	not provided
1654	c.5431C>A	Benign	not provided
1655	c.5430G>T	Benign	not provided
1656	c.5430G>C	Benign	not provided
1657	c.5429T>A	Benign	not provided
1658	c.5428G>T	Benign	not provided
1659	c.5428G>C	Benign	not provided
1660	c.5428G>A	Pathogenic	not provided
1661	c.5427T>G	Benign	not provided
1662	c.5427T>C	Benign	not provided
1663	c.5427T>A	Pathogenic	not provided
1664	c.5422G>C	Benign	not provided
1665	c.5421T>G	Benign	not provided
1666	c.5421T>C	Benign	not provided
1667	c.5421T>A	Pathogenic	not provided
1668	c.5420T>G	Benign	not provided
1669	c.5420T>C	Benign	not provided
1670	c.5420T>A	Benign	not provided
1671	c.5419A>C	Benign	not provided
1672	c.5418A>T	Pathogenic	not provided
1673	c.5418A>G	Benign	not provided

1674	c.5417C>T		Benign	not provided
1675	c.5417C>G		Benign	not provided
1676	c.5417C>A		Benign	not provided
1677	c.5416C>A		Pathogenic	not provided
1678	c.5415C>G		Benign	not provided
1679	c.5415C>A		Benign	not provided
1680	c.5414A>T		Benign	not provided
1681	c.5414A>G		Benign	not provided
1682	c.5414A>C	rs397509281	Benign	not provided
1683	c.5413C>G		Benign	not provided
1684	c.5413C>A		Benign	not provided
1685	c.5412C>G		Benign	not provided
1686	c.5412C>A		Benign	not provided
1687	c.5411T>G		Benign	not provided
1688	c.5411T>C		Benign	not provided
1689	c.5410G>A		Pathogenic	not provided
1690	c.5410G>T		Benign	not provided
1691	c.5410G>C		Benign	not provided
1692	c.5409T>G		Benign	not provided
1693	c.5409T>C		Benign	not provided
1694	c.5409T>A		Benign	not provided
1695	c.5408G>A		Pathogenic	not provided
1696	c.5407G>C		Pathogenic	not provided
1697	c.5407-1G>T		Benign	not provided
1698	c.5407-2A>C		Benign	not provided
1699	c.5407-3C>T		Benign	not provided
1700	c.5407-3C>G		Benign	not provided
1701	c.5407-3C>A		Pathogenic	not provided
1702	c.5407-4C>T		Benign	not provided
1703	c.5407-4C>A		Benign	not provided
1704	c.5407-5T>G		Benign	not provided
1705	c.5407-5T>C		Benign	not provided
1706	c.5407-5T>A		Benign	not provided
1707	c.5407-6A>T		Benign	not provided
1708	c.5407-6A>G		Benign	not provided
1709	c.5407-6A>C		Benign	not provided
1710	c.5407-7G>T		Pathogenic	not provided
1711	c.5407-7G>C		Benign	not provided
1712	c.5407-8G>C		Benign	not provided
1713	c.5407-8G>A		Benign	not provided
1714	c.5407-9G>T		Benign	not provided
1715	c.5407-10G>T		Benign	not provided
1716	c.5407-10G>C		Benign	not provided
1717	c.5407-11T>G		Benign	not provided
1718	c.5407-11T>C		Benign	not provided
1719	c.5407-11T>A		Benign	not provided
1720	c.5407-12C>T		Benign	not provided
1721	c.5407-12C>G		Benign	not provided
1722	c.5407-12C>A		Benign	not provided
1723	c.5407-13C>T		Benign	not provided
1724	c.5407-13C>G		Benign	not provided
1725	c.5407-13C>A		Benign	not provided
1726	c.5407-14T>G		Benign	not provided
1727	c.5407-14T>C		Benign	not provided

1728	c.5407-14T>A	Benign	not provided
1729	c.5406+18T>G	Benign	not provided
1730	c.5406+18T>C	Benign	not provided
1731	c.5406+18T>A	Benign	not provided
1732	c.5406+17C>G	Benign	not provided
1733	c.5406+17C>A	Benign	not provided
1734	c.5406+16C>T	Benign	not provided
1735	c.5406+16C>G	Benign	not provided
1736	c.5406+16C>A	Pathogenic	not provided
1737	c.5406+15C>G	Benign	not provided
1738	c.5406+14G>C	Benign	not provided
1739	c.5406+14G>A	Pathogenic	not provided
1740	c.5406+13T>G	Benign	not provided
1741	c.5406+13T>C	Benign	not provided
1742	c.5406+13T>A	Benign	not provided
1743	c.5406+12G>C	Benign	not provided
1744	c.5406+11G>C	Pathogenic	not provided
1745	c.5406+11G>A	Pathogenic	not provided
1746	c.5406+10G>C	Pathogenic	not provided
1747	c.5406+10G>A	Pathogenic	not provided
1748	c.5406+9T>G	Benign	not provided
1749	c.5406+9T>A	Pathogenic	not provided
1750	c.5406+8T>G	Benign	not provided
1751	c.5406+8T>A	Benign	not provided
1752	c.5406+7A>T	Benign	not provided
1753	c.5406+7A>C	Benign	not provided
1754	c.5406+6T>G	Benign	not provided
1755	c.5406+6T>C	Benign	not provided
1756	c.5406+4A>T	Pathogenic	not provided
1757	c.5406+4A>C	Benign	not provided
1758	c.5406+3A>C	Benign	not provided
1759	c.5406+3A>G	Pathogenic	not provided
1760	c.5406+2T>G	Benign	not provided
1761	c.5406+2T>C	Benign	not provided
1762	c.5406+2T>A	Benign	not provided
1763	c.5406+1G>T	Benign	not provided
1764	c.5406+1G>C	Benign	not provided
1765	c.5406A>T	Pathogenic	not provided
1766	c.5406A>G	Pathogenic	not provided
1767	c.5405C>T	Benign	not provided
1768	c.5405C>G	Benign	not provided
1769	c.5405C>A	Pathogenic	not provided
1770	c.5404A>T	Benign	not provided
1771	c.5404A>G	Benign	not provided
1772	c.5403C>T	Benign	not provided
1773	c.5403C>G	Benign	not provided
1774	c.5403C>A	Benign	not provided
1775	c.5402G>T	Benign	not provided
1776	c.5402G>C	Benign	not provided
1777	c.5401G>T	Benign	not provided
1778	c.5401G>C	Benign	not provided
1779	c.5400T>G	Benign	not provided
1780	c.5400T>C	Benign	not provided
1781	c.5400T>A	Benign	not provided

1782	c.5399T>C		Benign	not provided
1783	c.5399T>A		Benign	not provided
1784	c.5398C>G		Benign	not provided
1785	c.5398C>A		Benign	not provided
1786	c.5398C>T		Benign	not provided
1787	c.5397C>G		Pathogenic	not provided
1788	c.5397C>A		Pathogenic	not provided
1789	c.5396C>G		Benign	not provided
1790	c.5396C>A		Benign	not provided
1791	c.5395A>T		Benign	not provided
1792	c.5395A>G		Pathogenic	not provided
1793	c.5395A>C		Benign	not provided
1794	c.5394C>G		Benign	not provided
1795	c.5394C>A		Pathogenic	not provided
1796	c.5393T>G		Pathogenic	not provided
1797	c.5393T>C		Benign	not provided
1798	c.5393T>A		Benign	not provided
1799	c.5392T>G		Benign	not provided
1800	c.5392T>C		Benign	not provided
1801	c.5392T>A		Benign	not provided
1802	c.5391A>T		Benign	not provided
1803	c.5391A>G		Pathogenic	not provided
1804	c.5391A>C		Benign	not provided
1805	c.5389T>G		Benign	not provided
1806	c.5389T>C		Benign	not provided
1807	c.5389T>A		Benign	not provided
1808	c.5388A>T		Benign	not provided
1809	c.5388A>C		Benign	not provided
1810	c.5387C>G		Pathogenic	not provided
1811	c.5386T>G		Benign	not provided
1812	c.5386T>C		Benign	not provided
1813	c.5386T>A		Benign	not provided
1814	c.5385T>G		Benign	not provided
1815	c.5385T>C		Benign	not provided
1816	c.5385T>A		Benign	not provided
1817	c.5384T>G		Benign	not provided
1818	c.5384T>C		Benign	not provided
1819	c.5384T>A		Benign	not provided
1820	c.5383C>G		Benign	not provided
1821	c.5382G>T	rs397509275	Benign	not provided
1822	c.5381A>T		Pathogenic	not provided
1823	c.5381A>G		Pathogenic	not provided
1824	c.5381A>C		Benign	not provided
1825	c.5380G>C		Benign	not provided
1826	c.5380G>A		Pathogenic	not provided
1827	c.5379G>T		Pathogenic	not provided
1828	c.5379G>C		Benign	not provided
1829	c.5379G>A		Benign	not provided
1830	c.5378A>T		Pathogenic	not provided
1831	c.5378A>G		Benign	not provided
1832	c.5377A>C		Benign	not provided
1833	c.5377A>G		Benign	not provided
1834	c.5376G>T		Pathogenic	not provided
1835	c.5376G>C		Benign	not provided

1836	c.5375T>G		Benign	not provided
1837	c.5375T>C		Benign	not provided
1838	c.5375T>A		Benign	not provided
1839	c.5374G>T		Benign	not provided
1840	c.5373G>T		Benign	not provided
1841	c.5373G>C		Benign	not provided
1842	c.5373G>A		Pathogenic	not provided
1843	c.5372T>G		Benign	not provided
1844	c.5372T>A	rs864622244	Benign	not provided
1845	c.5371G>C		Benign	not provided
1846	c.5370T>G		Benign	not provided
1847	c.5370T>C		Benign	not provided
1848	c.5370T>A		Benign	not provided
1849	c.5369C>T		Benign	not provided
1850	c.5369C>G		Pathogenic	not provided
1851	c.5369C>A		Pathogenic	not provided
1852	c.5368T>G		Benign	not provided
1853	c.5368T>A		Benign	not provided
1854	c.5367T>G		Benign	not provided
1855	c.5367T>A		Pathogenic	not provided
1856	c.5366C>G		Benign	not provided
1857	c.5366C>A		Benign	not provided
1858	c.5366C>T		Benign	not provided
1859	c.5364T>G		Benign	not provided
1860	c.5364T>C		Benign	not provided
1861	c.5364T>A		Benign	not provided
1862	c.5362G>C		Benign	not provided
1863	c.5362G>A		Pathogenic	not provided
1864	c.5361T>G		Benign	not provided
1865	c.5361T>C		Benign	not provided
1866	c.5361T>A		Benign	not provided
1867	c.5360G>T		Benign	not provided
1868	c.5360G>C		Pathogenic	not provided
1869	c.5359T>G		Benign	not provided
1870	c.5359T>C		Benign	not provided
1871	c.5358G>T		Benign	not provided
1872	c.5358G>A		Pathogenic	not provided
1873	c.5357T>G		Benign	not provided
1874	c.5357T>A		Benign	not provided
1875	c.5356C>G		Benign	not provided
1876	c.5356C>A		Benign	not provided
1877	c.5355G>C		Benign	not provided
1878	c.5355G>A		Pathogenic	not provided
1879	c.5355G>T	rs397509269	Benign	not provided
1880	c.5354A>T		Pathogenic	not provided
1881	c.5354A>G		Pathogenic	not provided
1882	c.5353C>G		Benign	not provided
1883	c.5353C>A		Pathogenic	not provided
1884	c.5352A>C		Benign	not provided
1885	c.5351T>G		Pathogenic	not provided
1886	c.5351T>C		Benign	not provided
1887	c.5351T>A		Pathogenic	not provided
1888	c.5350G>T		Pathogenic	not provided
1889	c.5350G>C		Pathogenic	not provided

1890	c.5349G>T	Benign	not provided
1891	c.5349G>C	Benign	not provided
1892	c.5348T>G	Benign	not provided
1893	c.5348T>A	Benign	not provided
1894	c.5347A>T	Benign	not provided
1895	c.5346G>T	Pathogenic	not provided
1896	c.5346G>C	Benign	not provided
1897	c.5345G>T	Benign	not provided
1898	c.5345G>C	Benign	not provided
1899	c.5344T>G	Benign	not provided
1900	c.5344T>C	Benign	not provided
1901	c.5344T>A	Benign	not provided
1902	c.5343A>T	Pathogenic	not provided
1903	c.5343A>G	Benign	not provided
1904	c.5342A>T	Pathogenic	not provided
1905	c.5342A>C	Benign	not provided
1906	c.5341G>C	Benign	not provided
1907	c.5338C>G	Benign	not provided
1908	c.5337A>T	Benign	not provided
1909	c.5337A>C	Benign	not provided
1910	c.5336A>T	Pathogenic	not provided
1911	c.5336A>G	Pathogenic	not provided
1912	c.5336A>C	Benign	not provided
1913	c.5335C>A	Pathogenic	not provided
1914	c.5334T>G	Benign	not provided
1915	c.5334T>A	Benign	not provided
1916	c.5333A>T	Benign	not provided
1917	c.5333A>C	Benign	not provided
1918	c.5333-3T>C	Benign	not provided
1919	c.5333-3T>A	Benign	not provided
1920	c.5333-4T>G	Benign	not provided
1921	c.5333-4T>C	Benign	not provided
1922	c.5333-4T>A	Pathogenic	not provided
1923	c.5333-5T>G	Pathogenic	not provided
1924	c.5333-5T>C	Benign	not provided
1925	c.5333-5T>A	Pathogenic	not provided
1926	c.5333-6T>C	Benign	not provided
1927	c.5333-6T>A	Benign	not provided
1928	c.5333-7A>T	Benign	not provided
1929	c.5333-7A>C	Benign	not provided
1930	c.5333-8C>G	Benign	not provided
1931	c.5333-8C>A	Benign	not provided
1932	c.5333-9C>T	Benign	not provided
1933	c.5333-9C>G	Benign	not provided
1934	c.5333-9C>A	Benign	not provided
1935	c.5333-10T>G	Benign	not provided
1936	c.5333-10T>C	Benign	not provided
1937	c.5333-10T>A	Benign	not provided
1938	c.5333-11G>T	Benign	not provided
1939	c.5333-11G>C	Benign	not provided
1940	c.5333-12T>A	Benign	not provided
1941	c.5333-12T>G	Benign	not provided
1942	c.5333-13A>T	Benign	not provided
1943	c.5333-13A>G	Benign	not provided

1944	c.5333-13A>C		Benign	not provided
1945	c.5332+23G>T		Pathogenic	not provided
1946	c.5332+23G>C		Benign	not provided
1947	c.5332+23G>A		Pathogenic	not provided
1948	c.5332+22A>T		Pathogenic	not provided
1949	c.5332+22A>G		Pathogenic	not provided
1950	c.5332+22A>C		Benign	not provided
1951	c.5332+21C>T		Benign	not provided
1952	c.5332+21C>G		Benign	not provided
1953	c.5332+21C>A		Pathogenic	not provided
1954	c.5332+20C>T		Benign	not provided
1955	c.5332+19C>A		Pathogenic	not provided
1956	c.5332+18C>T		Benign	not provided
1957	c.5332+18C>G		Benign	not provided
1958	c.5332+17A>T		Benign	not provided
1959	c.5332+17A>G		Benign	not provided
1960	c.5332+17A>C		Benign	not provided
1961	c.5332+16A>T		Pathogenic	not provided
1962	c.5332+16A>C		Benign	not provided
1963	c.5332+15G>T		Pathogenic	not provided
1964	c.5332+15G>A		Pathogenic	not provided
1965	c.5332+14A>T		Pathogenic	not provided
1966	c.5332+14A>G		Pathogenic	not provided
1967	c.5332+14A>C		Benign	not provided
1968	c.5332+13G>A		Pathogenic	not provided
1969	c.5332+12G>T		Benign	not provided
1970	c.5332+12G>A		Pathogenic	not provided
1971	c.5332+11G>T		Benign	not provided
1972	c.5332+11G>C		Benign	not provided
1973	c.5332+10T>G		Benign	not provided
1974	c.5332+10T>C		Benign	not provided
1975	c.5332+10T>A		Benign	not provided
1976	c.5332+9C>T		Benign	not provided
1977	c.5332+9C>G		Benign	not provided
1978	c.5332+9C>A		Benign	not provided
1979	c.5332+8C>G		Benign	not provided
1980	c.5332+8C>A		Benign	not provided
1981	c.5332+7G>C		Benign	not provided
1982	c.5332+7G>A		Pathogenic	not provided
1983	c.5332+6A>T		Pathogenic	not provided
1984	c.5332+6A>G		Pathogenic	not provided
1985	c.5332+6A>C		Benign	not provided
1986	c.5332+5G>T		Pathogenic	not provided
1987	c.5332+5G>C		Benign	not provided
1988	c.5332+4A>T		Pathogenic	not provided
1989	c.5332+4A>C		Benign	not provided
1990	c.5332+3A>C		Benign	not provided
1991	c.5332+3A>T		Benign	not provided
1992	c.5332G>T	rs80357112	Benign	not provided
1993	c.5331A>T		Pathogenic	not provided
1994	c.5331A>G		Pathogenic	not provided
1995	c.5331A>C		Benign	not provided
1996	c.5330C>G		Benign	not provided
1997	c.5330C>A		Benign	not provided

1998	c.5329A>G	Benign	not provided
1999	c.5329A>C	Benign	not provided
2000	c.5328C>A	Benign	not provided
2001	c.5327C>G	Benign	not provided
2002	c.5326C>G	Benign	not provided
2003	c.5326C>A	Benign	not provided
2004	c.5325G>T	Benign	not provided
2005	c.5325G>C	Benign	not provided
2006	c.5323A>T	Benign	not provided
2007	c.5323A>G	Benign	not provided
2008	c.5323A>C	Benign	not provided
2009	c.5322C>T	Benign	not provided
2010	c.5322C>G	Benign	not provided
2011	c.5322C>A	Benign	not provided
2012	c.5321A>T	Benign	not provided
2013	c.5321A>C	Benign	not provided
2014	c.5320A>T	Pathogenic	not provided
2015	c.5320A>G	Benign	not provided
2016	c.5319C>T	Benign	not provided
2017	c.5319C>A	Benign	not provided
2018	c.5318C>G	Benign	not provided
2019	c.5318C>A	Pathogenic	not provided
2020	c.5317A>G	Pathogenic	not provided
2021	c.5317A>C	Benign	not provided
2022	c.5316C>T	Benign	not provided
2023	c.5316C>A	Pathogenic	not provided
2024	c.5315T>C	Benign	not provided
2025	c.5315T>A	Benign	not provided
2026	c.5314T>G	Benign	not provided
2027	c.5314T>C	Benign	not provided
2028	c.5314T>A	Benign	not provided
2029	c.5313C>G	Benign	not provided
2030	c.5313C>A	Benign	not provided
2031	c.5312C>A	Pathogenic	not provided
2032	c.5311C>G	Benign	not provided
2033	c.5311C>A	Benign	not provided
2034	c.5310G>T	Pathogenic	not provided
2035	c.5309G>A	Pathogenic	not provided
2036	c.5308G>T	Benign	not provided
2037	c.5308G>C	Benign	not provided
2038	c.5308G>A	Pathogenic	not provided
2039	c.5307T>C	Benign	not provided
2040	c.5306A>T	Benign	not provided
2041	c.5306A>C	Benign	not provided
2042	c.5305T>G	Benign	not provided
2043	c.5305T>A	Benign	not provided
2044	c.5304C>G	Benign	not provided
2045	c.5304C>A	Benign	not provided
2046	c.5303G>T	Benign	not provided
2047	c.5303G>C	Benign	not provided
2048	c.5302T>C	Benign	not provided
2049	c.5302T>A	Pathogenic	not provided
2050	c.5301T>G	Benign	not provided
2051	c.5301T>C	Benign	not provided

2052	c.5300G>T	Benign	not provided
2053	c.5300G>A	Pathogenic	not provided
2054	c.5299T>G	Benign	not provided
2055	c.5299T>A	Benign	not provided
2056	c.5298C>T	Benign	not provided
2057	c.5298C>G	Pathogenic	not provided
2058	c.5298C>A	Pathogenic	not provided
2059	c.5297T>C	Benign	not provided
2060	c.5296A>T	Benign	not provided
2061	c.5296A>C	Benign	not provided
2062	c.5295A>T	Pathogenic	not provided
2063	c.5295A>G	Benign	not provided
2064	c.5295A>C	Benign	not provided
2065	c.5294A>T	Pathogenic	not provided
2066	c.5294A>G	Benign	not provided
2067	c.5294A>C	Benign	not provided
2068	c.5293G>C	Benign	not provided
2069	c.5293G>A	Pathogenic	not provided
2070	c.5292A>T	Pathogenic	not provided
2071	c.5292A>G	Pathogenic	not provided
2072	c.5292A>C	Benign	not provided
2073	c.5291T>A	Pathogenic	not provided
2074	c.5290C>G	Benign	not provided
2075	c.5290C>A	Benign	not provided
2076	c.5288G>C	Pathogenic	not provided
2077	c.5286G>T	Benign	not provided
2078	c.5286G>C	Pathogenic	not provided
2079	c.5286G>A	Benign	not provided
2080	c.5284A>T	Pathogenic	not provided
2081	c.5284A>G	Pathogenic	not provided
2082	c.5284A>C	Pathogenic	not provided
2083	c.5283C>T	Benign	not provided
2084	c.5283C>G	Pathogenic	not provided
2085	c.5283C>A	Pathogenic	not provided
2086	c.5282T>A	Benign	not provided
2087	c.5281T>G	Benign	not provided
2088	c.5281T>A	Benign	not provided
2089	c.5280C>G	Benign	not provided
2090	c.5279T>G	Benign	not provided
2091	c.5279T>C	Benign	not provided
2092	c.5279T>A	Benign	not provided
2093	c.5278A>G	Benign	not provided
2094	c.5278A>C	Benign	not provided
2095	c.5278A>T	Benign	not provided
2096	c.5278-2A>C	Benign	not provided
2097	c.5278-3C>G	Benign	not provided
2098	c.5278-3C>A	Pathogenic	not provided
2099	c.5278-4C>T	Benign	not provided
2100	c.5278-4C>G	Benign	not provided
2101	c.5278-4C>A	Benign	not provided
2102	c.5278-5T>G	Benign	not provided
2103	c.5278-5T>C	Benign	not provided
2104	c.5278-5T>A	Benign	not provided
2105	c.5278-6T>G	Benign	not provided

2106	c.5278-6T>A	Benign	not provided
2107	c.5278-7C>T	Benign	not provided
2108	c.5278-7C>G	Benign	not provided
2109	c.5278-7C>A	Benign	not provided
2110	c.5278-8T>G	Benign	not provided
2111	c.5278-8T>C	Benign	not provided
2112	c.5278-8T>A	Benign	not provided
2113	c.5278-9T>G	Benign	not provided
2114	c.5278-9T>C	Benign	not provided
2115	c.5278-9T>A	Benign	not provided
2116	c.5278-10C>T	Benign	not provided
2117	c.5278-10C>G	Benign	not provided
2118	c.5278-10C>A	Benign	not provided
2119	c.5278-11T>G	Benign	not provided
2120	c.5278-11T>A	Benign	not provided
2121	c.5278-12C>T	Benign	not provided
2122	c.5278-12C>A	Benign	not provided
2123	c.5278-13T>G	Benign	not provided
2124	c.5278-13T>C	Benign	not provided
2125	c.5278-13T>A	Benign	not provided
2126	c.5278-14C>T	Benign	not provided
2127	c.5278-14C>A	Benign	not provided
2128	c.5278-15C>T	Benign	not provided
2129	c.5278-15C>G	Benign	not provided
2130	c.5278-15C>A	Benign	not provided
2131	c.5278-16T>G	Benign	not provided
2132	c.5278-16T>A	Benign	not provided
2133	c.5278-17T>G	Benign	not provided
2134	c.5278-17T>C	Benign	not provided
2135	c.5278-17T>A	Benign	not provided
2136	c.5278-18C>T	Benign	not provided
2137	c.5278-18C>A	Benign	not provided
2138	c.5278-19T>G	Benign	not provided
2139	c.5278-19T>C	Benign	not provided
2140	c.5278-20C>G	Benign	not provided
2141	c.5278-20C>A	Benign	not provided
2142	c.5278-21T>A	Benign	not provided
2143	c.5278-21T>G	Benign	not provided
2144	c.5278-21T>C	Benign	not provided
2145	c.5278-22C>G	Benign	not provided
2146	c.5278-22C>A	Benign	not provided
2147	c.5277+10C>T	Benign	not provided
2148	c.5277+10C>G	Benign	not provided
2149	c.5277+9C>T	Benign	not provided
2150	c.5277+9C>A	Benign	not provided
2151	c.5277+8T>G	Benign	not provided
2152	c.5277+8T>C	Benign	not provided
2153	c.5277+8T>A	Benign	not provided
2154	c.5277+7C>G	Benign	not provided
2155	c.5277+7C>A	Benign	not provided
2156	c.5277+6G>C	Pathogenic	not provided
2157	c.5277+6G>A	Benign	not provided
2158	c.5277+5A>G	Benign	not provided
2159	c.5277+5A>C	Benign	not provided

2160	c.5277+4A>C		Benign	not provided
2161	c.5277+3A>T		Benign	not provided
2162	c.5277+2T>G		Pathogenic	not provided
2163	c.5277+2T>C		Benign	not provided
2164	c.5277+2T>A		Pathogenic	not provided
2165	c.5277G>C		Benign	not provided
2166	c.5276A>T		Pathogenic	not provided
2167	c.5276A>C		Benign	not provided
2168	c.5275A>T		Pathogenic	not provided
2169	c.5275A>G		Benign	not provided
2170	c.5275A>C		Benign	not provided
2171	c.5274A>T		Pathogenic	not provided
2172	c.5274A>C		Benign	not provided
2173	c.5273G>T		Pathogenic	not provided
2174	c.5273G>C		Benign	not provided
2175	c.5273G>A		Pathogenic	not provided
2176	c.5272A>T		Pathogenic	not provided
2177	c.5272A>C		Benign	not provided
2178	c.5271C>T		Benign	not provided
2179	c.5271C>G		Benign	not provided
2180	c.5270A>T		Benign	not provided
2181	c.5270A>G		Benign	not provided
2182	c.5270A>C		Benign	not provided
2183	c.5268G>T		Benign	not provided
2184	c.5268G>C		Benign	not provided
2185	c.5267A>C		Benign	not provided
2186	c.5267A>T		Pathogenic	not provided
2187	c.5266C>G		Benign	not provided
2188	c.5266C>A		Pathogenic	not provided
2189	c.5265C>G		Benign	not provided
2190	c.5265C>A		Pathogenic	not provided
2191	c.5264C>G		Pathogenic	not provided
2192	c.5264C>A		Benign	not provided
2193	c.5263T>G		Benign	not provided
2194	c.5263T>C		Benign	not provided
2195	c.5263T>A		Benign	not provided
2196	c.5262A>T		Benign	not provided
2197	c.5262A>G		Benign	not provided
2198	c.5262A>C		Benign	not provided
2199	c.5261A>T		Pathogenic	not provided
2200	c.5261A>G		Benign	not provided
2201	c.5261A>C		Benign	not provided
2202	c.5260G>A	rs80357432	Pathogenic	not provided
2203	c.5259A>C		Benign	not provided
2204	c.5258G>T		Pathogenic	not provided
2205	c.5258G>A	rs397509246	Pathogenic	not provided
2206	c.5257A>T		Pathogenic	not provided
2207	c.5257A>C		Benign	not provided
2208	c.5256A>T		Pathogenic	not provided
2209	c.5256A>G		Benign	not provided
2210	c.5255C>A		Benign	not provided
2211	c.5254G>T		Benign	not provided
2212	c.5253A>T		Pathogenic	not provided
2213	c.5253A>G		Pathogenic	not provided

2214	c.5253A>C		Benign	not provided
2215	c.5250G>T		Benign	not provided
2216	c.5250G>C		Benign	not provided
2217	c.5249A>T		Pathogenic	not provided
2218	c.5249A>G		Benign	not provided
2219	c.5249A>C		Benign	not provided
2220	c.5248A>T		Pathogenic	not provided
2221	c.5248A>G		Benign	not provided
2222	c.5248A>C		Benign	not provided
2223	c.5247A>T		Pathogenic	not provided
2224	c.5247A>G		Benign	not provided
2225	c.5247A>C		Benign	not provided
2226	c.5246C>A		Benign	not provided
2227	c.5245C>T		Benign	not provided
2228	c.5245C>G	rs397509244	Benign	not provided
2229	c.5244T>G		Benign	not provided
2230	c.5244T>C		Benign	not provided
2231	c.5244T>A		Benign	not provided
2232	c.5243G>C		Benign	not provided
2233	c.5242G>A	rs397507245	Pathogenic	not provided
2234	c.5241A>T		Pathogenic	not provided
2235	c.5241A>G		Benign	not provided
2236	c.5241A>C	rs397509242	Benign	not provided
2237	c.5240A>T		Pathogenic	not provided
2238	c.5240A>G		Benign	not provided
2239	c.5240A>C		Benign	not provided
2240	c.5239C>G		Benign	not provided
2241	c.5239C>A		Benign	not provided
2242	c.5238C>T		Benign	not provided
2243	c.5238C>A		Benign	not provided
2244	c.5237A>T		Benign	not provided
2245	c.5237A>G		Benign	not provided
2246	c.5235C>G		Benign	not provided
2247	c.5235C>A		Benign	not provided
2248	c.5235C>T		Benign	not provided
2249	c.5234A>T		Benign	not provided
2250	c.5234A>C		Benign	not provided
2251	c.5233A>T		Pathogenic	not provided
2252	c.5233A>G		Benign	not provided
2253	c.5233A>C		Benign	not provided
2254	c.5232A>T		Pathogenic	not provided
2255	c.5232A>G		Benign	not provided
2256	c.5232A>C		Benign	not provided
2257	c.5231G>T		Pathogenic	not provided
2258	c.5231G>C		Benign	not provided
2259	c.5230A>T		Pathogenic	not provided
2260	c.5230A>G		Benign	not provided
2261	c.5230A>C		Benign	not provided
2262	c.5229A>T		Pathogenic	not provided
2263	c.5229A>G		Benign	not provided
2264	c.5229A>C		Benign	not provided
2265	c.5228G>T		Pathogenic	not provided
2266	c.5228G>A		Pathogenic	not provided
2267	c.5227G>C		Benign	not provided

2268	c.5227G>A		Pathogenic	not provided
2269	c.5226T>G		Benign	not provided
2270	c.5226T>C		Benign	not provided
2271	c.5226T>A		Benign	not provided
2272	c.5225A>T		Benign	not provided
2273	c.5225A>C		Benign	not provided
2274	c.5224A>T		Pathogenic	not provided
2275	c.5224A>C		Benign	not provided
2276	c.5223C>T		Benign	not provided
2277	c.5223C>G		Pathogenic	not provided
2278	c.5223C>A		Pathogenic	not provided
2279	c.5222T>C		Benign	not provided
2280	c.5222T>A		Benign	not provided
2281	c.5221G>C		Benign	not provided
2282	c.5219T>G		Benign	not provided
2283	c.5218G>C		Benign	not provided
2284	c.5217T>C		Benign	not provided
2285	c.5217T>A		Benign	not provided
2286	c.5216A>C		Benign	not provided
2287	c.5214A>T		Pathogenic	not provided
2288	c.5214A>G		Pathogenic	not provided
2289	c.5214A>C		Benign	not provided
2290	c.5213G>C		Benign	not provided
2291	c.5212G>C		Benign	not provided
2292	c.5211A>C		Benign	not provided
2293	c.5211A>T		Pathogenic	not provided
2294	c.5210G>T		Pathogenic	not provided
2295	c.5210G>C		Benign	not provided
2296	c.5210G>A		Pathogenic	not provided
2297	c.5209A>G		Pathogenic	not provided
2298	c.5209A>C		Benign	not provided
2299	c.5208C>T		Benign	not provided
2300	c.5208C>G		Pathogenic	not provided
2301	c.5208C>A		Pathogenic	not provided
2302	c.5207T>A		Benign	not provided
2303	c.5206G>T		Benign	not provided
2304	c.5206G>C		Benign	not provided
2305	c.5206G>A		Benign	not provided
2306	c.5205A>G		Benign	not provided
2307	c.5205A>C		Benign	not provided
2308	c.5204A>T		Pathogenic	not provided
2309	c.5204A>G		Benign	not provided
2310	c.5204A>C		Benign	not provided
2311	c.5203G>T		Pathogenic	not provided
2312	c.5203G>C		Benign	not provided
2313	c.5203G>A	rs397509238	Pathogenic	not provided
2314	c.5202T>A		Pathogenic	not provided
2315	c.5201T>A		Benign	not provided
2316	c.5201T>C	rs397509237	Benign	not provided
2317	c.5200T>G		Benign	not provided
2318	c.5199T>G		Benign	not provided
2319	c.5199T>C		Benign	not provided
2320	c.5199T>A		Benign	not provided
2321	c.5198A>C		Benign	not provided

2322	c.5197G>T	Pathogenic	not provided
2323	c.5196T>G	Benign	not provided
2324	c.5195A>T	Benign	not provided
2325	c.5195A>C	Benign	not provided
2326	c.5194C>T	Benign	not provided
2327	c.5194C>G	Benign	not provided
2328	c.5194C>A	Benign	not provided
2329	c.5194-2A>T	Pathogenic	not provided
2330	c.5194-3C>T	Benign	not provided
2331	c.5194-3C>G	Pathogenic	not provided
2332	c.5194-3C>A	Pathogenic	not provided
2333	c.5194-4T>A	Benign	not provided
2334	c.5194-4T>G	Benign	not provided
2335	c.5194-5T>G	Benign	not provided
2336	c.5194-5T>C	Benign	not provided
2337	c.5194-5T>A	Benign	not provided
2338	c.5194-6T>G	Benign	not provided
2339	c.5194-6T>C	Benign	not provided
2340	c.5194-6T>A	Benign	not provided
2341	c.5194-7C>T	Benign	not provided
2342	c.5194-7C>G	Benign	not provided
2343	c.5194-7C>A	Benign	not provided
2344	c.5194-8T>G	Benign	not provided
2345	c.5194-8T>C	Benign	not provided
2346	c.5194-8T>A	Benign	not provided
2347	c.5194-9T>G	Benign	not provided
2348	c.5194-9T>C	Benign	not provided
2349	c.5194-9T>A	Pathogenic	not provided
2350	c.5194-10T>G	Benign	not provided
2351	c.5194-10T>A	Benign	not provided
2352	c.5193+30A>T	Pathogenic	not provided
2353	c.5193+30A>G	Pathogenic	not provided
2354	c.5193+30A>C	Benign	not provided
2355	c.5193+29G>T	Pathogenic	not provided
2356	c.5193+29G>C	Benign	not provided
2357	c.5193+29G>A	Pathogenic	not provided
2358	c.5193+28A>T	Pathogenic	not provided
2359	c.5193+28A>G	Pathogenic	not provided
2360	c.5193+28A>C	Benign	not provided
2361	c.5193+27C>G	Benign	not provided
2362	c.5193+26C>T	Benign	not provided
2363	c.5193+26C>G	Benign	not provided
2364	c.5193+26C>A	Benign	not provided
2365	c.5193+25A>T	Benign	not provided
2366	c.5193+25A>G	Benign	not provided
2367	c.5193+25A>C	Benign	not provided
2368	c.5193+24A>T	Benign	not provided
2369	c.5193+24A>G	Pathogenic	not provided
2370	c.5193+24A>C	Benign	not provided
2371	c.5193+23T>G	Benign	not provided
2372	c.5193+23T>C	Benign	not provided
2373	c.5193+23T>A	Benign	not provided
2374	c.5193+22C>G	Benign	not provided
2375	c.5193+22C>A	Benign	not provided

2376	c.5193+21A>T	Benign	not provided
2377	c.5193+21A>G	Benign	not provided
2378	c.5193+21A>C	Benign	not provided
2379	c.5193+20A>T	Pathogenic	not provided
2380	c.5193+20A>C	Benign	not provided
2381	c.5193+19A>T	Pathogenic	not provided
2382	c.5193+19A>C	Benign	not provided
2383	c.5193+18C>T	Benign	not provided
2384	c.5193+18C>G	Benign	not provided
2385	c.5193+18C>A	Benign	not provided
2386	c.5193+17A>T	Benign	not provided
2387	c.5193+17A>G	Pathogenic	not provided
2388	c.5193+17A>C	Benign	not provided
2389	c.5193+16T>G	Benign	not provided
2390	c.5193+16T>C	Benign	not provided
2391	c.5193+16T>A	Benign	not provided
2392	c.5193+15T>A	Benign	not provided
2393	c.5193+15T>G	Benign	not provided
2394	c.5193+15T>C	Benign	not provided
2395	c.5193+14G>T	Benign	not provided
2396	c.5193+14G>C	Benign	not provided
2397	c.5193+14G>A	Pathogenic	not provided
2398	c.5193+13T>G	Benign	not provided
2399	c.5193+13T>C	Benign	not provided
2400	c.5193+12A>T	Benign	not provided
2401	c.5193+12A>G	Benign	not provided
2402	c.5193+12A>C	Benign	not provided
2403	c.5193+11G>C	Benign	not provided
2404	c.5193+11G>A	Pathogenic	not provided
2405	c.5193+10T>G	Benign	not provided
2406	c.5193+10T>A	Benign	not provided
2407	c.5193+9T>G	Pathogenic	not provided
2408	c.5193+9T>C	Benign	not provided
2409	c.5193+9T>A	Benign	not provided
2410	c.5193+8C>T	Benign	not provided
2411	c.5193+8C>G	Benign	not provided
2412	c.5193+7A>T	Benign	not provided
2413	c.5193+7A>G	Pathogenic	not provided
2414	c.5193+7A>C	Benign	not provided
2415	c.5193+6T>G	Benign	not provided
2416	c.5193+6T>A	Benign	not provided
2417	c.5193+5G>T	Benign	not provided
2418	c.5193+5G>C	Benign	not provided
2419	c.5193+5G>A	Pathogenic	not provided
2420	c.5193+4A>G	Benign	not provided
2421	c.5193+4A>C	Benign	not provided
2422	c.5193+4A>T	Pathogenic	not provided
2423	c.5193+3A>T	Benign	not provided
2424	c.5193+3A>C	Benign	not provided
2425	c.5193+2T>C	Benign	not provided
2426	c.5193+2T>A	Pathogenic	not provided
2427	c.5193G>T	Benign	not provided
2428	c.5193G>C	Benign	not provided
2429	c.5192A>T	Pathogenic	not provided

2430	c.5192A>G	Pathogenic	not provided
2431	c.5192A>C	Benign	not provided
2432	c.5191G>C	Benign	not provided
2433	c.5190T>G	Benign	not provided
2434	c.5190T>C	Benign	not provided
2435	c.5190T>A	Benign	not provided
2436	c.5189A>C	Benign	not provided
2437	c.5188A>T	Pathogenic	not provided
2438	c.5188A>G	Benign	not provided
2439	c.5188A>C	Benign	not provided
2440	c.5187G>T	Pathogenic	not provided
2441	c.5187G>C	Benign	not provided
2442	c.5187G>A	Pathogenic	not provided
2443	c.5186T>G	Benign	not provided
2444	c.5185C>G	Benign	not provided
2445	c.5185C>A	Benign	not provided
2446	c.5184G>T	Benign	not provided
2447	c.5184G>C	Benign	not provided
2448	c.5184G>A	Pathogenic	not provided
2449	c.5183T>G	Benign	not provided
2450	c.5183T>C	Benign	not provided
2451	c.5183T>A	Benign	not provided
2452	c.5182A>T	Benign	not provided
2453	c.5182A>G	Benign	not provided
2454	c.5182A>C	Benign	not provided
2455	c.5181A>T	Pathogenic	not provided
2456	c.5181A>G	Benign	not provided
2457	c.5181A>C	Benign	not provided
2458	c.5180A>T	Pathogenic	not provided
2459	c.5180A>G	Benign	not provided
2460	c.5180A>C	Benign	not provided
2461	c.5179A>G	Benign	not provided
2462	c.5178A>T	Pathogenic	not provided
2463	c.5178A>G	Benign	not provided
2464	c.5178A>C	Benign	not provided
2465	c.5177G>A	Pathogenic	not provided
2466	c.5176A>C	Benign	not provided
2467	c.5175A>T	Pathogenic	not provided
2468	c.5174A>T	Pathogenic	not provided
2469	c.5174A>G	Benign	not provided
2470	c.5174A>C	Benign	not provided
2471	c.5173G>C	Benign	not provided
2472	c.5173G>A	Pathogenic	not provided
2473	c.5172A>T	Pathogenic	not provided
2474	c.5172A>G	Benign	not provided
2475	c.5171A>T	Pathogenic	not provided
2476	c.5171A>G	Benign	not provided
2477	c.5171A>C	Benign	not provided
2478	c.5170A>T	Benign	not provided
2479	c.5170A>G	Pathogenic	not provided
2480	c.5170A>C	Benign	not provided
2481	c.5169T>G	Pathogenic	not provided
2482	c.5169T>C	Benign	not provided
2483	c.5169T>A	Benign	not provided

2484	c.5168T>G	Benign	not provided
2485	c.5167A>T	Benign	not provided
2486	c.5167A>C	Benign	not provided
2487	c.5166T>G	Benign	not provided
2488	c.5166T>C	Benign	not provided
2489	c.5166T>A	Benign	not provided
2490	c.5165C>G	Benign	not provided
2491	c.5164T>G	Benign	not provided
2492	c.5164T>A	Benign	not provided
2493	c.5163G>T	Benign	not provided
2494	c.5163G>C	Benign	not provided
2495	c.5162A>T	Pathogenic	not provided
2496	c.5162A>G	Pathogenic	not provided
2497	c.5162A>C	Benign	not provided
2498	c.5161C>A	Pathogenic	not provided
2499	c.5158A>T	Benign	not provided
2500	c.5157G>C	Benign	not provided
2501	c.5156T>A	Benign	not provided
2502	c.5155G>C	Benign	not provided
2503	c.5154G>C	Pathogenic	not provided
2504	c.5153-2A>T	Pathogenic	not provided
2505	c.5153-2A>C	Benign	not provided
2506	c.5153-3T>G	Benign	not provided
2507	c.5153-3T>A	Benign	not provided
2508	c.5153-4T>G	Benign	not provided
2509	c.5153-4T>C	Benign	not provided
2510	c.5153-4T>A	Benign	not provided
2511	c.5153-5T>G	Benign	not provided
2512	c.5153-5T>A	Benign	not provided
2513	c.5153-6C>G	Benign	not provided
2514	c.5153-7T>G	Benign	not provided
2515	c.5153-7T>C	Benign	not provided
2516	c.5153-7T>A	Benign	not provided
2517	c.5153-8C>T	Benign	not provided
2518	c.5153-8C>G	Benign	not provided
2519	c.5153-9T>G	Benign	not provided
2520	c.5153-9T>C	Benign	not provided
2521	c.5153-9T>A	Benign	not provided
2522	c.5153-10A>T	Benign	not provided
2523	c.5153-10A>G	Benign	not provided
2524	c.5153-10A>C	Benign	not provided
2525	c.5153-11G>C	Benign	not provided
2526	c.5153-12T>G	Benign	not provided
2527	c.5153-12T>C	Benign	not provided
2528	c.5153-13A>T	Benign	not provided
2529	c.5153-13A>C	Benign	not provided
2530	c.5153-14T>G	Benign	not provided
2531	c.5153-14T>C	Benign	not provided
2532	c.5153-14T>A	Benign	not provided
2533	c.5153-15C>T	Benign	not provided
2534	c.5153-15C>G	Benign	not provided
2535	c.5153-15C>A	Pathogenic	not provided
2536	c.5153-16T>G	Benign	not provided
2537	c.5153-16T>C	Benign	not provided

2538	c.5153-16T>A	Benign	not provided
2539	c.5153-17T>G	Benign	not provided
2540	c.5153-17T>C	Benign	not provided
2541	c.5153-17T>A	Pathogenic	not provided
2542	c.5153-18T>G	Benign	not provided
2543	c.5153-18T>C	Benign	not provided
2544	c.5153-18T>A	Benign	not provided
2545	c.5153-19T>G	Benign	not provided
2546	c.5153-19T>C	Benign	not provided
2547	c.5153-19T>A	Benign	not provided
2548	c.5153-20C>T	Benign	not provided
2549	c.5153-20C>G	Benign	not provided
2550	c.5153-20C>A	Pathogenic	not provided
2551	c.5153-21T>G	Benign	not provided
2552	c.5153-21T>C	Benign	not provided
2553	c.5153-21T>A	Benign	not provided
2554	c.5153-22G>A	Pathogenic	not provided
2555	c.5153-22G>T	Benign	not provided
2556	c.5153-22G>C	Benign	not provided
2557	c.5153-23T>G	Benign	not provided
2558	c.5153-23T>C	Benign	not provided
2559	c.5153-23T>A	Benign	not provided
2560	c.5153-24C>T	Benign	not provided
2561	c.5153-24C>G	Benign	not provided
2562	c.5153-24C>A	Benign	not provided
2563	c.5153-25C>T	Benign	not provided
2564	c.5153-25C>G	Benign	not provided
2565	c.5153-25C>A	Benign	not provided
2566	c.5153-26A>T	Benign	not provided
2567	c.5153-26A>C	Benign	not provided
2568	c.5153-27A>T	Benign	not provided
2569	c.5153-27A>G	Pathogenic	not provided
2570	c.5153-27A>C	Benign	not provided
2571	c.5153-28T>G	Benign	not provided
2572	c.5153-28T>C	Benign	not provided
2573	c.5153-28T>A	Benign	not provided
2574	c.5153-29G>T	Benign	not provided
2575	c.5153-29G>C	Benign	not provided
2576	c.5153-29G>A	Pathogenic	not provided
2577	c.5153-30T>G	Benign	not provided
2578	c.5153-30T>C	Benign	not provided
2579	c.5153-30T>A	Benign	not provided
2580	c.5152+10A>T	Pathogenic	not provided
2581	c.5152+10A>C	Benign	not provided
2582	c.5152+8T>A	Benign	not provided
2583	c.5152+8T>G	Benign	not provided
2584	c.5152+7A>T	Benign	not provided
2585	c.5152+7A>C	Benign	not provided
2586	c.5152+5G>T	Benign	not provided
2587	c.5152+4A>T	Pathogenic	not provided
2588	c.5152+4A>C	Benign	not provided
2589	c.5152+3A>T	Benign	not provided
2590	c.5152+3A>G	Pathogenic	not provided
2591	c.5152T>C	Benign	not provided

2592	c.5152T>A	Benign	not provided
2593	c.5151C>T	Benign	not provided
2594	c.5151C>G	Benign	not provided
2595	c.5151C>A	Pathogenic	not provided
2596	c.5150T>G	Benign	not provided
2597	c.5150T>C	Benign	not provided
2598	c.5150T>A	Benign	not provided
2599	c.5149T>G	Pathogenic	not provided
2600	c.5149T>C	Benign	not provided
2601	c.5149T>A	Pathogenic	not provided
2602	c.5148T>C	Benign	not provided
2603	c.5147A>T	Benign	not provided
2604	c.5146T>G	Benign	not provided
2605	c.5145C>T	Benign	not provided
2606	c.5144G>C	Pathogenic	not provided
2607	c.5143A>G	Pathogenic	not provided
2608	c.5142T>C	Benign	not provided
2609	c.5142T>A	Benign	not provided
2610	c.5141T>A	Benign	not provided
2611	c.5140G>C	Benign	not provided
2612	c.5140G>A	Benign	not provided
2613	c.5139A>C	Benign	not provided
2614	c.5139A>T	Pathogenic	not provided
2615	c.5138T>G	Pathogenic	not provided
2616	c.5138T>A	Pathogenic	not provided
2617	c.5137G>T	Benign	not provided
2618	c.5137G>C	Pathogenic	not provided
2619	c.5136G>T	Benign	not provided
2620	c.5136G>C	Pathogenic	not provided
2621	c.5135G>T	Benign	not provided
2622	c.5135G>C	Benign	not provided
2623	c.5134T>G	Benign	not provided
2624	c.5134T>C	Benign	not provided
2625	c.5134T>A	Benign	not provided
2626	c.5133A>T	Benign	not provided
2627	c.5133A>C	Benign	not provided
2628	c.5132A>T	Pathogenic	not provided
2629	c.5132A>G	Benign	not provided
2630	c.5132A>C	Benign	not provided
2631	c.5131A>G	Benign	not provided
2632	c.5130A>T	Pathogenic	not provided
2633	c.5130A>G	Benign	not provided
2634	c.5130A>C	Benign	not provided
2635	c.5129G>T	Pathogenic	not provided
2636	c.5129G>C	Benign	not provided
2637	c.5128G>C	Benign	not provided
2638	c.5127A>T	Pathogenic	not provided
2639	c.5127A>G	Pathogenic	not provided
2640	c.5127A>C	Benign	not provided
2641	c.5126G>T	Pathogenic	not provided
2642	c.5126G>C	Benign	not provided
2643	c.5125G>T	Benign	not provided
2644	c.5125G>C	Benign	not provided
2645	c.5124G>T	Benign	not provided

2646	c.5124G>C	Benign	not provided
2647	c.5123C>G	Benign	not provided
2648	c.5122G>T	Benign	not provided
2649	c.5122G>C	Benign	not provided
2650	c.5121T>G	Benign	not provided
2651	c.5121T>C	Benign	not provided
2652	c.5121T>A	Pathogenic	not provided
2653	c.5120T>A	Benign	not provided
2654	c.5119A>T	Benign	not provided
2655	c.5119A>G	Benign	not provided
2656	c.5118A>T	Pathogenic	not provided
2657	c.5118A>G	Benign	not provided
2658	c.5118A>C	Benign	not provided
2659	c.5117G>T	Pathogenic	not provided
2660	c.5116G>T	Pathogenic	not provided
2661	c.5115A>T	Pathogenic	not provided
2662	c.5115A>G	Pathogenic	not provided
2663	c.5114T>A	Benign	not provided
2664	c.5113C>A	Pathogenic	not provided
2665	c.5112T>G	Benign	not provided
2666	c.5112T>C	Benign	not provided
2667	c.5112T>A	Benign	not provided
2668	c.5111T>G	Benign	not provided
2669	c.5111T>C	Benign	not provided
2670	c.5110T>G	Pathogenic	not provided
2671	c.5110T>A	Pathogenic	not provided
2672	c.5109T>A	Benign	not provided
2673	c.5108A>T	Benign	not provided
2674	c.5108A>C	Benign	not provided
2675	c.5107T>A	Benign	not provided
2676	c.5106A>T	Benign	not provided
2677	c.5106A>C	Benign	not provided
2678	c.5105A>T	Pathogenic	not provided
2679	c.5105A>G	Benign	not provided
2680	c.5105A>C	Benign	not provided
2681	c.5104A>T	Pathogenic	not provided
2682	c.5104A>G	Benign	not provided
2683	c.5104A>C	Benign	not provided
2684	c.5103G>T	Pathogenic	not provided
2685	c.5103G>C	Benign	not provided
2686	c.5102T>G	Benign	not provided
2687	c.5102T>A	Benign	not provided
2688	c.5101C>T	Benign	not provided
2689	c.5101C>G	Benign	not provided
2690	c.5100A>T	Benign	not provided
2691	c.5100A>C	Benign	not provided
2692	c.5099C>A	Benign	not provided
2693	c.5099C>T	Benign	not provided
2694	c.5099C>G	Benign	not provided
2695	c.5095C>G	Benign	not provided
2696	c.5094A>T	Benign	not provided
2697	c.5094A>C	Benign	not provided
2698	c.5093A>T	Pathogenic	not provided
2699	c.5093A>G	Benign	not provided

2700	c.5093A>C		Benign	not provided
2701	c.5092G>T		Pathogenic	not provided
2702	c.5092G>C		Benign	not provided
2703	c.5092G>A		Pathogenic	not provided
2704	c.5091T>C		Benign	not provided
2705	c.5091T>A		Benign	not provided
2706	c.5090G>T		Benign	not provided
2707	c.5090G>C		Pathogenic	not provided
2708	c.5089T>G		Benign	not provided
2709	c.5088G>T		Benign	not provided
2710	c.5088G>C		Pathogenic	not provided
2711	c.5087T>G		Benign	not provided
2712	c.5087T>A	rs397509226	Benign	not provided
2713	c.5085T>G		Benign	not provided
2714	c.5085T>C		Benign	not provided
2715	c.5084T>G		Benign	not provided
2716	c.5084T>C		Benign	not provided
2717	c.5084T>A		Pathogenic	not provided
2718	c.5083T>G		Benign	not provided
2719	c.5083T>C		Benign	not provided
2720	c.5083T>A		Benign	not provided
2721	c.5082G>T		Benign	not provided
2722	c.5082G>C		Benign	not provided
2723	c.5082G>A		Benign	not provided
2724	c.5081A>T		Pathogenic	not provided
2725	c.5081A>C		Benign	not provided
2726	c.5080G>C		Benign	not provided
2727	c.5079T>G		Benign	not provided
2728	c.5079T>C		Benign	not provided
2729	c.5079T>A		Benign	not provided
2730	c.5078C>G		Benign	not provided
2731	c.5078C>A		Benign	not provided
2732	c.5077G>T		Benign	not provided
2733	c.5077G>C		Benign	not provided
2734	c.5077G>A		Pathogenic	not provided
2735	c.5076T>G		Benign	not provided
2736	c.5076T>C		Benign	not provided
2737	c.5076T>A		Benign	not provided
2738	c.5075A>G		Benign	not provided
2739	c.5075-3C>A		Benign	not provided
2740	c.5075-4G>C		Benign	not provided
2741	c.5075-5T>G		Benign	not provided
2742	c.5075-5T>C		Benign	not provided
2743	c.5075-5T>A		Benign	not provided
2744	c.5075-6C>G		Benign	not provided
2745	c.5075-7T>G		Pathogenic	not provided
2746	c.5075-7T>A		Benign	not provided
2747	c.5075-8T>C		Benign	not provided
2748	c.5075-8T>A		Benign	not provided
2749	c.5075-10C>T		Benign	not provided
2750	c.5075-10C>G		Benign	not provided
2751	c.5075-10C>A		Benign	not provided
2752	c.5074+10G>T		Pathogenic	not provided
2753	c.5074+10G>C		Benign	not provided

2754	c.5074+10G>A		Pathogenic	not provided
2755	c.5074+9A>T		Pathogenic	not provided
2756	c.5074+9A>G		Benign	not provided
2757	c.5074+9A>C		Benign	not provided
2758	c.5074+8A>T		Pathogenic	not provided
2759	c.5074+8A>G		Benign	not provided
2760	c.5074+8A>C		Benign	not provided
2761	c.5074+7C>G		Benign	not provided
2762	c.5074+7C>A		Benign	not provided
2763	c.5074+6C>T		Benign	not provided
2764	c.5074+6C>A		Pathogenic	not provided
2765	c.5074+5A>G		Pathogenic	not provided
2766	c.5074+5A>C		Benign	not provided
2767	c.5074+4T>G		Benign	not provided
2768	c.5074+3A>T		Benign	not provided
2769	c.5074+3A>C		Benign	not provided
2770	c.5074+2T>G		Pathogenic	not provided
2771	c.5072C>G	rs80357034	Benign	not provided
2772	c.5071A>T		Benign	not provided
2773	c.5071A>C		Benign	not provided
2774	c.5070A>T		Pathogenic	not provided
2775	c.5070A>G		Benign	not provided
2776	c.5070A>C		Benign	not provided
2777	c.5069A>T		Pathogenic	not provided
2778	c.5069A>G		Benign	not provided
2779	c.5069A>C		Benign	not provided
2780	c.5068A>G		Benign	not provided
2781	c.5067G>T		Pathogenic	not provided
2782	c.5067G>C		Benign	not provided
2783	c.5066T>A		Benign	not provided
2784	c.5065A>T		Benign	not provided
2785	c.5064T>G		Benign	not provided
2786	c.5064T>A		Benign	not provided
2787	c.5063T>A		Benign	not provided
2788	c.5063T>G		Benign	not provided
2789	c.5062G>T		Benign	not provided
2790	c.5062G>C		Benign	not provided
2791	c.5062G>A		Pathogenic	not provided
2792	c.5061T>G		Benign	not provided
2793	c.5061T>A		Pathogenic	not provided
2794	c.5060T>C		Benign	not provided
2795	c.5060T>A		Benign	not provided
2796	c.5059G>T		Benign	not provided
2797	c.5059G>C		Benign	not provided
2798	c.5059G>A		Pathogenic	not provided
2799	c.5058T>G		Benign	not provided
2800	c.5058T>A	rs397509218	Benign	not provided
2801	c.5057A>T		Benign	not provided
2802	c.5056C>G		Benign	not provided
2803	c.5056C>A		Benign	not provided
2804	c.5055T>C		Benign	not provided
2805	c.5055T>A		Benign	not provided
2806	c.5054C>G		Benign	not provided
2807	c.5054C>A		Benign	not provided

2808	c.5053A>T	Benign	not provided
2809	c.5053A>C	Benign	not provided
2810	c.5051C>T	Benign	not provided
2811	c.5051C>G	Benign	not provided
2812	c.5051C>A	Benign	not provided
2813	c.5049G>T	Pathogenic	not provided
2814	c.5049G>C	Benign	not provided
2815	c.5049G>A	Pathogenic	not provided
2816	c.5048A>T	Pathogenic	not provided
2817	c.5048A>C	Benign	not provided
2818	c.5047G>C	Benign	not provided
2819	c.5047G>A	Pathogenic	not provided
2820	c.5046A>T	Pathogenic	not provided
2821	c.5046A>G	Benign	not provided
2822	c.5046A>C	Benign	not provided
2823	c.5045A>G	Benign	not provided
2824	c.5045A>C	Benign	not provided
2825	c.5044G>T	Pathogenic	not provided
2826	c.5043T>G	Benign	not provided
2827	c.5043T>C	Benign	not provided
2828	c.5043T>A	Benign	not provided
2829	c.5042C>A	Benign	not provided
2830	c.5042C>G	Benign	not provided
2831	c.5041A>T	Benign	not provided
2832	c.5041A>G	Pathogenic	not provided
2833	c.5040T>G	Pathogenic	not provided
2834	c.5040T>C	Benign	not provided
2835	c.5040T>A	Benign	not provided
2836	c.5039T>G	Benign	not provided
2837	c.5039T>C	Benign	not provided
2838	c.5039T>A	Benign	not provided
2839	c.5038A>T	Pathogenic	not provided
2840	c.5038A>G	Benign	not provided
2841	c.5038A>C	Benign	not provided
2842	c.5037A>T	Pathogenic	not provided
2843	c.5037A>C	Benign	not provided
2844	c.5036T>G	Benign	not provided
2845	c.5036T>A	Benign	not provided
2846	c.5035C>T	Benign	not provided
2847	c.5035C>A	Pathogenic	not provided
2848	c.5034T>G	Benign	not provided
2849	c.5034T>A	Benign	not provided
2850	c.5033A>T	Pathogenic	not provided
2851	c.5033A>G	Benign	not provided
2852	c.5033A>C	Benign	not provided
2853	c.5032A>T	Pathogenic	not provided
2854	c.5032A>G	Pathogenic	not provided
2855	c.5032A>C	Benign	not provided
2856	c.5031T>C	Benign	not provided
2857	c.5031T>G	Benign	not provided
2858	c.5030C>G	Benign	not provided
2859	c.5030C>A	Benign	not provided
2860	c.5029A>T	Benign	not provided
2861	c.5029A>G	Benign	not provided

2862	c.5029A>C	Benign	not provided
2863	c.5028A>C	Benign	not provided
2864	c.5026T>G	Benign	not provided
2865	c.5026T>A	Benign	not provided
2866	c.5024C>A	Benign	not provided
2867	c.5023A>C	Benign	not provided
2868	c.5021T>C	Benign	not provided
2869	c.5020A>G	Benign	not provided
2870	c.5020A>C	Benign	not provided
2871	c.5019C>T	Benign	not provided
2872	c.5019C>G	Benign	not provided
2873	c.5019C>A	Benign	not provided
2874	c.5018A>T	Benign	not provided
2875	c.5018A>C	Benign	not provided
2876	c.5017C>A	Pathogenic	not provided
2877	c.5017C>T	Benign	not provided
2878	c.5017C>G	Benign	not provided
2879	c.5016C>T	Benign	not provided
2880	c.5016C>G	Benign	not provided
2881	c.5016C>A	Pathogenic	not provided
2882	c.5015A>G	Benign	not provided
2883	c.5015A>C	Benign	not provided
2884	c.5014C>G	Benign	not provided
2885	c.5014C>A	Benign	not provided
2886	c.5013A>T	Benign	not provided
2887	c.5013A>G	Benign	not provided
2888	c.5013A>C	Benign	not provided
2889	c.5012A>T	Pathogenic	not provided
2890	c.5012A>G	Benign	not provided
2891	c.5012A>C	Benign	not provided
2892	c.5011A>T	Pathogenic	not provided
2893	c.5011A>G	Benign	not provided
2894	c.5011A>C	Benign	not provided
2895	c.5010A>T	Pathogenic	not provided
2896	c.5010A>C	Benign	not provided
2897	c.5009G>T	Pathogenic	not provided
2898	c.5009G>C	Benign	not provided
2899	c.5009G>A	Pathogenic	not provided
2900	c.5008A>T	Pathogenic	not provided
2901	c.5008A>G	Pathogenic	not provided
2902	c.5007C>G	Benign	not provided
2903	c.5007C>A	Pathogenic	not provided
2904	c.5006C>A	Benign	not provided
2905	c.5006C>G	Benign	not provided
2906	c.5005G>C	Benign	not provided
2907	c.5005G>A	Pathogenic	not provided
2908	c.5004T>G	Benign	not provided
2909	c.5004T>C	Benign	not provided
2910	c.5004T>A	Pathogenic	not provided
2911	c.5003T>A	Pathogenic	not provided
2912	c.5002T>G	Benign	not provided
2913	c.5002T>C	Benign	not provided
2914	c.5001G>T	Benign	not provided
2915	c.5001G>C	Benign	not provided

2916	c.5000A>T		Pathogenic	not provided
2917	c.5000A>C		Benign	not provided
2918	c.4999A>C		Benign	not provided
2919	c.4998C>G		Benign	not provided
2920	c.4997A>T		Benign	not provided
2921	c.4997A>C		Benign	not provided
2922	c.4996T>G		Benign	not provided
2923	c.4996T>A		Benign	not provided
2924	c.4996T>C	rs397509215	Benign	not provided
2925	c.4995G>C		Benign	not provided
2926	c.4995G>A		Pathogenic	not provided
2927	c.4995G>T		Benign	not provided
2928	c.4994T>C		Benign	not provided
2929	c.4994T>A		Benign	not provided
2930	c.4992C>G		Benign	not provided
2931	c.4991T>G		Benign	not provided
2932	c.4991T>A		Pathogenic	not provided
2933	c.4990C>T		Benign	not provided
2934	c.4990C>G		Benign	not provided
2935	c.4990C>A		Benign	not provided
2936	c.4989G>T		Benign	not provided
2937	c.4989G>C		Benign	not provided
2938	c.4989G>A		Pathogenic	not provided
2939	c.4988T>G		Benign	not provided
2940	c.4988T>C		Benign	not provided
2941	c.4987A>G		Benign	not provided
2942	c.4987A>C		Benign	not provided
2943	c.4987-2A>T		Pathogenic	not provided
2944	c.4987-3C>A		Pathogenic	not provided
2945	c.4987-4T>C		Benign	not provided
2946	c.4987-4T>A		Benign	not provided
2947	c.4987-5T>G		Pathogenic	not provided
2948	c.4987-6T>G		Benign	not provided
2949	c.4987-6T>C		Benign	not provided
2950	c.4987-6T>A		Benign	not provided
2951	c.4987-7A>T		Pathogenic	not provided
2952	c.4987-7A>G		Benign	not provided
2953	c.4987-7A>C		Benign	not provided
2954	c.4987-8A>T		Pathogenic	not provided
2955	c.4987-8A>C		Benign	not provided
2956	c.4987-9T>G		Benign	not provided
2957	c.4987-9T>C		Benign	not provided
2958	c.4987-9T>A		Benign	not provided
2959	c.4987-10T>C		Benign	not provided
2960	c.4987-10T>A		Pathogenic	not provided
2961	c.4986+10T>G		Benign	not provided
2962	c.4986+10T>C		Benign	not provided
2963	c.4986+10T>A		Benign	not provided
2964	c.4986+9A>G		Pathogenic	not provided
2965	c.4986+9A>C		Benign	not provided
2966	c.4986+8T>G		Benign	not provided
2967	c.4986+8T>C		Benign	not provided
2968	c.4986+8T>A		Benign	not provided
2969	c.4986+7G>T		Benign	not provided

2970	c.4986+7G>C	Benign	not provided
2971	c.4986+7G>A	Pathogenic	not provided
2972	c.4986+3G>T	Pathogenic	not provided
2973	c.4986+3G>A	Pathogenic	not provided
2974	c.4986+2T>G	Benign	not provided
2975	c.4986+2T>A	Benign	not provided
2976	c.4986+1G>C	Benign	not provided
2977	c.4986T>G	Benign	not provided
2978	c.4986T>C	Benign	not provided
2979	c.4986T>A	Benign	not provided
2980	c.4985T>G	Benign	not provided
2981	c.4985T>A	Pathogenic	not provided
2982	c.4984T>G	Benign	not provided
2983	c.4984T>C	Benign	not provided
2984	c.4984T>A	Benign	not provided
2985	c.4983A>T	Benign	not provided
2986	c.4983A>G	Benign	not provided
2987	c.4983A>C	Benign	not provided
2988	c.4982A>T	Pathogenic	not provided
2989	c.4982A>G	Benign	not provided
2990	c.4982A>C	Benign	not provided
2991	c.4981G>C	Benign	not provided
2992	c.4980A>T	Pathogenic	not provided
2993	c.4980A>G	Benign	not provided
2994	c.4980A>C	Benign	not provided
2995	c.4979A>T	Pathogenic	not provided
2996	c.4979A>G	Benign	not provided
2997	c.4979A>C	Benign	not provided
2998	c.4978G>T	Pathogenic	not provided
2999	c.4978G>C	Benign	not provided
3000	c.4978G>A	Pathogenic	not provided
3001	c.4977A>T	Pathogenic	not provided
3002	c.4977A>G	Pathogenic	not provided
3003	c.4976C>T	Benign	not provided
3004	c.4976C>G	Benign	not provided
3005	c.4976C>A	Pathogenic	not provided
3006	c.4975C>T	Benign	not provided
3007	c.4975C>G	Benign	not provided
3008	c.4975C>A	Pathogenic	not provided
3009	c.4972A>T	Benign	not provided
3010	c.4972A>G	Benign	not provided
3011	c.4971G>T	Pathogenic	not provided
3012	c.4970T>G	Benign	not provided
3013	c.4970T>A	Benign	not provided
3014	c.4969C>T	Benign	not provided
3015	c.4969C>G	Benign	not provided
3016	c.4969C>A	Benign	not provided
3017	c.4968C>T	Benign	not provided
3018	c.4968C>G	Benign	not provided
3019	c.4968C>A	Benign	not provided
3020	c.4967G>T	Benign	not provided
3021	c.4967G>C	Benign	not provided
3022	c.4966G>T	Benign	not provided
3023	c.4966G>C	Benign	not provided

3024	c.4966G>A	Pathogenic	not provided
3025	c.4965T>G	Benign	not provided
3026	c.4965T>C	Benign	not provided
3027	c.4965T>A	Benign	not provided
3028	c.4964C>G	Benign	not provided
3029	c.4964C>A	Pathogenic	not provided
3030	c.4963T>G	Benign	not provided
3031	c.4963T>A	Benign	not provided
3032	c.4962G>T	Benign	not provided
3033	c.4962G>C	Benign	not provided
3034	c.4961T>G	Benign	not provided
3035	c.4961T>C	Benign	not provided
3036	c.4961T>A	Benign	not provided
3037	c.4960G>T	Benign	not provided
3038	c.4960G>C	Benign	not provided
3039	c.4960G>A	Benign	not provided
3040	c.4959G>T	Benign	not provided
3041	c.4959G>C	Benign	not provided
3042	c.4958T>G	Benign	not provided
3043	c.4958T>C	Benign	not provided
3044	c.4958T>A	Benign	not provided
3045	c.4957G>T	Benign	not provided
3046	c.4957G>C	Benign	not provided
3047	c.4956G>T	Benign	not provided
3048	c.4956G>C	Benign	not provided
3049	c.4955T>G	Benign	not provided
3050	c.4954A>C	Benign	not provided
3051	c.4953C>T	Benign	not provided
3052	c.4953C>G	Benign	not provided
3053	c.4953C>A	Benign	not provided
3054	c.4952C>G	Benign	not provided
3055	c.4952C>A	Benign	not provided
3056	c.4951T>G	Benign	not provided
3057	c.4951T>A	Benign	not provided
3058	c.4950G>T	Benign	not provided
3059	c.4950G>C	Benign	not provided
3060	c.4949T>G	Benign	not provided
3061	c.4949T>A	Benign	not provided
3062	c.4948A>C	Benign	not provided
3063	c.4948A>T	Benign	not provided
3064	c.4948A>G	Benign	not provided
3065	c.4947A>T	Pathogenic	not provided
3066	c.4947A>G	Benign	not provided
3067	c.4947A>C	Benign	not provided
3068	c.4946G>T	Pathogenic	not provided
3069	c.4946G>A	Pathogenic	not provided
3070	c.4945A>T	Pathogenic	not provided
3071	c.4945A>G	Benign	not provided
3072	c.4945A>C	Benign	not provided
3073	c.4944A>T	Pathogenic	not provided
3074	c.4944A>G	Benign	not provided
3075	c.4944A>C	Benign	not provided
3076	c.4943A>T	Pathogenic	not provided
3077	c.4943A>G	Benign	not provided

3078	c.4943A>C	Benign	not provided
3079	c.4942A>C	Benign	not provided
3080	c.4941C>T	Benign	not provided
3081	c.4941C>G	Benign	not provided
3082	c.4940A>T	Benign	not provided
3083	c.4940A>G	Benign	not provided
3084	c.4940A>C	Benign	not provided
3085	c.4939A>T	Pathogenic	not provided
3086	c.4939A>G	Pathogenic	not provided
3087	c.4939A>C	Benign	not provided
3088	c.4938C>T	Benign	not provided
3089	c.4938C>G	Pathogenic	not provided
3090	c.4938C>A	Pathogenic	not provided
3091	c.4937T>C	Benign	not provided
3092	c.4937T>A	Benign	not provided
3093	c.4937T>G	Benign	not provided
3094	c.4936G>T	Benign	not provided
3095	c.4936G>C	Benign	not provided
3096	c.4936G>A	Pathogenic	not provided
3097	c.4935G>T	Benign	not provided
3098	c.4935G>A	Pathogenic	not provided
3099	c.4934G>A	Pathogenic	not provided
3100	c.4933A>T	Pathogenic	not provided
3101	c.4933A>C	Benign	not provided
3102	c.4932A>T	Pathogenic	not provided
3103	c.4932A>G	Benign	not provided
3104	c.4932A>C	Benign	not provided
3105	c.4931A>T	Pathogenic	not provided
3106	c.4931A>C	Benign	not provided
3107	c.4930G>A	Pathogenic	not provided
3108	c.4929A>T	Pathogenic	not provided
3109	c.4929A>G	Pathogenic	not provided
3110	c.4928C>T	Benign	not provided
3111	c.4928C>G	Benign	not provided
3112	c.4928C>A	Benign	not provided
3113	c.4927A>T	Benign	not provided
3114	c.4927A>G	Benign	not provided
3115	c.4927A>C	Benign	not provided
3116	c.4926A>T	Pathogenic	not provided
3117	c.4926A>G	Pathogenic	not provided
3118	c.4926A>C	Benign	not provided
3119	c.4925C>T	Benign	not provided
3120	c.4925C>G	Pathogenic	not provided
3121	c.4925C>A	Pathogenic	not provided
3122	c.4924T>G	Benign	not provided
3123	c.4924T>C	Benign	not provided
3124	c.4924T>A	Benign	not provided
3125	c.4923T>C	Benign	not provided
3126	c.4923T>A	Pathogenic	not provided
3127	c.4922C>G	Benign	not provided
3128	c.4922C>A	Benign	not provided
3129	c.4921G>T	Benign	not provided
3130	c.4921G>C	Benign	not provided
3131	c.4920A>T	Pathogenic	not provided

3132	c.4920A>G	Pathogenic	not provided
3133	c.4920A>C	Benign	not provided
3134	c.4919C>T	Benign	not provided
3135	c.4919C>G	Benign	not provided
3136	c.4919C>A	Benign	not provided
3137	c.4918A>T	Benign	not provided
3138	c.4918A>C	Benign	not provided
3139	c.4917G>T	Pathogenic	not provided
3140	c.4917G>C	Benign	not provided
3141	c.4916T>A	Pathogenic	not provided
3142	c.4916T>G	Benign	not provided
3143	c.4915T>G	Benign	not provided
3144	c.4915T>A	Benign	not provided
3145	c.4914A>T	Benign	not provided
3146	c.4914A>C	Benign	not provided
3147	c.4913A>T	Pathogenic	not provided
3148	c.4913A>G	Benign	not provided
3149	c.4913A>C	Benign	not provided
3150	c.4912G>T	Pathogenic	not provided
3151	c.4912G>C	Benign	not provided
3152	c.4912G>A	Pathogenic	not provided
3153	c.4911A>T	Pathogenic	not provided
3154	c.4911A>G	Pathogenic	not provided
3155	c.4911A>C	Benign	not provided
3156	c.4910C>G	Benign	not provided
3157	c.4909C>G	Benign	not provided
3158	c.4909C>A	Benign	not provided
3159	c.4908G>T	Benign	not provided
3160	c.4908G>C	Benign	not provided
3161	c.4907A>T	Pathogenic	not provided
3162	c.4907A>G	Benign	not provided
3163	c.4907A>C	Benign	not provided
3164	c.4906A>T	Pathogenic	not provided
3165	c.4906A>G	Benign	not provided
3166	c.4906A>C	Benign	not provided
3167	c.4905G>C	Benign	not provided
3168	c.4905G>A	Pathogenic	not provided
3169	c.4905G>T	Pathogenic	not provided
3170	c.4904A>T	Pathogenic	not provided
3171	c.4904A>G	Pathogenic	not provided
3172	c.4904A>C	Benign	not provided
3173	c.4902G>T	Benign	not provided
3174	c.4902G>C	Benign	not provided
3175	c.4901G>C	Benign	not provided
3176	c.4901G>A	Pathogenic	not provided
3177	c.4900A>C	Benign	not provided
3178	c.4899C>G	Benign	not provided
3179	c.4898G>T	Benign	not provided
3180	c.4898G>C	Benign	not provided
3181	c.4898G>A	Pathogenic	not provided
3182	c.4897A>T	Pathogenic	not provided
3183	c.4897A>G	Pathogenic	not provided
3184	c.4896G>T	Pathogenic	not provided
3185	c.4896G>C	Benign	not provided

3186	c.4896G>A		Pathogenic	not provided
3187	c.4895T>C		Benign	not provided
3188	c.4895T>A		Benign	not provided
3189	c.4893T>G		Benign	not provided
3190	c.4892G>T		Benign	not provided
3191	c.4892G>C		Benign	not provided
3192	c.4891A>G		Benign	not provided
3193	c.4891A>C		Benign	not provided
3194	c.4777A>T	rs397509197	Benign	not provided
3195	c.4743A>C	rs397509194	Benign	not provided
3196	c.4471C>G	rs111034213	Benign	not provided
3197	c.4343G>C	rs80357354	Benign	not provided
3198	c.4172T>C	rs397509146	Benign	not provided
3199	c.3829G>C	rs397509112	Benign	not provided
3200	c.3758C>A	rs397509100	Pathogenic	not provided
3201	c.3722C>G	rs80357143	Benign	not provided
3202	c.3613G>C	rs80357294	Benign	not provided
3203	c.3228A>T	rs397509048	Pathogenic	not provided
3204	c.3152C>G	rs397509039	Benign	not provided
3205	c.3140T>C	rs397509037	Benign	not provided
3206	c.3074C>T	rs397509034	Benign	not provided
3207	c.3072C>G	rs397509033	Benign	not provided
3208	c.2879G>A	rs397509022	Benign	not provided
3209	c.2675T>C	rs397508994	Benign	not provided
3210	c.2600A>G	rs397508985	Pathogenic	not provided
3211	c.2413T>C	rs397508966	Benign	not provided
3212	c.2330A>G	rs587778116	Pathogenic	not provided
3213	c.1960A>G	rs80357355	Benign	not provided
3214	c.1932T>G	rs397508918	Benign	not provided
3215	c.1744A>G	rs397508906	Benign	not provided
3216	c.1690A>C	rs397507191	Benign	not provided
3217	c.1669A>G	rs397508895	Benign	not provided
3218	c.1655G>T	rs397508893	Benign	not provided
3219	c.1544A>G	rs397508881	Pathogenic	not provided
3220	c.1393T>G	rs397508869	Benign	not provided
3221	c.1262A>G	rs397508849	Benign	not provided
3222	c.1076C>T	rs397508831	Benign	not provided
3223	c.791G>A	rs397509322	Pathogenic	not provided
3224	c.790A>T	rs397509321	Pathogenic	not provided
3225	c.788G>T	rs397509319	Benign	not provided
3226	c.788G>C	rs397509319	Pathogenic	not provided
3227	c.787G>T	rs397509318	Benign	not provided
3228	c.668A>G	rs397509306	Benign	not provided
3229	c.472A>T	rs397509192	Benign	not provided
3230	c.466C>G	rs587778115	Benign	not provided
3231	c.346G>A	rs397509071	Pathogenic	not provided
3232	c.340T>C	rs397509062	Benign	not provided
3233	c.329A>C	rs397509053	Benign	not provided
3234	c.301+10G>T		Pathogenic	not provided
3235	c.301+10G>C		Benign	not provided
3236	c.301+9T>G		Benign	not provided
3237	c.301+8T>G		Benign	not provided
3238	c.301+8T>A		Benign	not provided
3239	c.301+7G>T		Benign	not provided

3240	c.301+7G>C	Benign	not provided
3241	c.301+6T>G	Benign	not provided
3242	c.301+6T>A	Benign	not provided
3243	c.301+5G>C	Benign	not provided
3244	c.301+4A>T	Pathogenic	not provided
3245	c.301+4A>G	Benign	not provided
3246	c.301+4A>C	Benign	not provided
3247	c.301+3A>T	Pathogenic	not provided
3248	c.301+2T>A	Benign	not provided
3249	c.301+2T>G	Benign	not provided
3250	c.301T>G	Benign	not provided
3251	c.301T>A	Benign	not provided
3252	c.300G>T	Benign	not provided
3253	c.300G>C	Benign	not provided
3254	c.298G>T	Pathogenic	not provided
3255	c.295T>G	Benign	not provided
3256	c.295T>C	Benign	not provided
3257	c.294T>G	Benign	not provided
3258	c.294T>C	Benign	not provided
3259	c.294T>A	Benign	not provided
3260	c.293G>T	Benign	not provided
3261	c.293G>C	Benign	not provided
3262	c.293G>A	Benign	not provided
3263	c.292G>T	Benign	not provided
3264	c.292G>A	Pathogenic	not provided
3265	c.291A>T	Pathogenic	not provided
3266	c.290C>A	Benign	not provided
3267	c.289A>T	Benign	not provided
3268	c.289A>C	Benign	not provided
3269	c.288C>A	Benign	not provided
3270	c.288C>G	Benign	not provided
3271	c.287A>T	Benign	not provided
3272	c.287A>C	Benign	not provided
3273	c.286G>T	Pathogenic	not provided
3274	c.285T>G	Benign	not provided
3275	c.285T>C	Benign	not provided
3276	c.285T>A	Benign	not provided
3277	c.284T>G	Benign	not provided
3278	c.284T>C	Benign	not provided
3279	c.284T>A	Benign	not provided
3280	c.283C>T	Benign	not provided
3281	c.283C>G	Benign	not provided
3282	c.283C>A	Benign	not provided
3283	c.282G>C	Benign	not provided
3284	c.282G>A	Benign	not provided
3285	c.281A>T	Pathogenic	not provided
3286	c.281A>G	Pathogenic	not provided
3287	c.281A>C	Benign	not provided
3288	c.280C>G	Pathogenic	not provided
3289	c.280C>A	Pathogenic	not provided
3290	c.279T>G	Benign	not provided
3291	c.279T>C	Benign	not provided
3292	c.279T>A	Benign	not provided
3293	c.278T>G	Benign	not provided

3294	c.278T>C		Benign	not provided
3295	c.278T>A		Pathogenic	not provided
3296	c.277T>C		Benign	not provided
3297	c.277T>A		Benign	not provided
3298	c.277T>G		Benign	not provided
3299	c.276T>G		Benign	not provided
3300	c.276T>C		Benign	not provided
3301	c.276T>A		Benign	not provided
3302	c.275C>T		Benign	not provided
3303	c.275C>A		Benign	not provided
3304	c.274G>T		Benign	not provided
3305	c.274G>A		Pathogenic	not provided
3306	c.273T>C		Benign	not provided
3307	c.273T>A		Benign	not provided
3308	c.273T>G	rs397509006	Benign	not provided
3309	c.272G>T		Benign	not provided
3310	c.271T>G		Benign	not provided
3311	c.271T>A		Benign	not provided
3312	c.270T>G		Pathogenic	not provided
3313	c.270T>A		Pathogenic	not provided
3314	c.269T>G		Benign	not provided
3315	c.269T>A		Benign	not provided
3316	c.268A>T		Benign	not provided
3317	c.268A>C		Benign	not provided
3318	c.267C>T		Benign	not provided
3319	c.267C>A		Pathogenic	not provided
3320	c.266T>G		Benign	not provided
3321	c.266T>A		Benign	not provided
3322	c.265A>T		Benign	not provided
3323	c.265A>G		Benign	not provided
3324	c.265A>C		Benign	not provided
3325	c.264A>T		Pathogenic	not provided
3326	c.264A>G		Benign	not provided
3327	c.264A>C		Benign	not provided
3328	c.263A>G		Benign	not provided
3329	c.263A>C		Benign	not provided
3330	c.262A>T		Pathogenic	not provided
3331	c.262A>G		Benign	not provided
3332	c.262A>C		Benign	not provided
3333	c.261G>T		Pathogenic	not provided
3334	c.261G>C		Benign	not provided
3335	c.260T>C		Benign	not provided
3336	c.259T>A		Benign	not provided
3337	c.258A>C		Benign	not provided
3338	c.257T>G		Benign	not provided
3339	c.257T>C		Benign	not provided
3340	c.257T>A		Benign	not provided
3341	c.256C>T		Benign	not provided
3342	c.256C>G		Benign	not provided
3343	c.256C>A		Benign	not provided
3344	c.255G>C		Benign	not provided
3345	c.254A>T		Pathogenic	not provided
3346	c.254A>G		Pathogenic	not provided
3347	c.254A>C		Benign	not provided

3348	c.253G>T	Pathogenic	not provided
3349	c.253G>C	Benign	not provided
3350	c.253G>A	Pathogenic	not provided
3351	c.252A>T	Pathogenic	not provided
3352	c.252A>G	Benign	not provided
3353	c.252A>C	Benign	not provided
3354	c.251A>T	Pathogenic	not provided
3355	c.251A>G	Benign	not provided
3356	c.251A>C	Benign	not provided
3357	c.250G>C	Benign	not provided
3358	c.250G>A	Pathogenic	not provided
3359	c.249T>G	Benign	not provided
3360	c.249T>A	Pathogenic	not provided
3361	c.248T>G	Benign	not provided
3362	c.248T>A	Benign	not provided
3363	c.247G>C	Benign	not provided
3364	c.246T>G	Benign	not provided
3365	c.246T>C	Benign	not provided
3366	c.246T>A	Benign	not provided
3367	c.245T>G	Benign	not provided
3368	c.245T>C	Benign	not provided
3369	c.245T>A	Benign	not provided
3370	c.244C>T	Benign	not provided
3371	c.244C>G	Benign	not provided
3372	c.244C>A	Benign	not provided
3373	c.243A>T	Benign	not provided
3374	c.243A>C	Benign	not provided
3375	c.242A>G	Pathogenic	not provided
3376	c.242A>C	Benign	not provided
3377	c.241C>A	Pathogenic	not provided
3378	c.240T>G	Benign	not provided
3379	c.240T>C	Benign	not provided
3380	c.240T>A	Benign	not provided
3381	c.239G>T	Benign	not provided
3382	c.239G>C	Benign	not provided
3383	c.239G>A	Pathogenic	not provided
3384	c.238A>T	Pathogenic	not provided
3385	c.238A>G	Pathogenic	not provided
3386	c.238A>C	Benign	not provided
3387	c.237T>G	Benign	not provided
3388	c.237T>C	Benign	not provided
3389	c.237T>A	Benign	not provided
3390	c.236T>G	Pathogenic	not provided
3391	c.236T>C	Benign	not provided
3392	c.236T>A	Pathogenic	not provided
3393	c.235T>C	Benign	not provided
3394	c.234A>T	Pathogenic	not provided
3395	c.234A>G	Benign	not provided
3396	c.234A>C	Benign	not provided
3397	c.233G>T	Pathogenic	not provided
3398	c.233G>C	Benign	not provided
3399	c.233G>A	Pathogenic	not provided
3400	c.232A>T	Pathogenic	not provided
3401	c.232A>G	Pathogenic	not provided

3402	c.232A>C	Benign	not provided
3403	c.230C>A	Benign	not provided
3404	c.229A>T	Benign	not provided
3405	c.229A>G	Pathogenic	not provided
3406	c.229A>C	Benign	not provided
3407	c.228T>C	Benign	not provided
3408	c.228T>A	Benign	not provided
3409	c.227G>T	Benign	not provided
3410	c.227G>C	Benign	not provided
3411	c.227G>A	Benign	not provided
3412	c.226A>T	Pathogenic	not provided
3413	c.226A>G	Benign	not provided
3414	c.226A>C	Benign	not provided
3415	c.225A>T	Pathogenic	not provided
3416	c.225A>G	Benign	not provided
3417	c.225A>C	Benign	not provided
3418	c.224A>G	Benign	not provided
3419	c.224A>C	Benign	not provided
3420	c.223G>T	Pathogenic	not provided
3421	c.223G>C	Benign	not provided
3422	c.223G>A	Pathogenic	not provided
3423	c.222A>T	Pathogenic	not provided
3424	c.221A>T	Pathogenic	not provided
3425	c.221A>G	Benign	not provided
3426	c.221A>C	Benign	not provided
3427	c.220C>G	Benign	not provided
3428	c.219A>T	Benign	not provided
3429	c.219A>C	Benign	not provided
3430	c.218T>G	Benign	not provided
3431	c.218T>C	Benign	not provided
3432	c.218T>A	Benign	not provided
3433	c.217C>G	Benign	not provided
3434	c.215G>T	Benign	not provided
3435	c.215G>C	Benign	not provided
3436	c.215G>A	Benign	not provided
3437	c.214A>T	Pathogenic	not provided
3438	c.214A>G	Pathogenic	not provided
3439	c.214A>C	Benign	not provided
3440	c.213G>T	Pathogenic	not provided
3441	c.213G>C	Benign	not provided
3442	c.213G>A	Pathogenic	not provided
3443	c.213-1G>C	Benign	not provided
3444	c.213-3C>T	Benign	not provided
3445	c.213-3C>A	Pathogenic	not provided
3446	c.213-4T>G	Benign	not provided
3447	c.213-4T>C	Benign	not provided
3448	c.213-4T>A	Benign	not provided
3449	c.213-5T>G	Benign	not provided
3450	c.213-5T>C	Benign	not provided
3451	c.213-6T>G	Pathogenic	not provided
3452	c.213-6T>C	Benign	not provided
3453	c.213-6T>A	Benign	not provided
3454	c.213-7A>T	Pathogenic	not provided
3455	c.213-7A>G	Benign	not provided

3456	c.213-7A>C		Benign	not provided
3457	c.213-8A>G		Pathogenic	not provided
3458	c.213-8A>T		Pathogenic	not provided
3459	c.213-9T>G		Benign	not provided
3460	c.213-9T>C		Benign	not provided
3461	c.213-9T>A		Benign	not provided
3462	c.213-10T>G		Benign	not provided
3463	c.213-10T>C		Benign	not provided
3464	c.213-10T>A		Pathogenic	not provided
3465	c.212+10T>A		Pathogenic	not provided
3466	c.212+10T>C	rs80358174	Benign	not provided
3467	c.212+9T>G		Benign	not provided
3468	c.212+9T>C		Benign	not provided
3469	c.212+9T>A		Benign	not provided
3470	c.212+8A>C		Benign	not provided
3471	c.212+8A>T		Pathogenic	not provided
3472	c.212+8A>G		Benign	not provided
3473	c.212+7A>T		Pathogenic	not provided
3474	c.212+7A>G		Pathogenic	not provided
3475	c.212+7A>C		Benign	not provided
3476	c.212+6T>G		Benign	not provided
3477	c.212+6T>C		Benign	not provided
3478	c.212+6T>A		Benign	not provided
3479	c.212+5A>T		Benign	not provided
3480	c.212+5A>G		Pathogenic	not provided
3481	c.212+5A>C		Benign	not provided
3482	c.212+3A>C		Benign	not provided
3483	c.212+2T>G		Benign	not provided
3484	c.212+2T>A		Pathogenic	not provided
3485	c.211A>T		Pathogenic	not provided
3486	c.211A>C		Benign	not provided
3487	c.210A>T		Pathogenic	not provided
3488	c.210A>G		Benign	not provided
3489	c.210A>C		Benign	not provided
3490	c.209A>G		Benign	not provided
3491	c.209A>C		Benign	not provided
3492	c.209A>T		Pathogenic	not provided
3493	c.208A>T		Pathogenic	not provided
3494	c.208A>G		Benign	not provided
3495	c.208A>C		Benign	not provided
3496	c.207C>A		Pathogenic	not provided
3497	c.206C>G		Benign	not provided
3498	c.205A>T		Benign	not provided
3499	c.205A>G		Benign	not provided
3500	c.205A>C		Benign	not provided
3501	c.204A>G		Pathogenic	not provided
3502	c.204A>C		Benign	not provided
3503	c.203T>C		Benign	not provided
3504	c.202A>T		Benign	not provided
3505	c.202A>C		Benign	not provided
3506	c.201T>A		Benign	not provided
3507	c.200A>G		Benign	not provided
3508	c.200A>C		Benign	not provided
3509	c.199G>C		Benign	not provided

3510	c.198T>G		Benign	not provided
3511	c.197A>T		Pathogenic	not provided
3512	c.197A>G		Benign	not provided
3513	c.197A>C		Benign	not provided
3514	c.196A>C		Benign	not provided
3515	c.196A>T		Pathogenic	not provided
3516	c.196A>G		Benign	not provided
3517	c.195G>T		Pathogenic	not provided
3518	c.195G>C		Benign	not provided
3519	c.195G>A		Pathogenic	not provided
3520	c.194A>T		Pathogenic	not provided
3521	c.194A>G		Benign	not provided
3522	c.194A>C		Benign	not provided
3523	c.193A>T		Pathogenic	not provided
3524	c.193A>C		Benign	not provided
3525	c.192T>C		Benign	not provided
3526	c.191G>T		Benign	not provided
3527	c.190T>A		Benign	not provided
3528	c.189A>G		Pathogenic	not provided
3529	c.189A>C		Benign	not provided
3530	c.188T>G		Benign	not provided
3531	c.188T>C	rs80357086	Benign	not provided
3532	c.187T>G		Benign	not provided
3533	c.187T>C		Benign	not provided
3534	c.187T>A		Benign	not provided
3535	c.186T>G		Benign	not provided
3536	c.186T>C		Benign	not provided
3537	c.186T>A		Benign	not provided
3538	c.185C>A		Benign	not provided
3539	c.185C>T		Benign	not provided
3540	c.184C>T		Benign	not provided
3541	c.184C>G		Pathogenic	not provided
3542	c.184C>A		Benign	not provided
3543	c.183T>G		Benign	not provided
3544	c.183T>A		Benign	not provided
3545	c.182G>T		Benign	not provided
3546	c.182G>C		Benign	not provided
3547	c.180G>T		Benign	not provided
3548	c.180G>C		Benign	not provided
3549	c.180G>A		Pathogenic	not provided
3550	c.179A>T		Pathogenic	not provided
3551	c.179A>C		Benign	not provided
3552	c.178C>G		Benign	not provided
3553	c.178C>A		Pathogenic	not provided
3554	c.177A>T		Benign	not provided
3555	c.177A>G		Pathogenic	not provided
3556	c.177A>C		Benign	not provided
3557	c.176C>G		Benign	not provided
3558	c.175T>G		Benign	not provided
3559	c.175T>C		Benign	not provided
3560	c.175T>A		Benign	not provided
3561	c.174T>G		Benign	not provided
3562	c.174T>C		Benign	not provided
3563	c.174T>A		Benign	not provided

3564	c.173C>T		Benign	not provided
3565	c.173C>G		Benign	not provided
3566	c.173C>A		Benign	not provided
3567	c.170G>T		Benign	not provided
3568	c.168A>G		Benign	not provided
3569	c.168A>C		Benign	not provided
3570	c.168A>T	rs397508898	Pathogenic	not provided
3571	c.167A>T		Pathogenic	not provided
3572	c.167A>C		Benign	not provided
3573	c.167A>G	rs397508897	Benign	not provided
3574	c.166A>T		Pathogenic	not provided
3575	c.166A>G		Benign	not provided
3576	c.166A>C		Benign	not provided
3577	c.165G>T		Pathogenic	not provided
3578	c.165G>C		Benign	not provided
3579	c.164A>T		Pathogenic	not provided
3580	c.164A>C		Benign	not provided
3581	c.164A>G		Benign	not provided
3582	c.163A>T		Pathogenic	not provided
3583	c.163A>G		Benign	not provided
3584	c.163A>C		Benign	not provided
3585	c.162G>T		Pathogenic	not provided
3586	c.162G>C		Benign	not provided
3587	c.162G>A		Pathogenic	not provided
3588	c.161A>T		Pathogenic	not provided
3589	c.161A>C		Benign	not provided
3590	c.160C>G		Benign	not provided
3591	c.159C>G		Benign	not provided
3592	c.159C>A		Pathogenic	not provided
3593	c.158A>T		Benign	not provided
3594	c.158A>G		Benign	not provided
3595	c.158A>C		Benign	not provided
3596	c.157A>T		Pathogenic	not provided
3597	c.157A>G		Pathogenic	not provided
3598	c.157A>C		Benign	not provided
3599	c.156C>T		Benign	not provided
3600	c.156C>G		Pathogenic	not provided
3601	c.156C>A		Pathogenic	not provided
3602	c.155T>G		Benign	not provided
3603	c.155T>A		Benign	not provided
3604	c.154C>G		Benign	not provided
3605	c.153T>C		Benign	not provided
3606	c.153T>A		Benign	not provided
3607	c.152T>G		Benign	not provided
3608	c.152T>A		Benign	not provided
3609	c.151C>A		Benign	not provided
3610	c.151C>T		Benign	not provided
3611	c.151C>G		Benign	not provided
3612	c.150A>C		Benign	not provided
3613	c.149A>T		Pathogenic	not provided
3614	c.149A>G		Benign	not provided
3615	c.149A>C		Benign	not provided
3616	c.148A>T		Pathogenic	not provided
3617	c.148A>G		Benign	not provided

3618	c.148A>C	Benign	not provided
3619	c.147G>T	Pathogenic	not provided
3620	c.147G>C	Benign	not provided
3621	c.146T>C	Benign	not provided
3622	c.146T>A	Benign	not provided
3623	c.145C>T	Benign	not provided
3624	c.145C>G	Benign	not provided
3625	c.145C>A	Benign	not provided
3626	c.144G>T	Benign	not provided
3627	c.144G>C	Benign	not provided
3628	c.143T>G	Benign	not provided
3629	c.143T>C	Benign	not provided
3630	c.143T>A	Benign	not provided
3631	c.142A>T	Benign	not provided
3632	c.142A>C	Benign	not provided
3633	c.141C>G	Benign	not provided
3634	c.140G>C	Benign	not provided
3635	c.138T>G	Benign	not provided
3636	c.138T>C	Benign	not provided
3637	c.138T>A	Benign	not provided
3638	c.137T>G	Benign	not provided
3639	c.137T>C	Benign	not provided
3640	c.137T>A	Pathogenic	not provided
3641	c.136T>G	Benign	not provided
3642	c.136T>C	Benign	not provided
3643	c.136T>A	Benign	not provided
3644	c.135A>G	Benign	not provided
3645	c.135A>C	Benign	not provided
3646	c.135-2A>T	Pathogenic	not provided
3647	c.135-2A>C	Benign	not provided
3648	c.135-3T>G	Benign	not provided
3649	c.135-3T>A	Benign	not provided
3650	c.135-4A>T	Benign	not provided
3651	c.135-4A>C	Benign	not provided
3652	c.135-5T>G	Benign	not provided
3653	c.135-5T>A	Benign	not provided
3654	c.135-6T>C	Benign	not provided
3655	c.135-6T>A	Pathogenic	not provided
3656	c.135-7T>G	Pathogenic	not provided
3657	c.135-7T>A	Benign	not provided
3658	c.135-8A>T	Pathogenic	not provided
3659	c.135-8A>G	Benign	not provided
3660	c.135-8A>C	Benign	not provided
3661	c.135-9A>T	Pathogenic	not provided
3662	c.135-9A>G	Pathogenic	not provided
3663	c.135-9A>C	Benign	not provided
3664	c.135-10T>G	Benign	not provided
3665	c.135-10T>C	Benign	not provided
3666	c.135-10T>A	Benign	not provided
3667	c.135-11A>T	Benign	not provided
3668	c.135-11A>C	Benign	not provided
3669	c.135-12T>G	Benign	not provided
3670	c.135-12T>C	Benign	not provided
3671	c.135-12T>A	Benign	not provided

3672	c.134+23G>T	Benign	not provided
3673	c.134+23G>A	Benign	not provided
3674	c.134+22G>T	Benign	not provided
3675	c.134+22G>C	Benign	not provided
3676	c.134+22G>A	Pathogenic	not provided
3677	c.134+21T>G	Benign	not provided
3678	c.134+21T>C	Benign	not provided
3679	c.134+21T>A	Benign	not provided
3680	c.134+20G>T	Benign	not provided
3681	c.134+20G>C	Benign	not provided
3682	c.134+20G>A	Pathogenic	not provided
3683	c.134+19T>G	Benign	not provided
3684	c.134+19T>A	Benign	not provided
3685	c.134+18A>C	Benign	not provided
3686	c.134+17T>G	Benign	not provided
3687	c.134+17T>C	Benign	not provided
3688	c.134+17T>A	Benign	not provided
3689	c.134+16T>G	Benign	not provided
3690	c.134+16T>C	Benign	not provided
3691	c.134+16T>A	Benign	not provided
3692	c.134+15G>T	Benign	not provided
3693	c.134+15G>C	Benign	not provided
3694	c.134+14T>G	Benign	not provided
3695	c.134+14T>C	Benign	not provided
3696	c.134+14T>A	Benign	not provided
3697	c.134+13G>T	Benign	not provided
3698	c.134+13G>C	Benign	not provided
3699	c.134+12T>G	Benign	not provided
3700	c.134+12T>C	Benign	not provided
3701	c.134+12T>A	Benign	not provided
3702	c.134+11A>T	Benign	not provided
3703	c.134+11A>G	Benign	not provided
3704	c.134+11A>C	Benign	not provided
3705	c.134+10A>T	Pathogenic	not provided
3706	c.134+10A>G	Benign	not provided
3707	c.134+10A>C	Benign	not provided
3708	c.134+9G>T	Pathogenic	not provided
3709	c.134+8T>G	Benign	not provided
3710	c.134+8T>C	Benign	not provided
3711	c.134+8T>A	Benign	not provided
3712	c.134+7T>G	Benign	not provided
3713	c.134+7T>C	Benign	not provided
3714	c.134+7T>A	Benign	not provided
3715	c.134+6T>G	Benign	not provided
3716	c.134+6T>C	Benign	not provided
3717	c.134+6T>A	Benign	not provided
3718	c.134+4A>T	Pathogenic	not provided
3719	c.134+4A>G	Benign	not provided
3720	c.134+2T>A	Benign	not provided
3721	c.134A>T	Pathogenic	not provided
3722	c.134A>G	Benign	not provided
3723	c.133A>T	Pathogenic	not provided
3724	c.133A>G	Benign	not provided
3725	c.132C>A	Pathogenic	not provided

3726	c.131G>C		Benign	not provided
3727	c.130T>G	rs80357327	Benign	not provided
3728	c.130T>C	rs80357327	Benign	not provided
3729	c.129T>G		Benign	not provided
3730	c.129T>C		Benign	not provided
3731	c.129T>A		Benign	not provided
3732	c.128T>G		Benign	not provided
3733	c.128T>A		Benign	not provided
3734	c.127T>G		Benign	not provided
3735	c.127T>A		Benign	not provided
3736	c.126A>T		Benign	not provided
3737	c.126A>G		Pathogenic	not provided
3738	c.126A>C		Benign	not provided
3739	c.125T>G		Benign	not provided
3740	c.125T>C		Benign	not provided
3741	c.125T>A		Benign	not provided
3742	c.124A>T		Benign	not provided
3743	c.123C>G		Pathogenic	not provided
3744	c.123C>A		Benign	not provided
3745	c.122A>C		Benign	not provided
3746	c.121C>G		Benign	not provided
3747	c.120C>G		Benign	not provided
3748	c.120C>A		Benign	not provided
3749	c.119A>T		Benign	not provided
3750	c.119A>G		Benign	not provided
3751	c.119A>C		Benign	not provided
3752	c.118G>T		Pathogenic	not provided
3753	c.118G>C		Benign	not provided
3754	c.117T>C		Benign	not provided
3755	c.116G>C		Pathogenic	not provided
3756	c.114G>C		Benign	not provided
3757	c.113A>T		Pathogenic	not provided
3758	c.113A>G		Benign	not provided
3759	c.113A>C		Benign	not provided
3760	c.112A>T		Pathogenic	not provided
3761	c.112A>G		Benign	not provided
3762	c.112A>C		Benign	not provided
3763	c.111A>T		Pathogenic	not provided
3764	c.110C>T		Benign	not provided
3765	c.109A>T		Benign	not provided
3766	c.109A>G		Benign	not provided
3767	c.109A>C		Benign	not provided
3768	c.106T>G		Benign	not provided
3769	c.105C>T		Benign	not provided
3770	c.105C>G		Benign	not provided
3771	c.105C>A		Benign	not provided
3772	c.104T>G		Benign	not provided
3773	c.104T>C		Benign	not provided
3774	c.104T>A		Benign	not provided
3775	c.103G>C		Benign	not provided
3776	c.103G>A		Pathogenic	not provided
3777	c.102T>C		Benign	not provided
3778	c.102T>A		Benign	not provided
3779	c.101C>G		Benign	not provided

3780	c.101C>A	Benign	not provided
3781	c.100C>G	Benign	not provided
3782	c.100C>A	Benign	not provided
3783	c.99A>T	Benign	not provided
3784	c.99A>G	Benign	not provided
3785	c.99A>C	Benign	not provided
3786	c.98A>T	Pathogenic	not provided
3787	c.98A>G	Benign	not provided
3788	c.97G>A	Benign	not provided
3789	c.96G>T	Benign	not provided
3790	c.96G>A	Benign	not provided
3791	c.95A>T	Pathogenic	not provided
3792	c.95A>G	Benign	not provided
3793	c.95A>C	Benign	not provided
3794	c.94A>T	Pathogenic	not provided
3795	c.94A>G	Benign	not provided
3796	c.94A>C	Benign	not provided
3797	c.93C>T	Benign	not provided
3798	c.93C>A	Pathogenic	not provided
3799	c.92T>G	Benign	not provided
3800	c.92T>C	Benign	not provided
3801	c.92T>A	Benign	not provided
3802	c.91A>T	Benign	not provided
3803	c.91A>C	Benign	not provided
3804	c.90G>T	Pathogenic	not provided
3805	c.90G>C	Benign	not provided
3806	c.90G>A	Pathogenic	not provided
3807	c.89T>G	Benign	not provided
3808	c.89T>C	Benign	not provided
3809	c.88T>G	Benign	not provided
3810	c.88T>C	Benign	not provided
3811	c.88T>A	Benign	not provided
3812	c.87G>C	Benign	not provided
3813	c.87G>T	Benign	not provided
3814	c.86A>T	Pathogenic	not provided
3815	c.86A>C	Benign	not provided
3816	c.85G>C	Benign	not provided
3817	c.85G>A	Benign	not provided
3818	c.84G>T	Benign	not provided
3819	c.84G>C	Benign	not provided
3820	c.83T>G	Benign	not provided
3821	c.83T>A	Benign	not provided
3822	c.82C>A	Pathogenic	not provided
3823	c.81T>G	Benign	not provided
3824	c.81T>A	Benign	not provided
3825	c.81-1G>T	Benign	not provided
3826	c.81-2A>T	Pathogenic	not provided
3827	c.81-3T>G	Benign	not provided
3828	c.81-3T>C	Benign	not provided
3829	c.81-3T>A	Benign	not provided
3830	c.81-4C>G	Benign	not provided
3831	c.81-4C>A	Benign	not provided
3832	c.81-5G>T	Benign	not provided
3833	c.81-5G>C	Benign	not provided

3834	c.81-6T>G		Benign	not provided
3835	c.81-7C>T		Benign	not provided
3836	c.81-7C>G		Benign	not provided
3837	c.81-7C>A		Benign	not provided
3838	c.81-8C>T		Benign	not provided
3839	c.81-8C>G		Benign	not provided
3840	c.81-8C>A		Benign	not provided
3841	c.81-9C>T		Benign	not provided
3842	c.81-10A>T		Benign	not provided
3843	c.81-10A>G		Pathogenic	not provided
3844	c.81-10A>C		Benign	not provided
3845	c.81-11T>G		Benign	not provided
3846	c.81-11T>A		Benign	not provided
3847	c.81-12C>A		Benign	not provided
3848	c.81-15C>T		Benign	not provided
3849	c.81-15C>G		Benign	not provided
3850	c.81-16C>G		Benign	not provided
3851	c.81-17C>T		Benign	not provided
3852	c.81-18C>T		Benign	not provided
3853	c.81-19T>G		Benign	not provided
3854	c.81-19T>A		Benign	not provided
3855	c.81-20C>G		Benign	not provided
3856	c.81-20C>A		Benign	not provided
3857	c.81-21T>G		Benign	not provided
3858	c.81-21T>C		Benign	not provided
3859	c.81-21T>A		Benign	not provided
3860	c.81-22T>G		Benign	not provided
3861	c.81-22T>C		Benign	not provided
3862	c.81-22T>A		Benign	not provided
3863	c.81-23T>G		Benign	not provided
3864	c.81-23T>C		Benign	not provided
3865	c.81-23T>A		Benign	not provided
3866	c.80+4A>G		Benign	not provided
3867	c.80+4A>C		Benign	not provided
3868	c.80+3A>T		Benign	not provided
3869	c.80+3A>G		Pathogenic	not provided
3870	c.80+3A>C		Benign	not provided
3871	c.80+2T>C		Benign	not provided
3872	c.79T>G		Benign	not provided
3873	c.79T>C		Benign	not provided
3874	c.79T>A		Benign	not provided
3875	c.78C>G		Pathogenic	not provided
3876	c.78C>A		Benign	not provided
3877	c.77T>G		Benign	not provided
3878	c.76A>G		Benign	not provided
3879	c.76A>C		Benign	not provided
3880	c.74C>G		Benign	not provided
3881	c.73C>T		Benign	not provided
3882	c.73C>G		Benign	not provided
3883	c.73C>A	rs397509313	Benign	not provided
3884	c.72T>C		Benign	not provided
3885	c.72T>A		Benign	not provided
3886	c.71G>T		Benign	not provided
3887	c.71G>C		Benign	not provided

3888	c.69G>T	Benign	not provided
3889	c.68A>T	Pathogenic	not provided
3890	c.68A>G	Benign	not provided
3891	c.67G>T	Pathogenic	not provided
3892	c.67G>A	Pathogenic	not provided
3893	c.66A>T	Pathogenic	not provided
3894	c.65T>G	Benign	not provided
3895	c.64T>G	Benign	not provided
3896	c.64T>A	Benign	not provided
3897	c.63C>G	Benign	not provided
3898	c.63C>A	Benign	not provided
3899	c.62T>A	Benign	not provided
3900	c.62T>C	Benign	not provided
3901	c.61A>T	Benign	not provided
3902	c.61A>C	Benign	not provided
3903	c.60A>T	Pathogenic	not provided
3904	c.59A>T	Pathogenic	not provided
3905	c.59A>G	Benign	not provided
3906	c.59A>C	Benign	not provided
3907	c.58A>T	Pathogenic	not provided
3908	c.58A>G	Benign	not provided
3909	c.58A>C	Benign	not provided
3910	c.57G>T	Pathogenic	not provided
3911	c.57G>C	Benign	not provided
3912	c.57G>A	Benign	not provided
3913	c.56A>T	Pathogenic	not provided
3914	c.56A>C	Benign	not provided
3915	c.55C>G	Benign	not provided
3916	c.55C>A	Benign	not provided
3917	c.54G>T	Benign	not provided
3918	c.54G>C	Pathogenic	not provided
3919	c.52A>T	Benign	not provided
3920	c.52A>G	Pathogenic	not provided
3921	c.52A>C	Benign	not provided
3922	c.51T>C	Benign	not provided
3923	c.51T>A	Benign	not provided
3924	c.51T>G	Benign	not provided
3925	c.50C>T	Benign	not provided
3926	c.50C>G	Benign	not provided
3927	c.50C>A	Benign	not provided
3928	c.49G>C	Benign	not provided
3929	c.49G>A	Pathogenic	not provided
3930	c.48T>G	Benign	not provided
3931	c.47A>T	Benign	not provided
3932	c.47A>G	Benign	not provided
3933	c.47A>C	Benign	not provided
3934	c.46A>T	Benign	not provided
3935	c.46A>G	Pathogenic	not provided
3936	c.46A>C	Benign	not provided
3937	c.45T>G	Benign	not provided
3938	c.45T>C	Benign	not provided
3939	c.45T>A	Benign	not provided
3940	c.44T>G	Benign	not provided
3941	c.44T>A	Benign	not provided

3942	c.43A>T	Benign	not provided
3943	c.43A>G	Pathogenic	not provided
3944	c.42C>G	Benign	not provided
3945	c.41T>G	Benign	not provided
3946	c.41T>C	Benign	not provided
3947	c.41T>A	Benign	not provided
3948	c.40G>C	Benign	not provided
3949	c.40G>A	Pathogenic	not provided
3950	c.39T>G	Benign	not provided
3951	c.39T>C	Benign	not provided
3952	c.39T>A	Benign	not provided
3953	c.38A>T	Benign	not provided
3954	c.38A>G	Benign	not provided
3955	c.38A>C	Benign	not provided
3956	c.37A>T	Pathogenic	not provided
3957	c.37A>C	Benign	not provided
3958	c.36A>T	Pathogenic	not provided
3959	c.36A>C	Benign	not provided
3960	c.35A>T	Pathogenic	not provided
3961	c.35A>C	Benign	not provided
3962	c.34C>A	Benign	not provided
3963	c.33A>T	Benign	not provided
3964	c.33A>C	Benign	not provided
3965	c.32T>A	Benign	not provided
3966	c.31G>T	Benign	not provided
3967	c.31G>A	Benign	not provided
3968	c.30A>C	Benign	not provided
3969	c.30A>T	Pathogenic	not provided
3970	c.30A>G	Benign	not provided
3971	c.29A>T	Pathogenic	not provided
3972	c.29A>G	Benign	not provided
3973	c.29A>C	Benign	not provided
3974	c.28G>T	Pathogenic	not provided
3975	c.28G>C	Benign	not provided
3976	c.28G>A	Benign	not provided
3977	c.27A>T	Pathogenic	not provided
3978	c.27A>G	Benign	not provided
3979	c.27A>C	Benign	not provided
3980	c.26A>T	Pathogenic	not provided
3981	c.26A>G	Benign	not provided
3982	c.26A>C	Benign	not provided
3983	c.25G>T	Pathogenic	not provided
3984	c.25G>A	Pathogenic	not provided
3985	c.24T>G	Benign	not provided
3986	c.24T>C	Benign	not provided
3987	c.24T>A	Benign	not provided
3988	c.23T>G	Benign	not provided
3989	c.23T>C	Benign	not provided
3990	c.23T>A	Benign	not provided
3991	c.22G>T	Benign	not provided
3992	c.22G>C	Benign	not provided
3993	c.21C>G	Benign	not provided
3994	c.21C>A	Benign	not provided
3995	c.20G>C	Benign	not provided

3996	c.19C>G	Benign	not provided
3997	c.18T>G	Benign	not provided
3998	c.18T>C	Benign	not provided
3999	c.18T>A	Benign	not provided
4000	c.17T>G	Benign	not provided
4001	c.17T>C	Benign	not provided
4002	c.17T>A	Benign	not provided
4003	c.16C>G	Benign	not provided
4004	c.16C>A	Benign	not provided
4005	c.15T>G	Benign	not provided
4006	c.15T>A	Benign	not provided
4007	c.14C>G	Benign	not provided
4008	c.14C>A	Benign	not provided
4009	c.13G>T	Benign	not provided
4010	c.13G>A	Benign	not provided
4011	c.12T>G	Benign	not provided
4012	c.12T>C	Benign	not provided
4013	c.12T>A	Benign	not provided
4014	c.11C>G	Pathogenic	not provided
4015	c.11C>A	Benign	not provided
4016	c.10T>G	Benign	not provided
4017	c.10T>A	Benign	not provided
4018	c.9A>C	Benign	not provided
4019	c.8T>C	Benign	not provided
4020	c.8T>A	Benign	not provided
4021	c.7T>G	Benign	not provided
4022	c.7T>C	Benign	not provided
4023	c.7T>A	Benign	not provided
4024	c.6T>G	Benign	not provided
4025	c.6T>A	Benign	not provided
4026	c.5A>T	Benign	not provided
4027	c.5A>G	Benign	not provided
4028	c.5A>C	Benign	not provided
4029	c.4G>T	Pathogenic	not provided
4030	c.4G>C	Benign	not provided
4031	c.4G>A	Benign	not provided
4032	c.1A>T	Benign	not provided
4033	c.1A>C	Benign	not provided
4034	c.-1A>T	Pathogenic	not provided
4035	c.-2A>G	Benign	not provided
4036	c.-2A>C	Benign	not provided
4037	c.-3G>A	Benign	not provided
4038	c.-4A>T	Pathogenic	not provided
4039	c.-4A>G	Benign	not provided
4040	c.-4A>C	Benign	not provided
4041	c.-5A>T	Pathogenic	not provided
4042	c.-5A>G	Benign	not provided
4043	c.-5A>C	Benign	not provided
4044	c.-6A>T	Pathogenic	not provided
4045	c.-6A>G	Benign	not provided
4046	c.-6A>C	Benign	not provided
4047	c.-7G>T	Pathogenic	not provided
4048	c.-7G>C	Benign	not provided
4049	c.-7G>A	Benign	not provided

4050	c.-8A>T		Pathogenic	not provided
4051	c.-8A>G		Pathogenic	not provided
4052	c.-8A>C		Benign	not provided
4053	c.-9C>T		Benign	not provided
4054	c.-9C>A		Benign	not provided
4055	c.-10A>T		Benign	not provided
4056	c.-10A>G		Benign	not provided
4057	c.-11A>T		Pathogenic	not provided
4058	c.-11A>G		Benign	not provided
4059	c.-12G>T		Pathogenic	not provided
4060	c.-12G>C		Benign	not provided
4061	c.-12G>A		Benign	not provided
4062	c.-13G>T		Benign	not provided
4063	c.-13G>C		Benign	not provided
4064	c.-14T>A		Benign	not provided
4065	c.-14T>G		Benign	not provided
4066	c.-15T>G		Benign	not provided
4067	c.-15T>A		Benign	not provided
4068	c.-16A>T		Benign	not provided
4069	c.-16A>C		Benign	not provided
4070	c.-17C>G		Benign	not provided
4071	c.-17C>A		Benign	not provided
4072	c.-18T>G		Benign	not provided
4073	c.-18T>C		Benign	not provided
4074	c.-18T>A		Benign	not provided
4075	c.-19T>G		Benign	not provided
4076	c.-19T>C		Benign	not provided
4077	c.-19T>A		Benign	not provided
4078	c.-19-1G>T		Benign	not provided
4079	c.-19-1G>C		Benign	not provided
4080	c.-19-2A>T		Pathogenic	not provided
4081	c.-19-2A>C		Benign	not provided
4082	c.-19-3A>T		Pathogenic	not provided
4083	c.-19-3A>C		Benign	not provided
4084	c.*1363A>T	rs1332532415	Benign	Uncertain significance
4085	c.*1323A>G	rs189382442	Benign	Uncertain significance
4086	c.*1292T>C	rs182218567	Benign	Uncertain significance
4087	c.*1285C>A	rs757676381	Pathogenic	Uncertain significance
4088	c.*1271T>C	rs1555574034	Benign	Uncertain significance
4089	c.*800T>C	rs1555574136	Benign	Uncertain significance
4090	c.*743G>T	rs886052971	Benign	Uncertain significance
4091	c.*693C>T	rs540031582	Benign	Uncertain significance
4092	c.*485G>A	rs527725740	Pathogenic	Uncertain significance
4093	c.*465G>A	rs886052972	Benign	Uncertain significance
4094	c.*387T>G	rs886052973	Benign	Uncertain significance
4095	c.*291C>T	rs878854928	Benign	Uncertain significance
4096	c.*58C>T	rs137892861	Benign	Uncertain significance
4097	c.*42T>A	rs273902777	Benign	Uncertain significance
4098	c.*14C>G	rs1064796142	Benign	Uncertain significance
4099	c.*13A>G	rs762552027	Benign	Uncertain significance
4100	c.5588A>G	rs1567756242	Pathogenic	Uncertain significance
4101	c.5587T>G	rs763740623	Benign	Uncertain significance
4102	c.5585A>G	rs80357183	Benign	Uncertain significance
4103	c.5585A>T	rs80357183	Benign	Uncertain significance

4104	c.5580C>G		Benign	Uncertain significance
4105	c.5575C>T	rs1555574342	Benign	Uncertain significance
4106	c.5575C>A	rs1555574342	Pathogenic	Uncertain significance
4107	c.5574C>G	rs876659941	Pathogenic	Uncertain significance
4108	c.5573T>A		Benign	Uncertain significance
4109	c.5572A>T	rs765656957	Benign	Uncertain significance
4110	c.5571G>T	rs28897699	Pathogenic	Uncertain significance
4111	c.5571G>C	rs28897699	Benign	Uncertain significance
4112	c.5566C>A	rs80357274	Pathogenic	Uncertain significance
4113	c.5564T>C	rs900082291	Benign	Uncertain significance
4114	c.5561T>C	rs80356996	Benign	Uncertain significance
4115	c.5555C>T	rs730881502	Benign	Uncertain significance
4116	c.5554A>G	rs1555574370	Benign	Uncertain significance
4117	c.5553C>A	rs80357326	Benign	Uncertain significance
4118	c.5551G>A		Pathogenic	Uncertain significance
4119	c.5540G>T	rs1182000313	Benign	Uncertain significance
4120	c.5538G>C	rs80356849	Benign	Uncertain significance
4121	c.5536C>A		Pathogenic	Uncertain significance
4122	c.5534A>G	rs876660280	Pathogenic	Uncertain significance
4123	c.5531T>C	rs80357323	Benign	Uncertain significance
4124	c.5530C>A	rs1471435242	Benign	Uncertain significance
4125	c.5528C>T	rs730881447	Benign	Uncertain significance
4126	c.5528C>A	rs730881447	Benign	Uncertain significance
4127	c.5525T>A	rs1555574400	Benign	Uncertain significance
4128	c.5522G>C	rs80357368	Benign	Uncertain significance
4129	c.5522G>A	rs80357368	Pathogenic	Uncertain significance
4130	c.5521A>G		Pathogenic	Uncertain significance
4131	c.5519A>C		Benign	Uncertain significance
4132	c.5518G>T		Pathogenic	Uncertain significance
4133	c.5518G>A	rs1567756588	Pathogenic	Uncertain significance
4134	c.5512G>T	rs730881501	Benign	Uncertain significance
4135	c.5512G>A	rs730881501	Pathogenic	Uncertain significance
4136	c.5511G>C	rs80356914	Pathogenic	Uncertain significance
4137	c.5506G>A	rs80356942	Pathogenic	Uncertain significance
4138	c.5504G>C	rs273902776	Benign	Uncertain significance
4139	c.5504G>A	rs273902776	Benign	Uncertain significance
4140	c.5501C>A		Pathogenic	Uncertain significance
4141	c.5501C>T	rs730881500	Benign	Uncertain significance
4142	c.5498T>C	rs587782340	Benign	Uncertain significance
4143	c.5492C>G	rs587782778	Benign	Uncertain significance
4144	c.5488G>A	rs80357393	Benign	Uncertain significance
4145	c.5485G>A	rs869320789	Pathogenic	Uncertain significance
4146	c.5482T>G	rs1060502342	Benign	Uncertain significance
4147	c.5481G>A	rs587782432	Pathogenic	Uncertain significance
4148	c.5479A>G		Benign	Uncertain significance
4149	c.5478G>T	rs80357332	Pathogenic	Uncertain significance
4150	c.5477A>T	rs730881499	Pathogenic	Uncertain significance
4151	c.5476C>A	rs587782887	Benign	Uncertain significance
4152	c.5473G>A	rs398122700	Pathogenic	Uncertain significance
4153	c.5470A>G	rs587782026	Benign	Uncertain significance
4154	c.5468-4C>A		Benign	Uncertain significance
4155	c.5458G>A	rs398122698	Pathogenic	Uncertain significance
4156	c.5447C>G		Benign	Uncertain significance
4157	c.5447C>A	rs1567757864	Pathogenic	Uncertain significance

4158	c.5444G>C	rs80356962	Benign	Uncertain significance
4159	c.5441C>A		Benign	Uncertain significance
4160	c.5438A>T	rs1555574715	Benign	Uncertain significance
4161	c.5437G>T	rs1303996018	Pathogenic	Uncertain significance
4162	c.5433G>C	rs4438367	Benign	Uncertain significance
4163	c.5429T>C	rs80357451	Benign	Uncertain significance
4164	c.5429T>G	rs80357451	Benign	Uncertain significance
4165	c.5426T>C	rs80357216	Benign	Uncertain significance
4166	c.5425G>A	rs28897698	Pathogenic	Uncertain significance
4167	c.5423T>G	rs80357358	Benign	Uncertain significance
4168	c.5423T>A	rs80357358	Benign	Uncertain significance
4169	c.5423T>C	rs80357358	Benign	Uncertain significance
4170	c.5422G>T	rs1555574756	Benign	Uncertain significance
4171	c.5419A>T		Benign	Uncertain significance
4172	c.5416C>T		Benign	Uncertain significance
4173	c.5413C>T	rs587782873	Benign	Uncertain significance
4174	c.5408G>T	rs80357149	Benign	Uncertain significance
4175	c.5407G>T		Benign	Uncertain significance
4176	c.5407G>A	rs876659510	Pathogenic	Uncertain significance
4177	c.5404A>C	rs1555575080	Benign	Uncertain significance
4178	c.5401G>A	rs587776492	Pathogenic	Uncertain significance
4179	c.5399T>G		Benign	Uncertain significance
4180	c.5396C>T	rs786201945	Benign	Uncertain significance
4181	c.5387C>T	rs80357055	Benign	Uncertain significance
4182	c.5383C>A	rs878854958	Benign	Uncertain significance
4183	c.5383C>T	rs878854958	Benign	Uncertain significance
4184	c.5378A>C	rs730881446	Benign	Uncertain significance
4185	c.5374G>A	rs1555575131	Pathogenic	Uncertain significance
4186	c.5374G>C	rs1555575131	Benign	Uncertain significance
4187	c.5372T>C	rs864622244	Benign	Uncertain significance
4188	c.5371G>A	rs145758886	Pathogenic	Uncertain significance
4189	c.5371G>T	rs145758886	Benign	Uncertain significance
4190	c.5368T>C	rs1567758993	Benign	Uncertain significance
4191	c.5365G>C	rs80357078	Benign	Uncertain significance
4192	c.5363G>A	rs80357069	Pathogenic	Uncertain significance
4193	c.5360G>A		Pathogenic	Uncertain significance
4194	c.5354A>C	rs876660057	Benign	Uncertain significance
4195	c.5350G>A	rs1060502328	Pathogenic	Uncertain significance
4196	c.5349G>A	rs587782019	Pathogenic	Uncertain significance
4197	c.5347A>G	rs80357012	Benign	Uncertain significance
4198	c.5343A>C	rs1555575182	Benign	Uncertain significance
4199	c.5341G>A		Pathogenic	Uncertain significance
4200	c.5339T>A	rs80357474	Benign	Uncertain significance
4201	c.5339T>G	rs80357474	Benign	Uncertain significance
4202	c.5335C>G		Pathogenic	Uncertain significance
4203	c.5332+78C>A	rs273901763	Benign	Uncertain significance
4204	c.5332G>C	rs80357112	Benign	Uncertain significance
4205	c.5330C>T	rs398122696	Benign	Uncertain significance
4206	c.5329A>T	rs879255296	Benign	Uncertain significance
4207	c.5327C>T	rs398122695	Benign	Uncertain significance
4208	c.5326C>T	rs1800757	Benign	Uncertain significance
4209	c.5325G>A	rs1135401885	Pathogenic	Uncertain significance
4210	c.5324T>C		Benign	Uncertain significance
4211	c.5321A>G	rs587781770	Benign	Uncertain significance

4212	c.5320A>C		Benign	Uncertain significance
4213	c.5318C>T	rs80357428	Benign	Uncertain significance
4214	c.5315T>G	rs1555575700	Benign	Uncertain significance
4215	c.5312C>T	rs80357025	Benign	Uncertain significance
4216	c.5312C>G	rs80357025	Benign	Uncertain significance
4217	c.5311C>T		Benign	Uncertain significance
4218	c.5309G>C	rs863224765	Pathogenic	Uncertain significance
4219	c.5303G>A	rs730881497	Pathogenic	Uncertain significance
4220	c.5302T>G	rs431825416	Benign	Uncertain significance
4221	c.5300G>C		Benign	Uncertain significance
4222	c.5299T>C	rs1567760645	Benign	Uncertain significance
4223	c.5296A>G	rs886039314	Benign	Uncertain significance
4224	c.5288G>T	rs80357007	Benign	Uncertain significance
4225	c.5287G>C	rs876660907	Pathogenic	Uncertain significance
4226	c.5285G>C		Benign	Uncertain significance
4227	c.5285G>T	rs398122694	Benign	Uncertain significance
4228	c.5282T>G	rs80356905	Benign	Uncertain significance
4229	c.5281T>C	rs1567760717	Benign	Uncertain significance
4230	c.5277+77C>A	rs273901758	Benign	Uncertain significance
4231	c.5277+7C>T		Benign	Uncertain significance
4232	c.5276A>G	rs431825415	Benign	Uncertain significance
4233	c.5272A>G		Pathogenic	Uncertain significance
4234	c.5271C>A		Benign	Uncertain significance
4235	c.5269G>A	rs863224764	Pathogenic	Uncertain significance
4236	c.5269G>T	rs863224764	Pathogenic	Uncertain significance
4237	c.5269G>C	rs863224764	Benign	Uncertain significance
4238	c.5267A>G	rs1567764257	Pathogenic	Uncertain significance
4239	c.5265C>T	rs1555576855	Benign	Uncertain significance
4240	c.5264C>T	rs1555576858	Benign	Uncertain significance
4241	c.5260G>C	rs80357432	Benign	Uncertain significance
4242	c.5259A>T	rs771577266	Pathogenic	Uncertain significance
4243	c.5255C>G	rs80357028	Benign	Uncertain significance
4244	c.5255C>T	rs80357028	Pathogenic	Uncertain significance
4245	c.5254G>A	rs80357074	Pathogenic	Uncertain significance
4246	c.5252G>T	rs80357442	Benign	Uncertain significance
4247	c.5251C>G	rs80357123	Benign	Uncertain significance
4248	c.5246C>T	rs80357462	Benign	Uncertain significance
4249	c.5245C>A	rs397509244	Benign	Uncertain significance
4250	c.5243G>T	rs397509243	Pathogenic	Uncertain significance
4251	c.5238C>G	rs786202389	Benign	Uncertain significance
4252	c.5237A>C	rs876659991	Benign	Uncertain significance
4253	c.5236C>G	rs80357146	Benign	Uncertain significance
4254	c.5236C>T	rs80357146	Benign	Uncertain significance
4255	c.5236C>A	rs80357146	Benign	Uncertain significance
4256	c.5234A>G	rs1567764456	Benign	Uncertain significance
4257	c.5231G>A	rs1567764460	Pathogenic	Uncertain significance
4258	c.5228G>C	rs1346819781	Benign	Uncertain significance
4259	c.5225A>G	rs864622104	Benign	Uncertain significance
4260	c.5224A>G		Pathogenic	Uncertain significance
4261	c.5222T>G	rs80357023	Benign	Uncertain significance
4262	c.5221G>A	rs876659122	Pathogenic	Uncertain significance
4263	c.5219T>C	rs1555576951	Benign	Uncertain significance
4264	c.5217T>G	rs80357340	Benign	Uncertain significance
4265	c.5215G>C	rs80357283	Benign	Uncertain significance

4266	c.5215G>A	rs80357283	Pathogenic	Uncertain significance
4267	c.5215G>T	rs80357283	Pathogenic	Uncertain significance
4268	c.5205A>T	rs431825412	Pathogenic	Uncertain significance
4269	c.5202T>G	rs869320780	Benign	Uncertain significance
4270	c.5201T>G		Benign	Uncertain significance
4271	c.5200T>A	rs80356957	Pathogenic	Uncertain significance
4272	c.5200T>C	rs80356957	Benign	Uncertain significance
4273	c.5198A>T	rs80357270	Benign	Uncertain significance
4274	c.5197G>C	rs398122693	Benign	Uncertain significance
4275	c.5197G>A	rs398122693	Pathogenic	Uncertain significance
4276	c.5196T>A		Benign	Uncertain significance
4277	c.5195A>G		Benign	Uncertain significance
4278	c.5193+13T>A	rs273901750	Benign	Uncertain significance
4279	c.5191G>A	rs397507244	Pathogenic	Uncertain significance
4280	c.5189A>T	rs80357171	Benign	Uncertain significance
4281	c.5186T>C		Benign	Uncertain significance
4282	c.5186T>A	rs730881496	Benign	Uncertain significance
4283	c.5179A>C	rs80357347	Benign	Uncertain significance
4284	c.5177G>C	rs786203547	Benign	Uncertain significance
4285	c.5177G>T	rs786203547	Pathogenic	Uncertain significance
4286	c.5175A>C	rs191373374	Benign	Uncertain significance
4287	c.5172A>C	rs879254150	Benign	Uncertain significance
4288	c.5168T>C	rs1064793533	Benign	Uncertain significance
4289	c.5168T>A	rs1064793533	Benign	Uncertain significance
4290	c.5167A>G	rs1426821558	Pathogenic	Uncertain significance
4291	c.5165C>A	rs80357104	Pathogenic	Uncertain significance
4292	c.5161C>G	rs878854957	Benign	Uncertain significance
4293	c.5156T>C		Benign	Uncertain significance
4294	c.5156T>G	rs1247437511	Benign	Uncertain significance
4295	c.5155G>A		Pathogenic	Uncertain significance
4296	c.5155G>T	rs749465132	Benign	Uncertain significance
4297	c.5153G>T		Benign	Uncertain significance
4298	c.5153-42T>A	rs273901747	Benign	Uncertain significance
4299	c.5153-66T>A	rs273901748	Benign	Uncertain significance
4300	c.5152T>G	rs1567769155	Benign	Uncertain significance
4301	c.5147A>C	rs587782456	Benign	Uncertain significance
4302	c.5147A>G	rs587782456	Pathogenic	Uncertain significance
4303	c.5146T>C		Benign	Uncertain significance
4304	c.5146T>A		Benign	Uncertain significance
4305	c.5145C>A	rs80357094	Pathogenic	Uncertain significance
4306	c.5144G>T	rs45444999	Benign	Uncertain significance
4307	c.5141T>C	rs80357243	Benign	Uncertain significance
4308	c.5140G>T	rs1567769244	Benign	Uncertain significance
4309	c.5137G>A	rs1064793309	Pathogenic	Uncertain significance
4310	c.5133A>G		Benign	Uncertain significance
4311	c.5131A>C	rs886040272	Benign	Uncertain significance
4312	c.5129G>A	rs398122691	Benign	Uncertain significance
4313	c.5128G>A		Pathogenic	Uncertain significance
4314	c.5126G>A	rs786204269	Pathogenic	Uncertain significance
4315	c.5125G>A	rs886038197	Benign	Uncertain significance
4316	c.5123C>T	rs28897696	Benign	Uncertain significance
4317	c.5122G>A	rs397507243	Pathogenic	Uncertain significance
4318	c.5120T>C	rs1064796143	Benign	Uncertain significance
4319	c.5120T>G	rs1064796143	Benign	Uncertain significance

4320	c.5119A>C		Benign	Uncertain significance
4321	c.5116G>C	rs886040864	Benign	Uncertain significance
4322	c.5114T>G	rs397507242	Benign	Uncertain significance
4323	c.5113C>G	rs80356858	Benign	Uncertain significance
4324	c.5111T>A	rs1555578598	Pathogenic	Uncertain significance
4325	c.5110T>C	rs1555578599	Benign	Uncertain significance
4326	c.5107T>G	rs863224763	Benign	Uncertain significance
4327	c.5107T>C	rs863224763	Benign	Uncertain significance
4328	c.5102T>C		Benign	Uncertain significance
4329	c.5101C>A	rs910555398	Benign	Uncertain significance
4330	c.5098A>C	rs397509227	Benign	Uncertain significance
4331	c.5096G>T	rs41293459	Benign	Uncertain significance
4332	c.5096G>C	rs41293459	Benign	Uncertain significance
4333	c.5091T>G		Benign	Uncertain significance
4334	c.5089T>A	rs80356993	Benign	Uncertain significance
4335	c.5087T>C	rs397509226	Benign	Uncertain significance
4336	c.5086G>A	rs80357125	Pathogenic	Uncertain significance
4337	c.5086G>T	rs80357125	Benign	Uncertain significance
4338	c.5086G>C	rs80357125	Benign	Uncertain significance
4339	c.5085T>A	rs80357387	Pathogenic	Uncertain significance
4340	c.5081A>G	rs1567769562	Pathogenic	Uncertain significance
4341	c.5080G>A	rs80356896	Pathogenic	Uncertain significance
4342	c.5078C>T	rs1567769586	Benign	Uncertain significance
4343	c.5075A>C	rs397509222	Benign	Uncertain significance
4344	c.5075A>T	rs397509222	Benign	Uncertain significance
4345	c.5075-4G>T		Benign	Uncertain significance
4346	c.5074+7C>T		Benign	Uncertain significance
4347	c.5074+4T>A		Benign	Uncertain significance
4348	c.5073A>C	rs80356853	Benign	Uncertain significance
4349	c.5073A>G	rs80356853	Pathogenic	Uncertain significance
4350	c.5071A>G	rs397509219	Benign	Uncertain significance
4351	c.5067G>A		Pathogenic	Uncertain significance
4352	c.5066T>C	rs80357061	Benign	Uncertain significance
4353	c.5065A>C	rs1555579619	Benign	Uncertain significance
4354	c.5065A>G	rs1555579619	Pathogenic	Uncertain significance
4355	c.5064T>C	rs1567771967	Benign	Uncertain significance
4356	c.5063T>C		Benign	Uncertain significance
4357	c.5060T>G	rs1555579627	Benign	Uncertain significance
4358	c.5057A>C	rs730882166	Benign	Uncertain significance
4359	c.5056C>T	rs1555579648	Benign	Uncertain significance
4360	c.5050A>T		Pathogenic	Uncertain significance
4361	c.5050A>G	rs879255491	Benign	Uncertain significance
4362	c.5050A>C	rs879255491	Benign	Uncertain significance
4363	c.5048A>G		Pathogenic	Uncertain significance
4364	c.5045A>T	rs80357265	Pathogenic	Uncertain significance
4365	c.5044G>C		Benign	Uncertain significance
4366	c.5042C>T	rs766784305	Benign	Uncertain significance
4367	c.5041A>C	rs876659314	Benign	Uncertain significance
4368	c.5036T>C	rs760038328	Benign	Uncertain significance
4369	c.5035C>G		Benign	Uncertain significance
4370	c.5030C>T	rs876660263	Benign	Uncertain significance
4371	c.5028A>T	rs1064793596	Benign	Uncertain significance
4372	c.5023A>T		Benign	Uncertain significance
4373	c.5018A>G	rs1064793913	Benign	Uncertain significance

4374	c.5015A>T	rs1567772328	Benign	Uncertain significance
4375	c.5006C>T	rs1057518640	Benign	Uncertain significance
4376	c.5003T>G	rs1567772408	Benign	Uncertain significance
4377	c.5003T>C	rs1567772408	Benign	Uncertain significance
4378	c.5002T>A	rs587781472	Benign	Uncertain significance
4379	c.5000A>G	rs1057519495	Benign	Uncertain significance
4380	c.4999A>G	rs80357204	Benign	Uncertain significance
4381	c.4997A>G	rs397509216	Pathogenic	Uncertain significance
4382	c.4994T>G		Benign	Uncertain significance
4383	c.4993G>T		Benign	Uncertain significance
4384	c.4993G>C	rs80357169	Benign	Uncertain significance
4385	c.4988T>A	rs80357205	Benign	Uncertain significance
4386	c.4987A>T	rs80357117	Benign	Uncertain significance
4387	c.4981G>A	rs80357401	Pathogenic	Uncertain significance
4388	c.4973C>A	rs1015073230	Benign	Uncertain significance
4389	c.4967G>A	rs80357414	Benign	Uncertain significance
4390	c.4957G>A	rs80357261	Benign	Uncertain significance
4391	c.4955T>A	rs80356968	Benign	Uncertain significance
4392	c.4954A>G	rs1348949389	Benign	Uncertain significance
4393	c.4952C>T	rs80356938	Benign	Uncertain significance
4394	c.4951T>C	rs879254042	Benign	Uncertain significance
4395	c.4950G>A		Pathogenic	Uncertain significance
4396	c.4949T>C	rs778487856	Benign	Uncertain significance
4397	c.4946G>C	rs876660509	Benign	Uncertain significance
4398	c.4942A>G		Benign	Uncertain significance
4399	c.4934G>T	rs70953661	Benign	Uncertain significance
4400	c.4933A>G	rs80356926	Benign	Uncertain significance
4401	c.4931A>G	rs80357016	Benign	Uncertain significance
4402	c.4922C>T		Benign	Uncertain significance
4403	c.4921G>A	rs1800726	Pathogenic	Uncertain significance
4404	c.4918A>G	rs1555580683	Benign	Uncertain significance
4405	c.4916T>C	rs1567774676	Benign	Uncertain significance
4406	c.4910C>A	rs80357048	Pathogenic	Uncertain significance
4407	c.4909C>T	rs876659989	Benign	Uncertain significance
4408	c.4903G>C	rs200432771	Benign	Uncertain significance
4409	c.4903G>A	rs200432771	Pathogenic	Uncertain significance
4410	c.4900A>G		Pathogenic	Uncertain significance
4411	c.4899C>A	rs1567774741	Benign	Uncertain significance
4412	c.4897A>C	rs1555580711	Benign	Uncertain significance
4413	c.4895T>G	rs1397965282	Benign	Uncertain significance
4414	c.4894G>C	rs770193975	Pathogenic	Uncertain significance
4415	c.4894G>A	rs770193975	Pathogenic	Uncertain significance
4416	c.4894G>T	rs770193975	Benign	Uncertain significance
4417	c.4893T>A	rs80356850	Benign	Uncertain significance
4418	c.4892G>A	rs273901742	Benign	Uncertain significance
4419	c.4891A>T	rs786202734	Pathogenic	Uncertain significance
4420	c.4884G>T	rs80357158	Benign	Uncertain significance
4421	c.4879G>T		Benign	Uncertain significance
4422	c.4879G>A	rs774505084	Pathogenic	Uncertain significance
4423	c.4874A>G	rs730881494	Pathogenic	Uncertain significance
4424	c.4873T>A		Pathogenic	Uncertain significance
4425	c.4871G>A	rs1555580767	Pathogenic	Uncertain significance
4426	c.4865C>G	rs786202573	Benign	Uncertain significance
4427	c.4864A>G	rs786202026	Pathogenic	Uncertain significance

4428	c.4862A>C	rs1555580777	Benign	Uncertain significance
4429	c.4856C>T	rs876659163	Benign	Uncertain significance
4430	c.4853A>G	rs1277159752	Pathogenic	Uncertain significance
4431	c.4852C>T	rs755920262	Benign	Uncertain significance
4432	c.4852C>A	rs755920262	Benign	Uncertain significance
4433	c.4844C>T	rs1555580805	Benign	Uncertain significance
4434	c.4843G>C		Benign	Uncertain significance
4435	c.4843G>A	rs80356987	Pathogenic	Uncertain significance
4436	c.4841C>T	rs766305255	Benign	Uncertain significance
4437	c.4838G>A	rs1555580821	Benign	Uncertain significance
4438	c.4836G>C	rs747688901	Benign	Uncertain significance
4439	c.4826A>G	rs483353096	Benign	Uncertain significance
4440	c.4823C>T	rs80357072	Pathogenic	Uncertain significance
4441	c.4822G>A	rs1567775064	Pathogenic	Uncertain significance
4442	c.4814T>C		Benign	Uncertain significance
4443	c.4813T>A	rs80356833	Benign	Uncertain significance
4444	c.4813T>G	rs80356833	Benign	Uncertain significance
4445	c.4811A>G	rs80357439	Benign	Uncertain significance
4446	c.4810C>G		Benign	Uncertain significance
4447	c.4808C>T	rs1064794054	Benign	Uncertain significance
4448	c.4804G>T		Benign	Uncertain significance
4449	c.4801A>C	rs80357303	Benign	Uncertain significance
4450	c.4796C>T		Benign	Uncertain significance
4451	c.4795G>A		Pathogenic	Uncertain significance
4452	c.4790C>A	rs587781623	Benign	Uncertain significance
4453	c.4789A>G	rs80357187	Benign	Uncertain significance
4454	c.4787C>T	rs80357429	Benign	Uncertain significance
4455	c.4786T>G		Benign	Uncertain significance
4456	c.4783T>A		Benign	Uncertain significance
4457	c.4783T>C	rs1325644035	Benign	Uncertain significance
4458	c.4781C>A	rs1301724072	Benign	Uncertain significance
4459	c.4780C>T	rs587782012	Benign	Uncertain significance
4460	c.4780C>G	rs587782012	Benign	Uncertain significance
4461	c.4777A>G	rs397509197	Benign	Uncertain significance
4462	c.4774A>C	rs1567775261	Benign	Uncertain significance
4463	c.4772G>A	rs1555580956	Benign	Uncertain significance
4464	c.4771G>A	rs587782825	Pathogenic	Uncertain significance
4465	c.4769T>C	rs773524529	Benign	Uncertain significance
4466	c.4765C>G	rs80357002	Benign	Uncertain significance
4467	c.4751C>T	rs1060502323	Benign	Uncertain significance
4468	c.4748G>A	rs752624544	Pathogenic	Uncertain significance
4469	c.4739C>A	rs80357411	Pathogenic	Uncertain significance
4470	c.4739C>T	rs80357411	Benign	Uncertain significance
4471	c.4733A>G	rs80356930	Benign	Uncertain significance
4472	c.4730C>T	rs273901741	Benign	Uncertain significance
4473	c.4730C>A	rs273901741	Pathogenic	Uncertain significance
4474	c.4727A>G	rs876659007	Benign	Uncertain significance
4475	c.4726G>A	rs1060502355	Pathogenic	Uncertain significance
4476	c.4724C>A	rs80357052	Benign	Uncertain significance
4477	c.4717G>C	rs1188628175	Benign	Uncertain significance
4478	c.4713C>G	rs768945711	Benign	Uncertain significance
4479	c.4712T>C	rs273901740	Benign	Uncertain significance
4480	c.4704C>T	rs886037793	Benign	Uncertain significance
4481	c.4702A>G	rs80357119	Benign	Uncertain significance

4482	c.4700G>T	rs1555581074	Pathogenic	Uncertain significance
4483	c.4699G>A	rs568753972	Pathogenic	Uncertain significance
4484	c.4698T>G	rs1246456207	Benign	Uncertain significance
4485	c.4697C>G	rs1060502325	Pathogenic	Uncertain significance
4486	c.4697C>A	rs1060502325	Pathogenic	Uncertain significance
4487	c.4696T>G	rs730881493	Benign	Uncertain significance
4488	c.4693G>A	rs863224762	Pathogenic	Uncertain significance
4489	c.4685C>T	rs80357096	Benign	Uncertain significance
4490	c.4682C>A	rs56158747	Benign	Uncertain significance
4491	c.4679G>T	rs564757581	Pathogenic	Uncertain significance
4492	c.4677G>C	rs587781876	Benign	Uncertain significance
4493	c.4675+3A>C		Benign	Uncertain significance
4494	c.4674A>G	rs996042036	Pathogenic	Uncertain significance
4495	c.4673T>G		Benign	Uncertain significance
4496	c.4672C>G	rs1555581794	Benign	Uncertain significance
4497	c.4671T>G		Benign	Uncertain significance
4498	c.4670A>G	rs869320779	Benign	Uncertain significance
4499	c.4669G>A	rs80356906	Benign	Uncertain significance
4500	c.4669G>T	rs80356906	Pathogenic	Uncertain significance
4501	c.4669G>C	rs80356906	Benign	Uncertain significance
4502	c.4667A>G	rs1555581811	Benign	Uncertain significance
4503	c.4664G>A	rs786202165	Benign	Uncertain significance
4504	c.4649C>T	rs80357076	Benign	Uncertain significance
4505	c.4647A>C	rs1371814796	Benign	Uncertain significance
4506	c.4646A>T	rs876659001	Pathogenic	Uncertain significance
4507	c.4641G>C		Benign	Uncertain significance
4508	c.4641G>T	rs864622265	Pathogenic	Uncertain significance
4509	c.4639T>G	rs730881492	Pathogenic	Uncertain significance
4510	c.4639T>A	rs730881492	Pathogenic	Uncertain significance
4511	c.4636G>C	rs28897691	Benign	Uncertain significance
4512	c.4634A>G		Benign	Uncertain significance
4513	c.4631C>T	rs80356917	Benign	Uncertain significance
4514	c.4618G>C		Benign	Uncertain significance
4515	c.4616T>C	rs377629427	Benign	Uncertain significance
4516	c.4606G>C	rs876660460	Benign	Uncertain significance
4517	c.4603G>A	rs80357366	Pathogenic	Uncertain significance
4518	c.4597G>A	rs899108857	Pathogenic	Uncertain significance
4519	c.4597G>T	rs899108857	Pathogenic	Uncertain significance
4520	c.4594G>A	rs786201658	Pathogenic	Uncertain significance
4521	c.4591G>T		Pathogenic	Uncertain significance
4522	c.4591G>A	rs1555581897	Pathogenic	Uncertain significance
4523	c.4589A>C	rs398122689	Benign	Uncertain significance
4524	c.4588A>C		Benign	Uncertain significance
4525	c.4583T>C	rs1555581911	Benign	Uncertain significance
4526	c.4580A>G		Pathogenic	Uncertain significance
4527	c.4579G>A	rs80357237	Pathogenic	Uncertain significance
4528	c.4576G>A	rs878853294	Pathogenic	Uncertain significance
4529	c.4574A>G	rs786203386	Benign	Uncertain significance
4530	c.4571C>G	rs1555581944	Benign	Uncertain significance
4531	c.4568C>T	rs1555581955	Benign	Uncertain significance
4532	c.4567C>G		Pathogenic	Uncertain significance
4533	c.4564T>C	rs1567778091	Benign	Uncertain significance
4534	c.4559G>C	rs1555581971	Benign	Uncertain significance
4535	c.4555A>G	rs1567778111	Benign	Uncertain significance

4536	c.4552C>A	rs80356881	Pathogenic	Uncertain significance
4537	c.4552C>G	rs80356881	Pathogenic	Uncertain significance
4538	c.4550T>C	rs1555581980	Benign	Uncertain significance
4539	c.4549C>T		Benign	Uncertain significance
4540	c.4549C>G	rs137894496	Benign	Uncertain significance
4541	c.4541C>T	rs863224761	Benign	Uncertain significance
4542	c.4534A>G		Pathogenic	Uncertain significance
4543	c.4534A>T	rs80357137	Pathogenic	Uncertain significance
4544	c.4533C>A	rs1567778195	Benign	Uncertain significance
4545	c.4532A>C	rs1555582004	Benign	Uncertain significance
4546	c.4530G>A	rs1555582010	Pathogenic	Uncertain significance
4547	c.4529T>C	rs1064794378	Benign	Uncertain significance
4548	c.4526A>G	rs1555582023	Pathogenic	Uncertain significance
4549	c.4519A>G	rs1567778299	Pathogenic	Uncertain significance
4550	c.4514A>G		Benign	Uncertain significance
4551	c.4514A>T	rs1567778319	Benign	Uncertain significance
4552	c.4513G>C	rs1555582052	Benign	Uncertain significance
4553	c.4510T>G	rs1567778337	Benign	Uncertain significance
4554	c.4505C>T		Benign	Uncertain significance
4555	c.4504C>T	rs80357383	Benign	Uncertain significance
4556	c.4493C>G	rs1555582079	Benign	Uncertain significance
4557	c.4492C>T		Benign	Uncertain significance
4558	c.4485G>C	rs1064793833	Benign	Uncertain significance
4559	c.4484+3A>C		Benign	Uncertain significance
4560	c.4481A>G	rs758779691	Benign	Uncertain significance
4561	c.4480G>A	rs80357148	Pathogenic	Uncertain significance
4562	c.4477G>A	rs949577051	Benign	Uncertain significance
4563	c.4475G>A		Pathogenic	Uncertain significance
4564	c.4474G>A		Pathogenic	Uncertain significance
4565	c.4471C>T		Benign	Uncertain significance
4566	c.4471C>A	rs111034213	Benign	Uncertain significance
4567	c.4470A>T	rs1555582549	Benign	Uncertain significance
4568	c.4466A>G	rs587781880	Benign	Uncertain significance
4569	c.4463A>C	rs1567779520	Benign	Uncertain significance
4570	c.4460A>G	rs80357126	Benign	Uncertain significance
4571	c.4458T>G	rs879255285	Benign	Uncertain significance
4572	c.4456A>T	rs397507232	Pathogenic	Uncertain significance
4573	c.4454C>G		Benign	Uncertain significance
4574	c.4454C>T	rs80356870	Benign	Uncertain significance
4575	c.4450T>G	rs80357404	Benign	Uncertain significance
4576	c.4450T>A	rs80357404	Benign	Uncertain significance
4577	c.4449T>A	rs1555582581	Benign	Uncertain significance
4578	c.4447A>C	rs1555582583	Benign	Uncertain significance
4579	c.4446T>G	rs1555582586	Benign	Uncertain significance
4580	c.4445A>G		Benign	Uncertain significance
4581	c.4445A>T	rs757726297	Benign	Uncertain significance
4582	c.4441G>A	rs1135401828	Pathogenic	Uncertain significance
4583	c.4435G>C	rs786203524	Benign	Uncertain significance
4584	c.4434G>C	rs1555582594	Benign	Uncertain significance
4585	c.4430T>C	rs876660550	Benign	Uncertain significance
4586	c.4429T>G		Benign	Uncertain significance
4587	c.4427A>C	rs750437234	Benign	Uncertain significance
4588	c.4423G>T	rs876660940	Pathogenic	Uncertain significance
4589	c.4420G>A	rs1555582616	Pathogenic	Uncertain significance

4590	c.4417T>C	rs398122686	Benign	Uncertain significance
4591	c.4414C>T	rs200582930	Benign	Uncertain significance
4592	c.4412G>A	rs587782708	Benign	Uncertain significance
4593	c.4412G>T	rs587782708	Benign	Uncertain significance
4594	c.4412G>C	rs587782708	Benign	Uncertain significance
4595	c.4411G>A	rs1567779701	Benign	Uncertain significance
4596	c.4405C>A		Benign	Uncertain significance
4597	c.4405C>T	rs80356960	Benign	Uncertain significance
4598	c.4402A>G	rs80357022	Benign	Uncertain significance
4599	c.4396A>T	rs1064794830	Pathogenic	Uncertain significance
4600	c.4393A>G		Pathogenic	Uncertain significance
4601	c.4390C>G		Pathogenic	Uncertain significance
4602	c.4390C>T	rs1259139517	Benign	Uncertain significance
4603	c.4390C>A	rs1259139517	Pathogenic	Uncertain significance
4604	c.4385A>T		Pathogenic	Uncertain significance
4605	c.4384G>A	rs141255461	Pathogenic	Uncertain significance
4606	c.4383T>A	rs431825408	Benign	Uncertain significance
4607	c.4382G>C	rs1555582692	Benign	Uncertain significance
4608	c.4379G>A	rs397509167	Pathogenic	Uncertain significance
4609	c.4376A>G	rs1555582697	Benign	Uncertain significance
4610	c.4370C>T		Benign	Uncertain significance
4611	c.4369T>A		Benign	Uncertain significance
4612	c.4367C>T		Benign	Uncertain significance
4613	c.4366A>G	rs786201835	Benign	Uncertain significance
4614	c.4360G>A	rs1555582714	Pathogenic	Uncertain significance
4615	c.4358C>T	rs1171055085	Benign	Uncertain significance
4616	c.4358-1G>T		Benign	Uncertain significance
4617	c.4358-6T>C	rs1135401875	Benign	Uncertain significance
4618	c.4357G>T	rs1555583984	Benign	Uncertain significance
4619	c.4351G>A	rs1555583989	Pathogenic	Uncertain significance
4620	c.4349C>T	rs886040226	Benign	Uncertain significance
4621	c.4344C>G		Benign	Uncertain significance
4622	c.4339C>A	rs80357067	Benign	Uncertain significance
4623	c.4338A>T	rs1555584018	Benign	Uncertain significance
4624	c.4337A>G	rs1273755215	Benign	Uncertain significance
4625	c.4337A>C	rs1273755215	Benign	Uncertain significance
4626	c.4333C>A	rs876660684	Benign	Uncertain significance
4627	c.4331A>T		Benign	Uncertain significance
4628	c.4319A>G	rs786202288	Pathogenic	Uncertain significance
4629	c.4318G>A		Pathogenic	Uncertain significance
4630	c.4318G>C	rs1555584070	Benign	Uncertain significance
4631	c.4312G>A		Pathogenic	Uncertain significance
4632	c.4310C>T	rs1555584083	Benign	Uncertain significance
4633	c.4305C>G	rs730881445	Benign	Uncertain significance
4634	c.4304A>T		Benign	Uncertain significance
4635	c.4304A>G	rs876660809	Benign	Uncertain significance
4636	c.4300A>G		Benign	Uncertain significance
4637	c.4297A>G	rs541512953	Pathogenic	Uncertain significance
4638	c.4292C>T	rs748839289	Benign	Uncertain significance
4639	c.4288C>G	rs80357466	Pathogenic	Uncertain significance
4640	c.4288C>T	rs80357466	Benign	Uncertain significance
4641	c.4286A>G	rs876659228	Pathogenic	Uncertain significance
4642	c.4273C>T	rs768327850	Benign	Uncertain significance
4643	c.4273C>G	rs768327850	Benign	Uncertain significance

4644	c.4272G>C	rs398122684	Benign	Uncertain significance
4645	c.4271A>G	rs1555584133	Pathogenic	Uncertain significance
4646	c.4268G>T	rs876660129	Benign	Uncertain significance
4647	c.4265G>A	rs747364414	Pathogenic	Uncertain significance
4648	c.4262A>T	rs80357079	Benign	Uncertain significance
4649	c.4260G>T	rs1567783343	Benign	Uncertain significance
4650	c.4258C>G	rs80357305	Benign	Uncertain significance
4651	c.4254A>T	rs1555584157	Pathogenic	Uncertain significance
4652	c.4253T>C	rs397509157	Benign	Uncertain significance
4653	c.4249G>A	rs1029805420	Pathogenic	Uncertain significance
4654	c.4247C>G		Benign	Uncertain significance
4655	c.4246G>A	rs370999077	Pathogenic	Uncertain significance
4656	c.4243G>C	rs1057519558	Benign	Uncertain significance
4657	c.4243G>A	rs1057519558	Pathogenic	Uncertain significance
4658	c.4241T>G	rs878854951	Benign	Uncertain significance
4659	c.4241T>C	rs878854951	Benign	Uncertain significance
4660	c.4237G>A		Pathogenic	Uncertain significance
4661	c.4234G>C		Benign	Uncertain significance
4662	c.4234G>A	rs765183110	Benign	Uncertain significance
4663	c.4234G>T	rs765183110	Benign	Uncertain significance
4664	c.4233G>A	rs1567783458	Pathogenic	Uncertain significance
4665	c.4231A>G	rs587781768	Benign	Uncertain significance
4666	c.4223A>C	rs1555584227	Benign	Uncertain significance
4667	c.4223A>G	rs1555584227	Pathogenic	Uncertain significance
4668	c.4220T>G	rs80357492	Benign	Uncertain significance
4669	c.4220T>C	rs80357492	Benign	Uncertain significance
4670	c.4219C>G	rs397507227	Benign	Uncertain significance
4671	c.4211T>G	rs80356916	Benign	Uncertain significance
4672	c.4211T>C	rs80356916	Benign	Uncertain significance
4673	c.4205A>C	rs80356882	Benign	Uncertain significance
4674	c.4205A>G	rs80356882	Benign	Uncertain significance
4675	c.4200G>C		Benign	Uncertain significance
4676	c.4199T>A		Benign	Uncertain significance
4677	c.4199T>C	rs80357473	Benign	Uncertain significance
4678	c.4198A>G	rs80357306	Benign	Uncertain significance
4679	c.4196C>G	rs876658465	Benign	Uncertain significance
4680	c.4193A>G	rs761640584	Benign	Uncertain significance
4681	c.4192G>A		Benign	Uncertain significance
4682	c.4192G>T	rs876660331	Pathogenic	Uncertain significance
4683	c.4190G>A	rs767421571	Pathogenic	Uncertain significance
4684	c.4189A>C	rs886037792	Benign	Uncertain significance
4685	c.4186C>A	rs80357011	Benign	Uncertain significance
4686	c.4180A>C	rs1555586062	Benign	Uncertain significance
4687	c.4178C>G	rs1555586071	Benign	Uncertain significance
4688	c.4177A>G	rs587782870	Benign	Uncertain significance
4689	c.4175T>C	rs778319854	Benign	Uncertain significance
4690	c.4172T>G	rs397509146	Benign	Uncertain significance
4691	c.4171A>G		Benign	Uncertain significance
4692	c.4169A>G	rs1165149350	Benign	Uncertain significance
4693	c.4168G>A	rs752300203	Pathogenic	Uncertain significance
4694	c.4159T>C	rs876658221	Benign	Uncertain significance
4695	c.4154T>C		Benign	Uncertain significance
4696	c.4151G>A	rs786203545	Benign	Uncertain significance
4697	c.4150G>A	rs1555586146	Pathogenic	Uncertain significance

4698	c.4148C>T	rs80357071	Benign	Uncertain significance
4699	c.4144T>A	rs786202106	Benign	Uncertain significance
4700	c.4143C>G	rs1567788391	Benign	Uncertain significance
4701	c.4133T>C	rs1567788422	Benign	Uncertain significance
4702	c.4132G>T	rs28897690	Benign	Uncertain significance
4703	c.4131C>A	rs80356871	Benign	Uncertain significance
4704	c.4130G>A	rs1567788442	Pathogenic	Uncertain significance
4705	c.4129A>G	rs730881491	Benign	Uncertain significance
4706	c.4126A>G		Benign	Uncertain significance
4707	c.4123G>A	rs80357397	Pathogenic	Uncertain significance
4708	c.4122T>A	rs1555586200	Benign	Uncertain significance
4709	c.4121G>A	rs1382449149	Pathogenic	Uncertain significance
4710	c.4111G>A	rs774593602	Pathogenic	Uncertain significance
4711	c.4105G>C		Benign	Uncertain significance
4712	c.4103C>T		Benign	Uncertain significance
4713	c.4102G>A	rs1064796463	Pathogenic	Uncertain significance
4714	c.4099G>A	rs786202998	Pathogenic	Uncertain significance
4715	c.4097G>A	rs876660948	Pathogenic	Uncertain significance
4716	c.4096+1G>T		Pathogenic	Uncertain significance
4717	c.4096G>C	rs431825405	Benign	Uncertain significance
4718	c.4096G>A	rs431825405	Pathogenic	Uncertain significance
4719	c.4088C>T	rs398122680	Benign	Uncertain significance
4720	c.4085A>T		Benign	Uncertain significance
4721	c.4084G>A	rs775463394	Pathogenic	Uncertain significance
4722	c.4083G>A	rs374192364	Pathogenic	Uncertain significance
4723	c.4082T>C		Benign	Uncertain significance
4724	c.4075C>A		Benign	Uncertain significance
4725	c.4075C>G	rs80357456	Benign	Uncertain significance
4726	c.4073A>G	rs397507225	Pathogenic	Uncertain significance
4727	c.4072G>A	rs397509136	Pathogenic	Uncertain significance
4728	c.4071A>T	rs786201475	Pathogenic	Uncertain significance
4729	c.4069G>C		Benign	Uncertain significance
4730	c.4064A>G	rs1567789081	Benign	Uncertain significance
4731	c.4063A>G	rs876660530	Pathogenic	Uncertain significance
4732	c.4061A>G	rs767246037	Benign	Uncertain significance
4733	c.4057G>A		Pathogenic	Uncertain significance
4734	c.4055A>T	rs879254228	Pathogenic	Uncertain significance
4735	c.4054G>C	rs80357202	Benign	Uncertain significance
4736	c.4054G>A	rs80357202	Pathogenic	Uncertain significance
4737	c.4049G>T	rs1433564897	Benign	Uncertain significance
4738	c.4048G>C	rs748674194	Benign	Uncertain significance
4739	c.4048G>T	rs748674194	Benign	Uncertain significance
4740	c.4046C>G	rs80357345	Benign	Uncertain significance
4741	c.4045A>G	rs80357231	Benign	Uncertain significance
4742	c.4045A>C	rs80357231	Benign	Uncertain significance
4743	c.4040G>A	rs80357210	Pathogenic	Uncertain significance
4744	c.4034A>T		Pathogenic	Uncertain significance
4745	c.4031A>G	rs55639854	Benign	Uncertain significance
4746	c.4028A>T	rs775339017	Benign	Uncertain significance
4747	c.4027G>T	rs1160287128	Pathogenic	Uncertain significance
4748	c.4021G>A	rs762908108	Pathogenic	Uncertain significance
4749	c.4020G>T	rs1555586637	Benign	Uncertain significance
4750	c.4016A>G	rs886039313	Benign	Uncertain significance
4751	c.4015G>A	rs80357021	Benign	Uncertain significance

4752	c.4013A>G	rs1555586661	Benign	Uncertain significance
4753	c.4011C>G	rs80356886	Benign	Uncertain significance
4754	c.4009G>C	rs886041144	Benign	Uncertain significance
4755	c.4006A>T	rs976725810	Pathogenic	Uncertain significance
4756	c.3998T>C	rs1135401872	Benign	Uncertain significance
4757	c.3996A>G	rs1567789328	Pathogenic	Uncertain significance
4758	c.3995G>T	rs730881490	Pathogenic	Uncertain significance
4759	c.3992A>G	rs1060502363	Pathogenic	Uncertain significance
4760	c.3988A>T	rs1555586688	Pathogenic	Uncertain significance
4761	c.3985G>A	rs876659467	Pathogenic	Uncertain significance
4762	c.3980A>G	rs730881444	Pathogenic	Uncertain significance
4763	c.3979C>G		Pathogenic	Uncertain significance
4764	c.3976C>T	rs1567789376	Benign	Uncertain significance
4765	c.3974G>A	rs921253348	Pathogenic	Uncertain significance
4766	c.3971T>C		Benign	Uncertain significance
4767	c.3970A>T	rs587782241	Benign	Uncertain significance
4768	c.3969A>T	rs876660410	Pathogenic	Uncertain significance
4769	c.3965A>G	rs80357042	Benign	Uncertain significance
4770	c.3965A>C	rs80357042	Benign	Uncertain significance
4771	c.3965A>T	rs80357042	Pathogenic	Uncertain significance
4772	c.3964A>G	rs80357343	Benign	Uncertain significance
4773	c.3964A>C	rs80357343	Benign	Uncertain significance
4774	c.3962C>A	rs386833394	Benign	Uncertain significance
4775	c.3962C>G	rs386833394	Benign	Uncertain significance
4776	c.3955G>A	rs431825403	Pathogenic	Uncertain significance
4777	c.3953T>C	rs1388475291	Benign	Uncertain significance
4778	c.3952A>G	rs397509121	Benign	Uncertain significance
4779	c.3944C>A	rs80357500	Benign	Uncertain significance
4780	c.3941A>T	rs759916956	Benign	Uncertain significance
4781	c.3940G>A	rs80356954	Benign	Uncertain significance
4782	c.3938A>G	rs765729710	Pathogenic	Uncertain significance
4783	c.3933C>A		Benign	Uncertain significance
4784	c.3931A>G	rs864622233	Benign	Uncertain significance
4785	c.3929C>T	rs80357257	Benign	Uncertain significance
4786	c.3929C>G	rs80357257	Benign	Uncertain significance
4787	c.3918G>C	rs786202068	Benign	Uncertain significance
4788	c.3914A>T	rs431825402	Pathogenic	Uncertain significance
4789	c.3913G>C	rs1555586799	Benign	Uncertain significance
4790	c.3906A>T	rs1567789643	Pathogenic	Uncertain significance
4791	c.3904G>A	rs80357461	Pathogenic	Uncertain significance
4792	c.3903T>A	rs273900719	Benign	Uncertain significance
4793	c.3902G>A	rs1057519496	Pathogenic	Uncertain significance
4794	c.3901A>T	rs786203580	Pathogenic	Uncertain significance
4795	c.3901A>G	rs786203580	Pathogenic	Uncertain significance
4796	c.3897G>T	rs398122678	Benign	Uncertain significance
4797	c.3896A>G	rs876660866	Pathogenic	Uncertain significance
4798	c.3893C>T	rs80357440	Benign	Uncertain significance
4799	c.3892T>A		Benign	Uncertain significance
4800	c.3889T>A	rs1450793674	Benign	Uncertain significance
4801	c.3889T>C	rs1450793674	Benign	Uncertain significance
4802	c.3887T>G	rs1555586855	Benign	Uncertain significance
4803	c.3886T>C		Benign	Uncertain significance
4804	c.3881G>T		Benign	Uncertain significance
4805	c.3878C>T	rs80357213	Benign	Uncertain significance

4806	c.3878C>A	rs80357213	Benign	Uncertain significance
4807	c.3877G>C	rs397507223	Benign	Uncertain significance
4808	c.3875C>G	rs876658340	Pathogenic	Uncertain significance
4809	c.3875C>A	rs876658340	Pathogenic	Uncertain significance
4810	c.3874T>C	rs786203823	Benign	Uncertain significance
4811	c.3869A>C	rs431825401	Benign	Uncertain significance
4812	c.3868A>G	rs80357254	Benign	Uncertain significance
4813	c.3865A>C	rs1064795449	Benign	Uncertain significance
4814	c.3862G>C	rs876659708	Benign	Uncertain significance
4815	c.3859G>A	rs1555586934	Pathogenic	Uncertain significance
4816	c.3857G>C	rs142383077	Benign	Uncertain significance
4817	c.3852C>A		Benign	Uncertain significance
4818	c.3851A>T		Benign	Uncertain significance
4819	c.3851A>G	rs80357499	Pathogenic	Uncertain significance
4820	c.3847C>T	rs1555586946	Benign	Uncertain significance
4821	c.3843G>T		Benign	Uncertain significance
4822	c.3842A>G		Pathogenic	Uncertain significance
4823	c.3842A>C	rs80357483	Pathogenic	Uncertain significance
4824	c.3841C>G	rs80356866	Pathogenic	Uncertain significance
4825	c.3839C>A		Pathogenic	Uncertain significance
4826	c.3838T>C	rs1567789887	Benign	Uncertain significance
4827	c.3835G>C	rs80357036	Benign	Uncertain significance
4828	c.3835G>T	rs80357036	Benign	Uncertain significance
4829	c.3831A>G	rs1567789910	Benign	Uncertain significance
4830	c.3830C>T	rs730881489	Pathogenic	Uncertain significance
4831	c.3824T>A		Benign	Uncertain significance
4832	c.3820G>C		Pathogenic	Uncertain significance
4833	c.3819G>C		Benign	Uncertain significance
4834	c.3818A>T		Pathogenic	Uncertain significance
4835	c.3816C>A	rs786201893	Benign	Uncertain significance
4836	c.3812G>C	rs1567789989	Benign	Uncertain significance
4837	c.3809G>A	rs1207787135	Pathogenic	Uncertain significance
4838	c.3807C>G	rs786202569	Benign	Uncertain significance
4839	c.3804T>G	rs140588714	Benign	Uncertain significance
4840	c.3803A>G	rs273900716	Benign	Uncertain significance
4841	c.3800T>C	rs587782190	Benign	Uncertain significance
4842	c.3798C>A	rs200648498	Benign	Uncertain significance
4843	c.3798C>G	rs200648498	Benign	Uncertain significance
4844	c.3797G>T	rs80357160	Benign	Uncertain significance
4845	c.3793A>C	rs1060502364	Benign	Uncertain significance
4846	c.3792G>C		Benign	Uncertain significance
4847	c.3791A>G	rs1555587039	Benign	Uncertain significance
4848	c.3785C>T	rs80357269	Benign	Uncertain significance
4849	c.3784T>C	rs1011096937	Benign	Uncertain significance
4850	c.3783A>T	rs80356831	Benign	Uncertain significance
4851	c.3782T>C	rs397507219	Benign	Uncertain significance
4852	c.3778T>G	rs1555587079	Benign	Uncertain significance
4853	c.3775A>G		Benign	Uncertain significance
4854	c.3774G>T	rs431825399	Pathogenic	Uncertain significance
4855	c.3772G>A	rs397509105	Pathogenic	Uncertain significance
4856	c.3770A>G		Pathogenic	Uncertain significance
4857	c.3769G>A	rs1567790183	Pathogenic	Uncertain significance
4858	c.3760A>G	rs80357362	Pathogenic	Uncertain significance
4859	c.3758C>G	rs397509100	Benign	Uncertain significance

4860	c.3755T>C	rs1555587147	Benign	Uncertain significance
4861	c.3753T>G	rs397509098	Benign	Uncertain significance
4862	c.3752G>A	rs879254079	Pathogenic	Uncertain significance
4863	c.3750G>T	rs145903082	Benign	Uncertain significance
4864	c.3746C>G	rs80357099	Benign	Uncertain significance
4865	c.3745A>G	rs1555587189	Pathogenic	Uncertain significance
4866	c.3740T>C		Benign	Uncertain significance
4867	c.3737C>T		Benign	Uncertain significance
4868	c.3736A>G	rs587776488	Benign	Uncertain significance
4869	c.3734G>A	rs1060502348	Pathogenic	Uncertain significance
4870	c.3728G>A		Pathogenic	Uncertain significance
4871	c.3720G>C	rs876658341	Benign	Uncertain significance
4872	c.3713C>G	rs28897688	Benign	Uncertain significance
4873	c.3710T>C	rs876660883	Benign	Uncertain significance
4874	c.3706A>G	rs1567790509	Benign	Uncertain significance
4875	c.3700G>C	rs763354142	Benign	Uncertain significance
4876	c.3698A>G	rs80357141	Benign	Uncertain significance
4877	c.3688T>A		Benign	Uncertain significance
4878	c.3682C>T	rs1567790599	Benign	Uncertain significance
4879	c.3681A>T	rs730881488	Benign	Uncertain significance
4880	c.3674G>C	rs1567790638	Benign	Uncertain significance
4881	c.3673T>G	rs1382025345	Benign	Uncertain significance
4882	c.3667C>T	rs1555587309	Benign	Uncertain significance
4883	c.3666G>C	rs1555587312	Benign	Uncertain significance
4884	c.3662A>C	rs273900713	Benign	Uncertain significance
4885	c.3659A>T	rs766572561	Benign	Uncertain significance
4886	c.3655G>A	rs80356921	Pathogenic	Uncertain significance
4887	c.3652A>T	rs80356894	Pathogenic	Uncertain significance
4888	c.3652A>G	rs80356894	Pathogenic	Uncertain significance
4889	c.3650C>G	rs398122676	Benign	Uncertain significance
4890	c.3649T>C	rs273900712	Benign	Uncertain significance
4891	c.3644A>G	rs786203310	Benign	Uncertain significance
4892	c.3642G>C	rs398122675	Benign	Uncertain significance
4893	c.3642G>T	rs398122675	Pathogenic	Uncertain significance
4894	c.3634T>C		Benign	Uncertain significance
4895	c.3632C>G	rs1555587377	Pathogenic	Uncertain significance
4896	c.3629A>G	rs1060502347	Pathogenic	Uncertain significance
4897	c.3625T>G	rs273900711	Benign	Uncertain significance
4898	c.3622A>G	rs80357152	Benign	Uncertain significance
4899	c.3620A>T	rs1555587402	Pathogenic	Uncertain significance
4900	c.3619A>G	rs80357455	Benign	Uncertain significance
4901	c.3616G>T	rs1555587407	Pathogenic	Uncertain significance
4902	c.3614G>T		Benign	Uncertain significance
4903	c.3613G>A	rs80357294	Pathogenic	Uncertain significance
4904	c.3611G>C	rs1456509049	Benign	Uncertain significance
4905	c.3607C>G	rs62625308	Benign	Uncertain significance
4906	c.3602G>A	rs1567790971	Pathogenic	Uncertain significance
4907	c.3601G>C	rs55725337	Pathogenic	Uncertain significance
4908	c.3595G>T	rs1555587437	Benign	Uncertain significance
4909	c.3587C>T	rs80356944	Benign	Uncertain significance
4910	c.3587C>A	rs80356944	Benign	Uncertain significance
4911	c.3586A>T	rs1340335862	Benign	Uncertain significance
4912	c.3584A>G	rs28897685	Benign	Uncertain significance
4913	c.3583C>G	rs876659903	Benign	Uncertain significance

4914	c.3583C>T	rs876659903	Benign	Uncertain significance
4915	c.3581C>T	rs80357290	Benign	Uncertain significance
4916	c.3580A>G	rs369982706	Pathogenic	Uncertain significance
4917	c.3572G>A	rs878854948	Pathogenic	Uncertain significance
4918	c.3569C>T	rs755209182	Benign	Uncertain significance
4919	c.3565A>T	rs1567791142	Pathogenic	Uncertain significance
4920	c.3564G>C	rs879255484	Benign	Uncertain significance
4921	c.3560G>C	rs80356975	Benign	Uncertain significance
4922	c.3557T>A	rs1567791164	Pathogenic	Uncertain significance
4923	c.3556C>T	rs1555587505	Benign	Uncertain significance
4924	c.3555G>T	rs587779368	Benign	Uncertain significance
4925	c.3548A>T	rs16942	Pathogenic	Uncertain significance
4926	c.3545A>T	rs1555587532	Pathogenic	Uncertain significance
4927	c.3542T>G		Benign	Uncertain significance
4928	c.3542T>C	rs80357032	Benign	Uncertain significance
4929	c.3541G>T		Benign	Uncertain significance
4930	c.3540C>G		Benign	Uncertain significance
4931	c.3539G>A	rs1064793057	Pathogenic	Uncertain significance
4932	c.3535A>C	rs587782188	Benign	Uncertain significance
4933	c.3533G>C	rs1294360179	Benign	Uncertain significance
4934	c.3533G>A	rs1294360179	Pathogenic	Uncertain significance
4935	c.3532A>G	rs1567791298	Pathogenic	Uncertain significance
4936	c.3527T>A	rs80357027	Benign	Uncertain significance
4937	c.3526G>A	rs777796838	Pathogenic	Uncertain significance
4938	c.3524C>T	rs1567791322	Benign	Uncertain significance
4939	c.3523G>A		Pathogenic	Uncertain significance
4940	c.3521C>G		Pathogenic	Uncertain significance
4941	c.3518G>T		Benign	Uncertain significance
4942	c.3518G>A	rs746949187	Benign	Uncertain significance
4943	c.3515A>G	rs80357206	Benign	Uncertain significance
4944	c.3511A>G	rs730882164	Pathogenic	Uncertain significance
4945	c.3510T>G		Pathogenic	Uncertain significance
4946	c.3508A>G	rs273899708	Benign	Uncertain significance
4947	c.3508A>T	rs273899708	Benign	Uncertain significance
4948	c.3506A>G	rs1323940169	Benign	Uncertain significance
4949	c.3505G>A		Pathogenic	Uncertain significance
4950	c.3505G>T	rs876659269	Pathogenic	Uncertain significance
4951	c.3504T>G	rs1458197554	Benign	Uncertain significance
4952	c.3496G>C	rs745418679	Benign	Uncertain significance
4953	c.3491G>T	rs397509075	Benign	Uncertain significance
4954	c.3488C>T	rs80356918	Benign	Uncertain significance
4955	c.3487A>G	rs769456095	Pathogenic	Uncertain significance
4956	c.3484G>T	rs1555587619	Pathogenic	Uncertain significance
4957	c.3481G>A		Benign	Uncertain significance
4958	c.3479A>C		Benign	Uncertain significance
4959	c.3476T>A	rs1567791496	Benign	Uncertain significance
4960	c.3475A>G	rs876658318	Benign	Uncertain significance
4961	c.3472G>A		Pathogenic	Uncertain significance
4962	c.3470G>C	rs876659133	Benign	Uncertain significance
4963	c.3466G>A	rs1064793302	Pathogenic	Uncertain significance
4964	c.3460T>A	rs1567791553	Benign	Uncertain significance
4965	c.3458T>C		Benign	Uncertain significance
4966	c.3457C>G	rs1555587658	Benign	Uncertain significance
4967	c.3454G>C	rs80357175	Benign	Uncertain significance

4968	c.3449C>T	rs587782752	Benign	Uncertain significance
4969	c.3440C>A	rs876660757	Pathogenic	Uncertain significance
4970	c.3437G>A	rs80357247	Pathogenic	Uncertain significance
4971	c.3437G>C	rs80357247	Benign	Uncertain significance
4972	c.3436T>G	rs1567791612	Benign	Uncertain significance
4973	c.3433G>A	rs431825396	Benign	Uncertain significance
4974	c.3433G>T	rs431825396	Pathogenic	Uncertain significance
4975	c.3432G>T	rs80356922	Benign	Uncertain significance
4976	c.3428C>G	rs80357434	Pathogenic	Uncertain significance
4977	c.3428C>T	rs80357434	Benign	Uncertain significance
4978	c.3425C>T	rs1555587711	Benign	Uncertain significance
4979	c.3424G>T	rs80357101	Benign	Uncertain significance
4980	c.3424G>C	rs80357101	Benign	Uncertain significance
4981	c.3421C>T	rs1555587727	Benign	Uncertain significance
4982	c.3419G>A		Pathogenic	Uncertain significance
4983	c.3418A>C	rs2227945	Benign	Uncertain significance
4984	c.3415A>C	rs587781765	Benign	Uncertain significance
4985	c.3411G>A	rs786202900	Pathogenic	Uncertain significance
4986	c.3409A>G	rs771479616	Pathogenic	Uncertain significance
4987	c.3407C>G	rs80357329	Benign	Uncertain significance
4988	c.3406C>T		Benign	Uncertain significance
4989	c.3406C>A	rs431825395	Benign	Uncertain significance
4990	c.3403C>G	rs80357136	Benign	Uncertain significance
4991	c.3401A>T	rs762744684	Pathogenic	Uncertain significance
4992	c.3400G>A	rs80357018	Pathogenic	Uncertain significance
4993	c.3398T>C	rs80356971	Benign	Uncertain significance
4994	c.3394A>G	rs530464947	Pathogenic	Uncertain significance
4995	c.3392A>T	rs1555587813	Benign	Uncertain significance
4996	c.3392A>G	rs1555587813	Benign	Uncertain significance
4997	c.3391G>A	rs1555587816	Pathogenic	Uncertain significance
4998	c.3386T>C	rs1567791871	Benign	Uncertain significance
4999	c.3385A>G		Benign	Uncertain significance
5000	c.3383T>C	rs1555587827	Benign	Uncertain significance
5001	c.3379T>C	rs1451089848	Benign	Uncertain significance
5002	c.3377C>T	rs80356887	Benign	Uncertain significance
5003	c.3374C>A	rs1555587835	Pathogenic	Uncertain significance
5004	c.3373T>C	rs1555587839	Benign	Uncertain significance
5005	c.3368A>T	rs1555587851	Pathogenic	Uncertain significance
5006	c.3366A>C	rs1567791947	Benign	Uncertain significance
5007	c.3361A>G	rs876660526	Pathogenic	Uncertain significance
5008	c.3361A>C	rs876660526	Benign	Uncertain significance
5009	c.3359T>A	rs1361427704	Benign	Uncertain significance
5010	c.3358G>C		Benign	Uncertain significance
5011	c.3358G>A	rs748894760	Pathogenic	Uncertain significance
5012	c.3356C>G	rs863224759	Benign	Uncertain significance
5013	c.3354G>T	rs80357334	Pathogenic	Uncertain significance
5014	c.3347T>G	rs786203153	Benign	Uncertain significance
5015	c.3345A>C	rs876658243	Benign	Uncertain significance
5016	c.3344A>T	rs1555587933	Pathogenic	Uncertain significance
5017	c.3338A>G		Pathogenic	Uncertain significance
5018	c.3331C>G		Benign	Uncertain significance
5019	c.3320A>G		Benign	Uncertain significance
5020	c.3316C>T	rs45599040	Benign	Uncertain significance
5021	c.3315T>A		Benign	Uncertain significance

5022	c.3313C>A	rs80357288	Benign	Uncertain significance
5023	c.3312G>C		Benign	Uncertain significance
5024	c.3309T>C	rs80357317	Benign	Uncertain significance
5025	c.3308G>A	rs80357135	Pathogenic	Uncertain significance
5026	c.3308G>T	rs80357135	Benign	Uncertain significance
5027	c.3305A>G	rs80356900	Benign	Uncertain significance
5028	c.3301A>G		Benign	Uncertain significance
5029	c.3298G>A	rs1555587999	Pathogenic	Uncertain significance
5030	c.3292C>T	rs879255291	Benign	Uncertain significance
5031	c.3289A>C		Benign	Uncertain significance
5032	c.3289A>T	rs587782864	Pathogenic	Uncertain significance
5033	c.3288A>G		Benign	Uncertain significance
5034	c.3287A>G	rs273899704	Benign	Uncertain significance
5035	c.3286C>A	rs80357485	Benign	Uncertain significance
5036	c.3281A>G		Pathogenic	Uncertain significance
5037	c.3280T>G		Benign	Uncertain significance
5038	c.3280T>C	rs1555588026	Benign	Uncertain significance
5039	c.3277G>T	rs587778117	Pathogenic	Uncertain significance
5040	c.3276G>T	rs764458412	Benign	Uncertain significance
5041	c.3274G>A	rs876658360	Pathogenic	Uncertain significance
5042	c.3272C>T		Benign	Uncertain significance
5043	c.3269A>G	rs1555588045	Benign	Uncertain significance
5044	c.3267G>T	rs767544239	Benign	Uncertain significance
5045	c.3263T>A	rs80356901	Benign	Uncertain significance
5046	c.3260G>C	rs80357172	Pathogenic	Uncertain significance
5047	c.3257T>C	rs80357006	Benign	Uncertain significance
5048	c.3250C>A	rs879254009	Benign	Uncertain significance
5049	c.3248T>C	rs786203958	Benign	Uncertain significance
5050	c.3247A>C	rs397507213	Benign	Uncertain significance
5051	c.3245C>T	rs1555588115	Benign	Uncertain significance
5052	c.3244G>T		Benign	Uncertain significance
5053	c.3244G>A	rs779459487	Pathogenic	Uncertain significance
5054	c.3242A>G		Benign	Uncertain significance
5055	c.3241A>G	rs876659928	Benign	Uncertain significance
5056	c.3238T>G	rs754597283	Benign	Uncertain significance
5057	c.3230G>A	rs1567792424	Benign	Uncertain significance
5058	c.3227G>T	rs80357313	Pathogenic	Uncertain significance
5059	c.3227G>C	rs80357313	Benign	Uncertain significance
5060	c.3225C>G	rs778607600	Benign	Uncertain significance
5061	c.3223A>G	rs1567792459	Benign	Uncertain significance
5062	c.3221G>C	rs786202155	Benign	Uncertain significance
5063	c.3220A>G	rs80357263	Pathogenic	Uncertain significance
5064	c.3218G>A	rs757632961	Pathogenic	Uncertain significance
5065	c.3217G>A	rs878854945	Pathogenic	Uncertain significance
5066	c.3209C>G	rs397507212	Benign	Uncertain significance
5067	c.3207A>T	rs1567792523	Pathogenic	Uncertain significance
5068	c.3206A>T	rs879254151	Pathogenic	Uncertain significance
5069	c.3200A>G	rs1555588214	Benign	Uncertain significance
5070	c.3199A>T		Pathogenic	Uncertain significance
5071	c.3196G>A		Pathogenic	Uncertain significance
5072	c.3195T>G	rs1567792553	Benign	Uncertain significance
5073	c.3194A>T	rs1555588228	Benign	Uncertain significance
5074	c.3190A>T	rs273899702	Pathogenic	Uncertain significance
5075	c.3185G>T	rs397507211	Pathogenic	Uncertain significance

5076	c.3184G>A	rs1567792594	Pathogenic	Uncertain significance
5077	c.3182T>C		Benign	Uncertain significance
5078	c.3181A>C	rs876658975	Benign	Uncertain significance
5079	c.3178G>C	rs80357424	Benign	Uncertain significance
5080	c.3173T>G	rs1555588264	Benign	Uncertain significance
5081	c.3172A>G	rs1064795478	Pathogenic	Uncertain significance
5082	c.3170G>C		Benign	Uncertain significance
5083	c.3170G>A	rs587776487	Pathogenic	Uncertain significance
5084	c.3169A>C	rs80357479	Benign	Uncertain significance
5085	c.3169A>G	rs80357479	Pathogenic	Uncertain significance
5086	c.3167C>T	rs587781588	Benign	Uncertain significance
5087	c.3167C>G	rs587781588	Benign	Uncertain significance
5088	c.3166T>C	rs876659601	Benign	Uncertain significance
5089	c.3163G>A	rs749417532	Pathogenic	Uncertain significance
5090	c.3160G>A	rs876658479	Pathogenic	Uncertain significance
5091	c.3159A>G		Benign	Uncertain significance
5092	c.3159A>C	rs1567792698	Benign	Uncertain significance
5093	c.3155A>G	rs398122672	Benign	Uncertain significance
5094	c.3154A>G	rs768995134	Pathogenic	Uncertain significance
5095	c.3152C>T	rs397509039	Benign	Uncertain significance
5096	c.3151A>G	rs398122671	Pathogenic	Uncertain significance
5097	c.3150T>G	rs1555588305	Benign	Uncertain significance
5098	c.3149G>C		Benign	Uncertain significance
5099	c.3148A>G		Pathogenic	Uncertain significance
5100	c.3143G>A	rs80356899	Benign	Uncertain significance
5101	c.3139G>A		Pathogenic	Uncertain significance
5102	c.3136G>C	rs1555588329	Benign	Uncertain significance
5103	c.3134A>G	rs1555588330	Benign	Uncertain significance
5104	c.3132T>G	rs1555588333	Pathogenic	Uncertain significance
5105	c.3131T>G		Benign	Uncertain significance
5106	c.3128A>C	rs1228972723	Benign	Uncertain significance
5107	c.3127A>C	rs587782630	Benign	Uncertain significance
5108	c.3127A>G	rs587782630	Benign	Uncertain significance
5109	c.3126C>G	rs878854943	Benign	Uncertain significance
5110	c.3124A>C		Benign	Uncertain significance
5111	c.3122C>T	rs397509035	Benign	Uncertain significance
5112	c.3120C>T	rs1064794877	Benign	Uncertain significance
5113	c.3119G>C	rs4986852	Benign	Uncertain significance
5114	c.3116C>T	rs1173254670	Benign	Uncertain significance
5115	c.3106T>C	rs766381694	Benign	Uncertain significance
5116	c.3104T>C	rs1555588389	Benign	Uncertain significance
5117	c.3097G>A	rs273899698	Pathogenic	Uncertain significance
5118	c.3093T>C		Benign	Uncertain significance
5119	c.3092T>G	rs863224758	Benign	Uncertain significance
5120	c.3091A>G	rs786203979	Benign	Uncertain significance
5121	c.3091A>T	rs786203979	Benign	Uncertain significance
5122	c.3086A>G	rs753286589	Benign	Uncertain significance
5123	c.3083G>T	rs80357459	Benign	Uncertain significance
5124	c.3080G>C	rs80357386	Benign	Uncertain significance
5125	c.3080G>A	rs80357386	Pathogenic	Uncertain significance
5126	c.3078T>G	rs1555588422	Pathogenic	Uncertain significance
5127	c.3071G>C	rs757579891	Benign	Uncertain significance
5128	c.3071G>A	rs757579891	Pathogenic	Uncertain significance
5129	c.3065C>T	rs786202070	Benign	Uncertain significance

5130	c.3053A>G	rs1567793093	Benign	Uncertain significance
5131	c.3048T>G	rs879255482	Benign	Uncertain significance
5132	c.3047A>G	rs1567793123	Benign	Uncertain significance
5133	c.3046A>G	rs80357154	Benign	Uncertain significance
5134	c.3042G>A	rs1555588493	Pathogenic	Uncertain significance
5135	c.3041T>A	rs80357020	Benign	Uncertain significance
5136	c.3040A>T	rs80356933	Benign	Uncertain significance
5137	c.3040A>G	rs80356933	Benign	Uncertain significance
5138	c.3037G>A	rs1555588500	Pathogenic	Uncertain significance
5139	c.3036A>C	rs1567793150	Benign	Uncertain significance
5140	c.3035G>C		Benign	Uncertain significance
5141	c.3035G>A	rs876658464	Pathogenic	Uncertain significance
5142	c.3034A>G	rs1555588503	Benign	Uncertain significance
5143	c.3032A>G		Benign	Uncertain significance
5144	c.3031G>A	rs876659974	Pathogenic	Uncertain significance
5145	c.3029C>T		Benign	Uncertain significance
5146	c.3028C>T	rs1057519249	Benign	Uncertain significance
5147	c.3024G>T		Benign	Uncertain significance
5148	c.3022A>C	rs56321129	Benign	Uncertain significance
5149	c.3010G>C	rs786202534	Benign	Uncertain significance
5150	c.3005A>T	rs1555588553	Benign	Uncertain significance
5151	c.3004A>G	rs786202665	Benign	Uncertain significance
5152	c.3001G>A		Benign	Uncertain significance
5153	c.3000G>C	rs748410422	Benign	Uncertain significance
5154	c.2999A>G	rs1060502330	Pathogenic	Uncertain significance
5155	c.2998G>A	rs80357124	Pathogenic	Uncertain significance
5156	c.2996T>G	rs876659514	Benign	Uncertain significance
5157	c.2995C>A	rs80356848	Benign	Uncertain significance
5158	c.2993T>G		Benign	Uncertain significance
5159	c.2993T>A	rs1567793330	Benign	Uncertain significance
5160	c.2992C>G	rs876659077	Pathogenic	Uncertain significance
5161	c.2990A>G		Benign	Uncertain significance
5162	c.2985G>T	rs1555588591	Pathogenic	Uncertain significance
5163	c.2983A>C		Benign	Uncertain significance
5164	c.2981G>T		Benign	Uncertain significance
5165	c.2981G>A	rs1238452758	Pathogenic	Uncertain significance
5166	c.2980T>C	rs144853230	Benign	Uncertain significance
5167	c.2980T>A	rs144853230	Benign	Uncertain significance
5168	c.2973A>C	rs876660683	Benign	Uncertain significance
5169	c.2969T>C	rs760588785	Benign	Uncertain significance
5170	c.2968G>A	rs397509029	Pathogenic	Uncertain significance
5171	c.2967T>G		Benign	Uncertain significance
5172	c.2967T>A	rs876659270	Pathogenic	Uncertain significance
5173	c.2960A>G	rs876659990	Benign	Uncertain significance
5174	c.2959A>G	rs878854941	Pathogenic	Uncertain significance
5175	c.2953C>T	rs1555588649	Benign	Uncertain significance
5176	c.2950T>C		Benign	Uncertain significance
5177	c.2942C>A	rs876659025	Pathogenic	Uncertain significance
5178	c.2941C>A	rs1555588677	Pathogenic	Uncertain significance
5179	c.2936G>T		Benign	Uncertain significance
5180	c.2935C>A	rs80356970	Benign	Uncertain significance
5181	c.2933A>G	rs863224756	Pathogenic	Uncertain significance
5182	c.2932T>C	rs876659545	Benign	Uncertain significance
5183	c.2930C>T	rs141465583	Benign	Uncertain significance

5184	c.2929C>T	rs1064794043	Benign	Uncertain significance
5185	c.2922A>C	rs730881487	Benign	Uncertain significance
5186	c.2921T>C	rs80356872	Benign	Uncertain significance
5187	c.2917C>A	rs80357080	Benign	Uncertain significance
5188	c.2917C>G	rs80357080	Benign	Uncertain significance
5189	c.2915G>A	rs587782721	Pathogenic	Uncertain significance
5190	c.2913T>G		Benign	Uncertain significance
5191	c.2911C>G	rs80357478	Benign	Uncertain significance
5192	c.2911C>A	rs80357478	Benign	Uncertain significance
5193	c.2909A>G		Benign	Uncertain significance
5194	c.2909A>T	rs756559408	Pathogenic	Uncertain significance
5195	c.2908A>G	rs1567793674	Pathogenic	Uncertain significance
5196	c.2906A>G	rs1567793695	Benign	Uncertain significance
5197	c.2905A>G	rs587781641	Benign	Uncertain significance
5198	c.2902C>T		Benign	Uncertain significance
5199	c.2899A>T	rs273899690	Benign	Uncertain significance
5200	c.2897T>C	rs879254045	Benign	Uncertain significance
5201	c.2894T>G	rs876660682	Benign	Uncertain significance
5202	c.2893C>T		Benign	Uncertain significance
5203	c.2893C>G	rs1060502344	Benign	Uncertain significance
5204	c.2890G>A	rs879254027	Pathogenic	Uncertain significance
5205	c.2888C>T	rs730881443	Benign	Uncertain significance
5206	c.2885A>G	rs780367532	Benign	Uncertain significance
5207	c.2882A>G	rs879254130	Benign	Uncertain significance
5208	c.2881A>T		Pathogenic	Uncertain significance
5209	c.2881A>C	rs786203786	Benign	Uncertain significance
5210	c.2878G>A	rs1555588796	Pathogenic	Uncertain significance
5211	c.2877A>C	rs587782743	Benign	Uncertain significance
5212	c.2875A>G		Pathogenic	Uncertain significance
5213	c.2872T>C	rs80356878	Benign	Uncertain significance
5214	c.2872T>A	rs80356878	Benign	Uncertain significance
5215	c.2870A>C	rs1567793946	Benign	Uncertain significance
5216	c.2867C>G	rs1060502336	Pathogenic	Uncertain significance
5217	c.2857T>C	rs1555588840	Benign	Uncertain significance
5218	c.2851A>G		Pathogenic	Uncertain significance
5219	c.2849C>T	rs1555588847	Benign	Uncertain significance
5220	c.2846G>A	rs730881486	Benign	Uncertain significance
5221	c.2845G>A	rs1324818767	Pathogenic	Uncertain significance
5222	c.2841A>T	rs864622618	Pathogenic	Uncertain significance
5223	c.2840A>G	rs778118145	Benign	Uncertain significance
5224	c.2836A>G		Pathogenic	Uncertain significance
5225	c.2836A>T	rs876660901	Benign	Uncertain significance
5226	c.2833A>G	rs1567794085	Pathogenic	Uncertain significance
5227	c.2831G>A	rs770769275	Pathogenic	Uncertain significance
5228	c.2830T>A	rs1064795603	Benign	Uncertain significance
5229	c.2823T>G		Benign	Uncertain significance
5230	c.2822A>G	rs776512377	Benign	Uncertain significance
5231	c.2818G>C	rs80357077	Benign	Uncertain significance
5232	c.2818G>T	rs80357077	Pathogenic	Uncertain significance
5233	c.2816T>C	rs1555588894	Benign	Uncertain significance
5234	c.2815G>A	rs1555588896	Benign	Uncertain significance
5235	c.2813C>T	rs1064793999	Benign	Uncertain significance
5236	c.2812C>T		Benign	Uncertain significance
5237	c.2808T>G	rs730881485	Benign	Uncertain significance

5238	c.2798G>A	rs80356941	Pathogenic	Uncertain significance
5239	c.2794G>T		Benign	Uncertain significance
5240	c.2791G>A	rs763639161	Pathogenic	Uncertain significance
5241	c.2791G>T	rs763639161	Benign	Uncertain significance
5242	c.2789C>T	rs80357256	Benign	Uncertain significance
5243	c.2783G>A	rs202004680	Benign	Uncertain significance
5244	c.2782G>A	rs80356995	Pathogenic	Uncertain significance
5245	c.2774T>C	rs1567794296	Benign	Uncertain significance
5246	c.2768T>C		Benign	Uncertain significance
5247	c.2765C>T	rs80357460	Benign	Uncertain significance
5248	c.2765C>G	rs80357460	Benign	Uncertain significance
5249	c.2764A>G	rs1555588988	Benign	Uncertain significance
5250	c.2762A>G	rs1555588991	Pathogenic	Uncertain significance
5251	c.2759T>C	rs80357008	Benign	Uncertain significance
5252	c.2758G>T	rs80357361	Benign	Uncertain significance
5253	c.2754G>T	rs398122668	Benign	Uncertain significance
5254	c.2752A>C	rs397509010	Benign	Uncertain significance
5255	c.2751C>G		Pathogenic	Uncertain significance
5256	c.2750T>G	rs587781492	Benign	Uncertain significance
5257	c.2750T>C	rs587781492	Benign	Uncertain significance
5258	c.2747A>G	rs864622588	Benign	Uncertain significance
5259	c.2747A>T	rs864622588	Pathogenic	Uncertain significance
5260	c.2746A>G	rs398122666	Pathogenic	Uncertain significance
5261	c.2746A>T	rs398122666	Pathogenic	Uncertain significance
5262	c.2740G>A	rs80357419	Pathogenic	Uncertain significance
5263	c.2739T>A	rs273899688	Benign	Uncertain significance
5264	c.2738A>G	rs199954851	Benign	Uncertain significance
5265	c.2737A>G		Benign	Uncertain significance
5266	c.2736G>T	rs1555589046	Pathogenic	Uncertain significance
5267	c.2736G>C	rs1555589046	Benign	Uncertain significance
5268	c.2734A>C	rs1555589048	Benign	Uncertain significance
5269	c.2732G>A	rs431825392	Benign	Uncertain significance
5270	c.2728C>G	rs397509004	Pathogenic	Uncertain significance
5271	c.2724A>C	rs1350643283	Benign	Uncertain significance
5272	c.2723A>C	rs1064794969	Benign	Uncertain significance
5273	c.2723A>G	rs1064794969	Benign	Uncertain significance
5274	c.2720A>G	rs1555589078	Benign	Uncertain significance
5275	c.2716A>C	rs1567794535	Benign	Uncertain significance
5276	c.2714A>G	rs397507203	Benign	Uncertain significance
5277	c.2710G>C	rs80357035	Benign	Uncertain significance
5278	c.2708G>A		Pathogenic	Uncertain significance
5279	c.2707T>C	rs1555589103	Benign	Uncertain significance
5280	c.2706A>C	rs398122665	Benign	Uncertain significance
5281	c.2702T>C	rs397507202	Benign	Uncertain significance
5282	c.2696T>G	rs1064794483	Benign	Uncertain significance
5283	c.2692A>G	rs80357420	Benign	Uncertain significance
5284	c.2690C>T	rs587776484	Benign	Uncertain significance
5285	c.2689C>T	rs770583134	Benign	Uncertain significance
5286	c.2686A>T	rs80357188	Pathogenic	Uncertain significance
5287	c.2684A>G	rs587781914	Benign	Uncertain significance
5288	c.2682A>T	rs1131692093	Benign	Uncertain significance
5289	c.2679G>T	rs587781771	Pathogenic	Uncertain significance
5290	c.2678A>G	rs1567794714	Benign	Uncertain significance
5291	c.2677A>C	rs80357170	Benign	Uncertain significance

5292	c.2666C>A	rs769712441	Pathogenic	Uncertain significance
5293	c.2663A>G	rs876658843	Benign	Uncertain significance
5294	c.2663A>T	rs876658843	Benign	Uncertain significance
5295	c.2663A>C	rs876658843	Benign	Uncertain significance
5296	c.2654T>A	rs1567794788	Benign	Uncertain significance
5297	c.2650A>G	rs80357120	Benign	Uncertain significance
5298	c.2648C>T	rs431825391	Pathogenic	Uncertain significance
5299	c.2647G>A		Pathogenic	Uncertain significance
5300	c.2645G>A	rs1555589241	Pathogenic	Uncertain significance
5301	c.2644T>C	rs184374817	Benign	Uncertain significance
5302	c.2644T>A	rs184374817	Benign	Uncertain significance
5303	c.2638G>C	rs587782370	Benign	Uncertain significance
5304	c.2635G>A	rs80357251	Pathogenic	Uncertain significance
5305	c.2633C>T	rs1555589255	Pathogenic	Uncertain significance
5306	c.2632G>A	rs80357230	Pathogenic	Uncertain significance
5307	c.2626G>A	rs1567794885	Pathogenic	Uncertain significance
5308	c.2623C>T	rs866257451	Benign	Uncertain significance
5309	c.2620A>G		Benign	Uncertain significance
5310	c.2620A>C	rs1064795862	Benign	Uncertain significance
5311	c.2618C>T	rs1567794912	Pathogenic	Uncertain significance
5312	c.2615T>C		Benign	Uncertain significance
5313	c.2612C>A	rs799917	Benign	Uncertain significance
5314	c.2609C>G	rs1060502324	Benign	Uncertain significance
5315	c.2609C>T	rs1060502324	Benign	Uncertain significance
5316	c.2608G>A	rs753256448	Pathogenic	Uncertain significance
5317	c.2599C>G		Benign	Uncertain significance
5318	c.2591C>T	rs80357003	Pathogenic	Uncertain significance
5319	c.2590T>G	rs80357285	Pathogenic	Uncertain significance
5320	c.2587G>A	rs1555589325	Benign	Uncertain significance
5321	c.2586G>C	rs1555589330	Benign	Uncertain significance
5322	c.2585A>G		Benign	Uncertain significance
5323	c.2583C>G	rs1555589334	Pathogenic	Uncertain significance
5324	c.2582T>G	rs80357098	Pathogenic	Uncertain significance
5325	c.2579C>T	rs1555589337	Benign	Uncertain significance
5326	c.2578A>G		Pathogenic	Uncertain significance
5327	c.2576A>G		Benign	Uncertain significance
5328	c.2575A>G	rs1555589354	Benign	Uncertain significance
5329	c.2569T>A		Pathogenic	Uncertain significance
5330	c.2567A>G	rs864622122	Pathogenic	Uncertain significance
5331	c.2564A>G	rs768001441	Pathogenic	Uncertain significance
5332	c.2564A>T	rs768001441	Pathogenic	Uncertain significance
5333	c.2564A>C	rs768001441	Benign	Uncertain significance
5334	c.2563C>A	rs80357131	Pathogenic	Uncertain significance
5335	c.2561C>T	rs80357315	Benign	Uncertain significance
5336	c.2560G>A		Pathogenic	Uncertain significance
5337	c.2555T>C	rs1555589415	Benign	Uncertain significance
5338	c.2554C>G	rs863224754	Benign	Uncertain significance
5339	c.2552A>G	rs1567795186	Benign	Uncertain significance
5340	c.2551G>A	rs398122662	Pathogenic	Uncertain significance
5341	c.2548A>C	rs1555589429	Benign	Uncertain significance
5342	c.2546A>G	rs1567795227	Benign	Uncertain significance
5343	c.2543A>G		Benign	Uncertain significance
5344	c.2541G>A	rs80357195	Pathogenic	Uncertain significance
5345	c.2539A>G	rs1555589446	Benign	Uncertain significance

5346	c.2538A>C	rs1567795266	Benign	Uncertain significance
5347	c.2536G>A	rs786203523	Pathogenic	Uncertain significance
5348	c.2535A>G		Pathogenic	Uncertain significance
5349	c.2534T>C	rs397508976	Benign	Uncertain significance
5350	c.2531G>A	rs56051266	Pathogenic	Uncertain significance
5351	c.2528C>G	rs1555589466	Benign	Uncertain significance
5352	c.2527A>G	rs80357435	Benign	Uncertain significance
5353	c.2524G>A	rs876658552	Pathogenic	Uncertain significance
5354	c.2522G>C	rs80357337	Benign	Uncertain significance
5355	c.2518A>C	rs377475866	Benign	Uncertain significance
5356	c.2518A>G	rs377475866	Pathogenic	Uncertain significance
5357	c.2516A>G		Benign	Uncertain significance
5358	c.2504A>C		Benign	Uncertain significance
5359	c.2503C>T	rs751656678	Benign	Uncertain significance
5360	c.2501G>T	rs757383244	Pathogenic	Uncertain significance
5361	c.2501G>A	rs757383244	Pathogenic	Uncertain significance
5362	c.2491T>C		Benign	Uncertain significance
5363	c.2491T>G	rs1060502350	Benign	Uncertain significance
5364	c.2489A>T		Pathogenic	Uncertain significance
5365	c.2483G>T	rs1555589578	Benign	Uncertain significance
5366	c.2482G>A	rs80357185	Benign	Uncertain significance
5367	c.2479G>C		Benign	Uncertain significance
5368	c.2477C>T		Benign	Uncertain significance
5369	c.2476A>C		Benign	Uncertain significance
5370	c.2474A>T	rs80357249	Benign	Uncertain significance
5371	c.2471A>G	rs1485586275	Benign	Uncertain significance
5372	c.2470A>C	rs1555589617	Benign	Uncertain significance
5373	c.2468G>C	rs876659731	Benign	Uncertain significance
5374	c.2468G>T	rs876659731	Pathogenic	Uncertain significance
5375	c.2466T>G	rs1064794701	Benign	Uncertain significance
5376	c.2465A>G	rs1555589630	Benign	Uncertain significance
5377	c.2456C>G	rs192655097	Benign	Uncertain significance
5378	c.2450G>T	rs1060502365	Benign	Uncertain significance
5379	c.2449G>A	rs1555589646	Pathogenic	Uncertain significance
5380	c.2447A>T	rs80357108	Benign	Uncertain significance
5381	c.2444T>C	rs730881484	Benign	Uncertain significance
5382	c.2437G>A	rs80357186	Benign	Uncertain significance
5383	c.2436G>A	rs1060502338	Pathogenic	Uncertain significance
5384	c.2435A>G		Benign	Uncertain significance
5385	c.2425G>A	rs786204151	Pathogenic	Uncertain significance
5386	c.2423T>C	rs398122660	Benign	Uncertain significance
5387	c.2420C>A	rs273899683	Benign	Uncertain significance
5388	c.2419G>A		Pathogenic	Uncertain significance
5389	c.2419G>T	rs80357240	Benign	Uncertain significance
5390	c.2417C>T	rs1555589705	Pathogenic	Uncertain significance
5391	c.2414G>A	rs1060502352	Pathogenic	Uncertain significance
5392	c.2410C>G	rs80356982	Pathogenic	Uncertain significance
5393	c.2405T>G	rs1555589737	Benign	Uncertain significance
5394	c.2404G>A	rs876660885	Pathogenic	Uncertain significance
5395	c.2404G>C	rs876660885	Pathogenic	Uncertain significance
5396	c.2404G>T	rs876660885	Benign	Uncertain significance
5397	c.2403T>G	rs80357381	Benign	Uncertain significance
5398	c.2402G>A	rs1567795821	Pathogenic	Uncertain significance
5399	c.2401T>C	rs1064793591	Benign	Uncertain significance

5400	c.2397T>A	rs80357203	Benign	Uncertain significance
5401	c.2396A>G	rs587782027	Benign	Uncertain significance
5402	c.2393C>G	rs876660005	Benign	Uncertain significance
5403	c.2392C>G	rs398122658	Benign	Uncertain significance
5404	c.2392C>A	rs398122658	Benign	Uncertain significance
5405	c.2392C>T	rs398122658	Benign	Uncertain significance
5406	c.2389G>A	rs62625306	Pathogenic	Uncertain significance
5407	c.2387C>G	rs80357364	Benign	Uncertain significance
5408	c.2387C>T	rs80357364	Benign	Uncertain significance
5409	c.2383A>G	rs1567795889	Benign	Uncertain significance
5410	c.2381C>T	rs7502059	Pathogenic	Uncertain significance
5411	c.2375G>A		Benign	Uncertain significance
5412	c.2372T>G		Benign	Uncertain significance
5413	c.2371C>G	rs1567795916	Benign	Uncertain significance
5414	c.2366G>A		Pathogenic	Uncertain significance
5415	c.2365A>G		Pathogenic	Uncertain significance
5416	c.2362G>A	rs80357060	Benign	Uncertain significance
5417	c.2357T>C	rs760864137	Benign	Uncertain significance
5418	c.2355A>C	rs1567795966	Benign	Uncertain significance
5419	c.2351C>G		Pathogenic	Uncertain significance
5420	c.2350T>G	rs80357399	Benign	Uncertain significance
5421	c.2346T>A	rs1555589837	Benign	Uncertain significance
5422	c.2345G>T	rs1567796012	Benign	Uncertain significance
5423	c.2344A>G	rs1433746078	Benign	Uncertain significance
5424	c.2342A>C	rs587776482	Benign	Uncertain significance
5425	c.2341G>C	rs757933953	Benign	Uncertain significance
5426	c.2339A>G	rs1410232200	Pathogenic	Uncertain significance
5427	c.2338C>G	rs80356945	Pathogenic	Uncertain significance
5428	c.2338C>A	rs80356945	Pathogenic	Uncertain significance
5429	c.2333G>A	rs730881483	Benign	Uncertain significance
5430	c.2329T>C		Benign	Uncertain significance
5431	c.2324C>T		Benign	Uncertain significance
5432	c.2321G>A	rs730881482	Pathogenic	Uncertain significance
5433	c.2320G>T	rs1555589917	Benign	Uncertain significance
5434	c.2314G>A	rs1567796129	Pathogenic	Uncertain significance
5435	c.2312T>C	rs730881481	Benign	Uncertain significance
5436	c.2303G>A		Pathogenic	Uncertain significance
5437	c.2302A>G	rs398122656	Pathogenic	Uncertain significance
5438	c.2300G>C	rs1567796202	Benign	Uncertain significance
5439	c.2299A>T	rs80357194	Pathogenic	Uncertain significance
5440	c.2299A>G	rs80357194	Pathogenic	Uncertain significance
5441	c.2298T>A		Benign	Uncertain significance
5442	c.2297G>A	rs1567796224	Pathogenic	Uncertain significance
5443	c.2296A>C	rs398122655	Benign	Uncertain significance
5444	c.2296A>G	rs398122655	Pathogenic	Uncertain significance
5445	c.2294A>G	rs80357085	Pathogenic	Uncertain significance
5446	c.2290G>A		Pathogenic	Uncertain significance
5447	c.2288C>A		Pathogenic	Uncertain significance
5448	c.2288C>G	rs876660112	Pathogenic	Uncertain significance
5449	c.2286A>G	rs273898682	Benign	Uncertain significance
5450	c.2282A>C	rs80356869	Benign	Uncertain significance
5451	c.2281G>C	rs397507198	Benign	Uncertain significance
5452	c.2281G>A	rs397507198	Pathogenic	Uncertain significance
5453	c.2279C>T		Benign	Uncertain significance

5454	c.2274G>T		Benign	Uncertain significance
5455	c.2269G>T	rs772617029	Benign	Uncertain significance
5456	c.2267G>A	rs975724885	Pathogenic	Uncertain significance
5457	c.2266A>G	rs1064795091	Benign	Uncertain significance
5458	c.2264A>C	rs922908090	Benign	Uncertain significance
5459	c.2263G>A	rs41286296	Pathogenic	Uncertain significance
5460	c.2259T>G	rs730881480	Benign	Uncertain significance
5461	c.2258G>A	rs878854939	Pathogenic	Uncertain significance
5462	c.2253G>C	rs1555590040	Benign	Uncertain significance
5463	c.2249T>C	rs1555590048	Benign	Uncertain significance
5464	c.2246A>T	rs730881479	Benign	Uncertain significance
5465	c.2245G>A	rs80357114	Pathogenic	Uncertain significance
5466	c.2245G>C	rs80357114	Benign	Uncertain significance
5467	c.2245G>T	rs80357114	Pathogenic	Uncertain significance
5468	c.2243A>G	rs1567796448	Benign	Uncertain significance
5469	c.2242A>G		Benign	Uncertain significance
5470	c.2239C>T	rs1555590087	Benign	Uncertain significance
5471	c.2238C>A	rs786202757	Benign	Uncertain significance
5472	c.2236G>T	rs876660267	Pathogenic	Uncertain significance
5473	c.2235A>C	rs876660266	Benign	Uncertain significance
5474	c.2232T>A		Benign	Uncertain significance
5475	c.2231C>A	rs786204220	Benign	Uncertain significance
5476	c.2228A>G	rs1567796489	Benign	Uncertain significance
5477	c.2227A>C	rs1212635015	Benign	Uncertain significance
5478	c.2225A>G	rs1060499618	Benign	Uncertain significance
5479	c.2222C>G	rs80357051	Benign	Uncertain significance
5480	c.2218G>A		Benign	Uncertain significance
5481	c.2218G>T	rs80357415	Benign	Uncertain significance
5482	c.2218G>C	rs80357415	Benign	Uncertain significance
5483	c.2217A>C	rs200521980	Benign	Uncertain significance
5484	c.2215A>C	rs56329598	Benign	Uncertain significance
5485	c.2215A>G	rs56329598	Pathogenic	Uncertain significance
5486	c.2209A>G	rs1567796585	Benign	Uncertain significance
5487	c.2203C>G	rs587781781	Benign	Uncertain significance
5488	c.2195A>C	rs876660463	Benign	Uncertain significance
5489	c.2185G>A	rs876659852	Pathogenic	Uncertain significance
5490	c.2183G>C	rs80357335	Benign	Uncertain significance
5491	c.2183G>A	rs80357335	Pathogenic	Uncertain significance
5492	c.2182A>G	rs1555590265	Benign	Uncertain significance
5493	c.2174G>C	rs1555590284	Benign	Uncertain significance
5494	c.2173A>G	rs1555590290	Pathogenic	Uncertain significance
5495	c.2171C>T	rs751104940	Benign	Uncertain significance
5496	c.2165T>C	rs1555590311	Benign	Uncertain significance
5497	c.2158G>A	rs80356875	Pathogenic	Uncertain significance
5498	c.2156A>C	rs876660920	Benign	Uncertain significance
5499	c.2153T>G	rs748550848	Benign	Uncertain significance
5500	c.2148T>A	rs730881478	Benign	Uncertain significance
5501	c.2147G>C	rs1555590369	Benign	Uncertain significance
5502	c.2143A>T	rs730881477	Benign	Uncertain significance
5503	c.2135G>T	rs1555590395	Benign	Uncertain significance
5504	c.2134T>C	rs786202015	Benign	Uncertain significance
5505	c.2131A>C	rs747046197	Benign	Uncertain significance
5506	c.2131A>G	rs747046197	Pathogenic	Uncertain significance
5507	c.2128A>G	rs876659959	Pathogenic	Uncertain significance

5508	c.2126T>G	rs1567796937	Benign	Uncertain significance
5509	c.2125T>G		Benign	Uncertain significance
5510	c.2123C>G	rs80357182	Benign	Uncertain significance
5511	c.2120G>A	rs80357192	Benign	Uncertain significance
5512	c.2119G>C	rs587781420	Benign	Uncertain significance
5513	c.2117C>T	rs759655692	Benign	Uncertain significance
5514	c.2116C>T		Benign	Uncertain significance
5515	c.2113G>A		Pathogenic	Uncertain significance
5516	c.2110A>C		Benign	Uncertain significance
5517	c.2108C>T	rs1302055573	Benign	Uncertain significance
5518	c.2102A>G	rs876658307	Benign	Uncertain significance
5519	c.2099T>A		Benign	Uncertain significance
5520	c.2099T>C	rs1567797015	Benign	Uncertain significance
5521	c.2096A>G		Pathogenic	Uncertain significance
5522	c.2095G>C	rs876658306	Benign	Uncertain significance
5523	c.2093C>T	rs1555590478	Benign	Uncertain significance
5524	c.2090T>A	rs730881476	Benign	Uncertain significance
5525	c.2090T>C	rs730881476	Benign	Uncertain significance
5526	c.2087C>T		Benign	Uncertain significance
5527	c.2081G>A	rs431825388	Pathogenic	Uncertain significance
5528	c.2078A>G	rs756748588	Benign	Uncertain significance
5529	c.2076T>A	rs587782595	Benign	Uncertain significance
5530	c.2075A>C		Benign	Uncertain significance
5531	c.2074C>T	rs545736576	Benign	Uncertain significance
5532	c.2072G>C	rs1555590574	Benign	Uncertain significance
5533	c.2071A>G		Benign	Uncertain significance
5534	c.2068A>G	rs587781448	Pathogenic	Uncertain significance
5535	c.2065A>G	rs876660188	Benign	Uncertain significance
5536	c.2062A>G		Benign	Uncertain significance
5537	c.2058A>T	rs1262148583	Benign	Uncertain significance
5538	c.2050C>A	rs397508934	Benign	Uncertain significance
5539	c.2050C>T	rs397508934	Benign	Uncertain significance
5540	c.2048A>G	rs1060502357	Benign	Uncertain significance
5541	c.2042G>A	rs1452826319	Pathogenic	Uncertain significance
5542	c.2039A>G	rs730881475	Benign	Uncertain significance
5543	c.2038A>G	rs1135401850	Benign	Uncertain significance
5544	c.2036A>C		Benign	Uncertain significance
5545	c.2033C>T	rs1555590634	Benign	Uncertain significance
5546	c.2029G>A		Pathogenic	Uncertain significance
5547	c.2027C>T		Benign	Uncertain significance
5548	c.2023G>A		Pathogenic	Uncertain significance
5549	c.2021C>G	rs876660543	Benign	Uncertain significance
5550	c.2017G>C		Benign	Uncertain significance
5551	c.2014A>G	rs397508929	Pathogenic	Uncertain significance
5552	c.2008G>A	rs80357029	Pathogenic	Uncertain significance
5553	c.1997T>C	rs1567797346	Benign	Uncertain significance
5554	c.1996C>G	rs1555590709	Benign	Uncertain significance
5555	c.1995C>G	rs80357238	Benign	Uncertain significance
5556	c.1985A>G	rs80357494	Benign	Uncertain significance
5557	c.1984C>T	rs397508927	Benign	Uncertain significance
5558	c.1981A>G	rs1555590724	Pathogenic	Uncertain significance
5559	c.1979T>C	rs1567797385	Benign	Uncertain significance
5560	c.1978G>T		Benign	Uncertain significance
5561	c.1978G>A	rs876660889	Benign	Uncertain significance

5562	c.1975C>G	rs587776481	Benign	Uncertain significance
5563	c.1969C>A	rs397508926	Benign	Uncertain significance
5564	c.1967A>G	rs397508925	Benign	Uncertain significance
5565	c.1966A>T	rs786203455	Pathogenic	Uncertain significance
5566	c.1964A>T	rs80357193	Benign	Uncertain significance
5567	c.1963T>G	rs80357166	Benign	Uncertain significance
5568	c.1954A>G	rs1567797589	Benign	Uncertain significance
5569	c.1951A>G		Benign	Uncertain significance
5570	c.1951A>C	rs1555590804	Benign	Uncertain significance
5571	c.1949T>G	rs1555590816	Benign	Uncertain significance
5572	c.1949T>C	rs1555590816	Benign	Uncertain significance
5573	c.1945G>C	rs80356907	Benign	Uncertain significance
5574	c.1934C>G	rs80357129	Benign	Uncertain significance
5575	c.1934C>A	rs80357129	Pathogenic	Uncertain significance
5576	c.1933T>C		Benign	Uncertain significance
5577	c.1931G>C	rs876658606	Benign	Uncertain significance
5578	c.1931G>T	rs876658606	Benign	Uncertain significance
5579	c.1930T>A	rs753521391	Pathogenic	Uncertain significance
5580	c.1929T>G	rs1060502361	Benign	Uncertain significance
5581	c.1928G>T	rs876660335	Benign	Uncertain significance
5582	c.1925A>G	rs786204049	Benign	Uncertain significance
5583	c.1924G>A	rs80357344	Pathogenic	Uncertain significance
5584	c.1924G>T	rs80357344	Pathogenic	Uncertain significance
5585	c.1922T>C	rs730881474	Benign	Uncertain significance
5586	c.1920A>C		Benign	Uncertain significance
5587	c.1920A>T	rs587782843	Pathogenic	Uncertain significance
5588	c.1919A>C	rs786203965	Benign	Uncertain significance
5589	c.1917G>T		Benign	Uncertain significance
5590	c.1913A>C	rs786201944	Benign	Uncertain significance
5591	c.1910C>T	rs1555590925	Benign	Uncertain significance
5592	c.1907G>C	rs398122649	Benign	Uncertain significance
5593	c.1906T>G		Benign	Uncertain significance
5594	c.1905T>G		Benign	Uncertain significance
5595	c.1904A>G		Benign	Uncertain significance
5596	c.1903A>G	rs1567797783	Pathogenic	Uncertain significance
5597	c.1901C>T	rs80357121	Benign	Uncertain significance
5598	c.1901C>G	rs80357121	Benign	Uncertain significance
5599	c.1900C>T	rs80357056	Benign	Uncertain significance
5600	c.1898C>T	rs398122647	Benign	Uncertain significance
5601	c.1897C>A	rs80356902	Pathogenic	Uncertain significance
5602	c.1895G>A	rs80356983	Pathogenic	Uncertain significance
5603	c.1892T>C	rs1386991114	Benign	Uncertain significance
5604	c.1891C>A	rs876659175	Pathogenic	Uncertain significance
5605	c.1888A>T		Pathogenic	Uncertain significance
5606	c.1886G>T	rs876660144	Pathogenic	Uncertain significance
5607	c.1885A>G		Pathogenic	Uncertain significance
5608	c.1884T>G	rs80357495	Benign	Uncertain significance
5609	c.1882A>G	rs1555591000	Pathogenic	Uncertain significance
5610	c.1881C>G	rs80356838	Pathogenic	Uncertain significance
5611	c.1880T>A	rs770002293	Benign	Uncertain significance
5612	c.1880T>G	rs770002293	Benign	Uncertain significance
5613	c.1870G>A	rs80356950	Pathogenic	Uncertain significance
5614	c.1868T>C	rs397508915	Benign	Uncertain significance
5615	c.1865C>G		Benign	Uncertain significance

5616	c.1864G>A	rs1567797954	Pathogenic	Uncertain significance
5617	c.1861C>T		Benign	Uncertain significance
5618	c.1859T>A	rs1555591085	Benign	Uncertain significance
5619	c.1856A>G	rs771890863	Benign	Uncertain significance
5620	c.1854G>T		Benign	Uncertain significance
5621	c.1853G>C	rs876659527	Benign	Uncertain significance
5622	c.1853G>A	rs876659527	Pathogenic	Uncertain significance
5623	c.1844C>T	rs398122645	Benign	Uncertain significance
5624	c.1842G>T	rs760109939	Benign	Uncertain significance
5625	c.1841A>G		Benign	Uncertain significance
5626	c.1838G>T		Benign	Uncertain significance
5627	c.1838G>A	rs786203937	Pathogenic	Uncertain significance
5628	c.1829G>C	rs876660322	Benign	Uncertain significance
5629	c.1829G>A	rs876660322	Pathogenic	Uncertain significance
5630	c.1828A>G	rs398122644	Pathogenic	Uncertain significance
5631	c.1826A>G	rs80357236	Benign	Uncertain significance
5632	c.1819A>G	rs80357220	Pathogenic	Uncertain significance
5633	c.1814C>A	rs1555591195	Benign	Uncertain significance
5634	c.1813G>C	rs587781613	Benign	Uncertain significance
5635	c.1813G>T	rs587781613	Benign	Uncertain significance
5636	c.1807T>G	rs1555591208	Pathogenic	Uncertain significance
5637	c.1800C>G	rs80357452	Pathogenic	Uncertain significance
5638	c.1799T>C	rs398122643	Benign	Uncertain significance
5639	c.1799T>G	rs398122643	Benign	Uncertain significance
5640	c.1796A>G		Benign	Uncertain significance
5641	c.1792T>G	rs1060504554	Benign	Uncertain significance
5642	c.1787T>C		Benign	Uncertain significance
5643	c.1786C>T	rs80357371	Benign	Uncertain significance
5644	c.1786C>G	rs80357371	Benign	Uncertain significance
5645	c.1784A>G	rs876660455	Benign	Uncertain significance
5646	c.1774A>C		Benign	Uncertain significance
5647	c.1773A>G	rs1555591259	Pathogenic	Uncertain significance
5648	c.1771A>G	rs1064795358	Pathogenic	Uncertain significance
5649	c.1769G>A	rs1060502349	Pathogenic	Uncertain significance
5650	c.1768A>G	rs80357454	Pathogenic	Uncertain significance
5651	c.1767C>A	rs587783039	Benign	Uncertain significance
5652	c.1766G>T		Benign	Uncertain significance
5653	c.1766G>A	rs1567798335	Pathogenic	Uncertain significance
5654	c.1764C>G		Benign	Uncertain significance
5655	c.1762A>G	rs1169162396	Benign	Uncertain significance
5656	c.1761A>G		Pathogenic	Uncertain significance
5657	c.1760T>C		Benign	Uncertain significance
5658	c.1759A>G		Pathogenic	Uncertain significance
5659	c.1757C>G	rs1064795270	Benign	Uncertain significance
5660	c.1756C>T	rs80357153	Benign	Uncertain significance
5661	c.1745C>A	rs786202386	Benign	Uncertain significance
5662	c.1742A>G		Benign	Uncertain significance
5663	c.1733C>T	rs80356939	Benign	Uncertain significance
5664	c.1733C>A	rs80356939	Pathogenic	Uncertain significance
5665	c.1729G>C		Benign	Uncertain significance
5666	c.1723G>A	rs397508902	Benign	Uncertain significance
5667	c.1721T>C	rs1060502341	Benign	Uncertain significance
5668	c.1718C>T	rs876660434	Benign	Uncertain significance
5669	c.1717T>A		Benign	Uncertain significance

5670	c.1717T>C	rs876660448	Benign	Uncertain significance
5671	c.1714G>A	rs730881473	Pathogenic	Uncertain significance
5672	c.1714G>C	rs730881473	Benign	Uncertain significance
5673	c.1712T>G		Benign	Uncertain significance
5674	c.1712T>A	rs80357159	Benign	Uncertain significance
5675	c.1711A>G	rs1310719199	Benign	Uncertain significance
5676	c.1709C>A	rs879254020	Benign	Uncertain significance
5677	c.1706A>G		Benign	Uncertain significance
5678	c.1706A>T	rs1060502329	Pathogenic	Uncertain significance
5679	c.1705A>G	rs587781315	Pathogenic	Uncertain significance
5680	c.1703C>G	rs80356910	Benign	Uncertain significance
5681	c.1702C>T	rs755122577	Benign	Uncertain significance
5682	c.1701T>G	rs1555591359	Benign	Uncertain significance
5683	c.1696A>C		Benign	Uncertain significance
5684	c.1696A>G		Benign	Uncertain significance
5685	c.1690A>T	rs397507191	Pathogenic	Uncertain significance
5686	c.1686T>G	rs1268133978	Pathogenic	Uncertain significance
5687	c.1685T>C	rs1555591375	Benign	Uncertain significance
5688	c.1684A>G	rs1567798727	Pathogenic	Uncertain significance
5689	c.1683T>C	rs1567798735	Benign	Uncertain significance
5690	c.1679A>T	rs1567798739	Benign	Uncertain significance
5691	c.1676G>A	rs80356980	Pathogenic	Uncertain significance
5692	c.1676G>T	rs80356980	Pathogenic	Uncertain significance
5693	c.1675G>A	rs1555591384	Pathogenic	Uncertain significance
5694	c.1672A>C	rs397507190	Benign	Uncertain significance
5695	c.1670C>T	rs1567798771	Benign	Uncertain significance
5696	c.1666A>G	rs587783041	Pathogenic	Uncertain significance
5697	c.1666A>C	rs587783041	Benign	Uncertain significance
5698	c.1662G>C	rs876659028	Benign	Uncertain significance
5699	c.1661A>G		Pathogenic	Uncertain significance
5700	c.1658A>C	rs748431827	Benign	Uncertain significance
5701	c.1655G>A	rs397508893	Pathogenic	Uncertain significance
5702	c.1654G>A	rs758598971	Pathogenic	Uncertain significance
5703	c.1651A>G	rs730881472	Pathogenic	Uncertain significance
5704	c.1649A>G	rs1064795217	Benign	Uncertain significance
5705	c.1642A>G	rs80356981	Pathogenic	Uncertain significance
5706	c.1639A>T	rs1060502351	Pathogenic	Uncertain significance
5707	c.1639A>C	rs1060502351	Benign	Uncertain significance
5708	c.1638G>A	rs1060502340	Pathogenic	Uncertain significance
5709	c.1636A>G	rs587782390	Benign	Uncertain significance
5710	c.1631A>T	rs397508892	Pathogenic	Uncertain significance
5711	c.1631A>C	rs397508892	Benign	Uncertain significance
5712	c.1624A>G	rs1555591461	Benign	Uncertain significance
5713	c.1620G>C	rs1555591465	Benign	Uncertain significance
5714	c.1618G>A	rs730881471	Pathogenic	Uncertain significance
5715	c.1616C>A	rs80357374	Benign	Uncertain significance
5716	c.1607C>T		Benign	Uncertain significance
5717	c.1607C>A	rs398122638	Benign	Uncertain significance
5718	c.1607C>G	rs398122638	Benign	Uncertain significance
5719	c.1604G>C	rs1064793602	Benign	Uncertain significance
5720	c.1603G>A	rs1555591488	Benign	Uncertain significance
5721	c.1589A>T		Pathogenic	Uncertain significance
5722	c.1589A>G	rs1555591512	Benign	Uncertain significance
5723	c.1580A>G	rs774959350	Benign	Uncertain significance

5724	c.1574T>C	rs879253902	Benign	Uncertain significance
5725	c.1571C>T	rs80357333	Pathogenic	Uncertain significance
5726	c.1568T>G	rs397508885	Benign	Uncertain significance
5727	c.1564G>A	rs80357453	Pathogenic	Uncertain significance
5728	c.1561G>C	rs80357122	Benign	Uncertain significance
5729	c.1558A>C	rs1555591572	Benign	Uncertain significance
5730	c.1555A>C	rs397508882	Benign	Uncertain significance
5731	c.1554C>A	rs1064794487	Pathogenic	Uncertain significance
5732	c.1553T>C	rs1567799193	Benign	Uncertain significance
5733	c.1552A>G		Pathogenic	Uncertain significance
5734	c.1543G>A	rs886037990	Pathogenic	Uncertain significance
5735	c.1541C>G	rs56100707	Benign	Uncertain significance
5736	c.1541C>T	rs56100707	Benign	Uncertain significance
5737	c.1540C>A	rs1555591584	Benign	Uncertain significance
5738	c.1538A>G	rs1356078500	Pathogenic	Uncertain significance
5739	c.1534C>G	rs41286294	Benign	Uncertain significance
5740	c.1532G>T	rs397507188	Benign	Uncertain significance
5741	c.1531G>A	rs1567799248	Pathogenic	Uncertain significance
5742	c.1523C>T	rs398122637	Benign	Uncertain significance
5743	c.1520G>T	rs80357224	Pathogenic	Uncertain significance
5744	c.1514A>T	rs879254266	Pathogenic	Uncertain significance
5745	c.1514A>G	rs879254266	Benign	Uncertain significance
5746	c.1510C>T	rs80357445	Benign	Uncertain significance
5747	c.1505T>C		Benign	Uncertain significance
5748	c.1502A>C	rs113656989	Benign	Uncertain significance
5749	c.1501A>C		Benign	Uncertain significance
5750	c.1501A>G	rs1064793883	Pathogenic	Uncertain significance
5751	c.1499A>G	rs1555591677	Benign	Uncertain significance
5752	c.1498A>G	rs1555591681	Benign	Uncertain significance
5753	c.1496C>T	rs876658285	Benign	Uncertain significance
5754	c.1495A>C		Benign	Uncertain significance
5755	c.1490C>T	rs1555591693	Benign	Uncertain significance
5756	c.1489C>A	rs1555591695	Benign	Uncertain significance
5757	c.1486C>A	rs28897676	Benign	Uncertain significance
5758	c.1484A>C	rs1567799498	Benign	Uncertain significance
5759	c.1478T>C	rs1555591707	Benign	Uncertain significance
5760	c.1477A>C		Benign	Uncertain significance
5761	c.1474A>G		Benign	Uncertain significance
5762	c.1472A>C	rs80357376	Benign	Uncertain significance
5763	c.1472A>G	rs80357376	Pathogenic	Uncertain significance
5764	c.1471C>G	rs62625303	Benign	Uncertain significance
5765	c.1469C>T	rs876658291	Benign	Uncertain significance
5766	c.1465G>A	rs80357167	Pathogenic	Uncertain significance
5767	c.1460T>G	rs748812609	Benign	Uncertain significance
5768	c.1460T>C	rs748812609	Benign	Uncertain significance
5769	c.1458T>G	rs80357400	Benign	Uncertain significance
5770	c.1450G>A	rs80357304	Pathogenic	Uncertain significance
5771	c.1448T>C	rs80357489	Benign	Uncertain significance
5772	c.1441C>A		Pathogenic	Uncertain significance
5773	c.1436A>G		Benign	Uncertain significance
5774	c.1435G>C		Benign	Uncertain significance
5775	c.1435G>A	rs1064794100	Pathogenic	Uncertain significance
5776	c.1424G>A		Pathogenic	Uncertain significance
5777	c.1423A>G		Benign	Uncertain significance

5778	c.1423A>T	rs1064794047	Pathogenic	Uncertain significance
5779	c.1418A>C		Benign	Uncertain significance
5780	c.1412T>C		Benign	Uncertain significance
5781	c.1409G>A	rs1567799713	Pathogenic	Uncertain significance
5782	c.1408A>G		Benign	Uncertain significance
5783	c.1406C>G	rs80357073	Benign	Uncertain significance
5784	c.1396C>G	rs80356964	Pathogenic	Uncertain significance
5785	c.1394A>G	rs876659885	Pathogenic	Uncertain significance
5786	c.1391C>T	rs62625301	Benign	Uncertain significance
5787	c.1387A>G	rs1135401844	Benign	Uncertain significance
5788	c.1384G>A	rs80357221	Pathogenic	Uncertain significance
5789	c.1381T>C	rs62625300	Benign	Uncertain significance
5790	c.1379T>C		Benign	Uncertain significance
5791	c.1379T>G	rs398122634	Benign	Uncertain significance
5792	c.1374C>A	rs879253999	Benign	Uncertain significance
5793	c.1367T>C	rs80357360	Benign	Uncertain significance
5794	c.1363A>G		Pathogenic	Uncertain significance
5795	c.1361G>A	rs80357181	Pathogenic	Uncertain significance
5796	c.1355T>C	rs878854932	Benign	Uncertain significance
5797	c.1350A>T		Benign	Uncertain significance
5798	c.1345T>C		Benign	Uncertain significance
5799	c.1343A>C		Benign	Uncertain significance
5800	c.1342C>T	rs786203578	Benign	Uncertain significance
5801	c.1339G>A	rs587782784	Benign	Uncertain significance
5802	c.1336A>G	rs587781715	Benign	Uncertain significance
5803	c.1334A>G		Benign	Uncertain significance
5804	c.1333G>C	rs80356915	Benign	Uncertain significance
5805	c.1332T>G	rs1555591925	Benign	Uncertain significance
5806	c.1331G>A		Pathogenic	Uncertain significance
5807	c.1324T>A	rs876660734	Benign	Uncertain significance
5808	c.1324T>C	rs876660734	Benign	Uncertain significance
5809	c.1323A>G	rs1555591935	Pathogenic	Uncertain significance
5810	c.1322T>C		Benign	Uncertain significance
5811	c.1321A>G	rs1567800029	Benign	Uncertain significance
5812	c.1319T>C	rs273897656	Benign	Uncertain significance
5813	c.1316C>G		Benign	Uncertain significance
5814	c.1315G>A		Benign	Uncertain significance
5815	c.1315G>T	rs1064794098	Benign	Uncertain significance
5816	c.1313A>C	rs730881470	Benign	Uncertain significance
5817	c.1310A>G	rs80357255	Pathogenic	Uncertain significance
5818	c.1310A>C	rs80357255	Benign	Uncertain significance
5819	c.1309C>T	rs759878392	Benign	Uncertain significance
5820	c.1300A>C		Benign	Uncertain significance
5821	c.1300A>G	rs786203753	Pathogenic	Uncertain significance
5822	c.1295T>C	rs369394098	Benign	Uncertain significance
5823	c.1291T>A	rs960381964	Benign	Uncertain significance
5824	c.1289A>G		Benign	Uncertain significance
5825	c.1286T>G	rs775869160	Benign	Uncertain significance
5826	c.1286T>C	rs775869160	Benign	Uncertain significance
5827	c.1270G>A	rs763051683	Pathogenic	Uncertain significance
5828	c.1270G>C	rs763051683	Benign	Uncertain significance
5829	c.1265A>T	rs1567800230	Benign	Uncertain significance
5830	c.1264T>C	rs764186025	Benign	Uncertain significance
5831	c.1261G>A	rs80357046	Pathogenic	Uncertain significance

5832	c.1259A>G	rs730881442	Benign	Uncertain significance
5833	c.1258G>C		Benign	Uncertain significance
5834	c.1258G>A	rs80357488	Pathogenic	Uncertain significance
5835	c.1256T>G	rs398122628	Benign	Uncertain significance
5836	c.1255G>A	rs876658873	Pathogenic	Uncertain significance
5837	c.1255G>C	rs876658873	Pathogenic	Uncertain significance
5838	c.1251T>G	rs80357197	Benign	Uncertain significance
5839	c.1244T>C	rs1064796031	Benign	Uncertain significance
5840	c.1243G>T	rs587782770	Benign	Uncertain significance
5841	c.1241A>C	rs1555592086	Benign	Uncertain significance
5842	c.1234G>A	rs587776478	Pathogenic	Uncertain significance
5843	c.1232A>T	rs730881469	Benign	Uncertain significance
5844	c.1231G>A	rs80357301	Pathogenic	Uncertain significance
5845	c.1228G>A	rs779974365	Pathogenic	Uncertain significance
5846	c.1226T>C	rs786202539	Benign	Uncertain significance
5847	c.1208C>T	rs80356934	Benign	Uncertain significance
5848	c.1207T>G		Benign	Uncertain significance
5849	c.1207T>C	rs1555592200	Benign	Uncertain significance
5850	c.1206G>C		Benign	Uncertain significance
5851	c.1205A>T	rs1555592216	Pathogenic	Uncertain significance
5852	c.1204G>A		Pathogenic	Uncertain significance
5853	c.1202G>C	rs397507184	Pathogenic	Uncertain significance
5854	c.1201G>A	rs1555592242	Pathogenic	Uncertain significance
5855	c.1199A>G	rs1555592245	Benign	Uncertain significance
5856	c.1196A>G	rs587780794	Benign	Uncertain significance
5857	c.1193C>T		Benign	Uncertain significance
5858	c.1190A>T		Benign	Uncertain significance
5859	c.1187A>T	rs1555592280	Benign	Uncertain significance
5860	c.1187A>G	rs1555592280	Benign	Uncertain significance
5861	c.1186G>A	rs786203145	Pathogenic	Uncertain significance
5862	c.1181G>A		Benign	Uncertain significance
5863	c.1181G>T	rs1555592295	Pathogenic	Uncertain significance
5864	c.1175T>C	rs777305766	Benign	Uncertain significance
5865	c.1163G>C	rs786203567	Benign	Uncertain significance
5866	c.1160C>G	rs1567800665	Benign	Uncertain significance
5867	c.1155G>T	rs876660558	Benign	Uncertain significance
5868	c.1147A>C		Benign	Uncertain significance
5869	c.1144G>T		Benign	Uncertain significance
5870	c.1142A>G	rs1555592379	Benign	Uncertain significance
5871	c.1139A>G	rs876659193	Pathogenic	Uncertain significance
5872	c.1139A>C	rs876659193	Benign	Uncertain significance
5873	c.1136T>C	rs1567800757	Benign	Uncertain significance
5874	c.1135A>G	rs864622723	Pathogenic	Uncertain significance
5875	c.1134C>A	rs863224752	Benign	Uncertain significance
5876	c.1132A>G	rs1555592404	Pathogenic	Uncertain significance
5877	c.1131C>G	rs786203434	Benign	Uncertain significance
5878	c.1131C>A	rs786203434	Benign	Uncertain significance
5879	c.1130G>A	rs80357398	Pathogenic	Uncertain significance
5880	c.1129A>G	rs1555592415	Pathogenic	Uncertain significance
5881	c.1127A>G	rs80356976	Benign	Uncertain significance
5882	c.1121C>T	rs80357235	Benign	Uncertain significance
5883	c.1114T>C	rs1306111238	Benign	Uncertain significance
5884	c.1114T>A	rs1306111238	Benign	Uncertain significance
5885	c.1108G>A		Pathogenic	Uncertain significance

5886	c.1108G>C	rs1567800850	Benign	Uncertain significance
5887	c.1106A>T	rs80357416	Benign	Uncertain significance
5888	c.1106A>G	rs80357416	Benign	Uncertain significance
5889	c.1099A>G	rs878854929	Pathogenic	Uncertain significance
5890	c.1097A>T	rs587781769	Benign	Uncertain significance
5891	c.1096G>C	rs1289961661	Benign	Uncertain significance
5892	c.1090C>A	rs876660309	Benign	Uncertain significance
5893	c.1082C>T	rs397508833	Benign	Uncertain significance
5894	c.1078T>C	rs587782790	Benign	Uncertain significance
5895	c.1075C>T	rs767666190	Benign	Uncertain significance
5896	c.1069A>G		Benign	Uncertain significance
5897	c.1064A>G	rs80357246	Benign	Uncertain significance
5898	c.1061A>G	rs1555592577	Benign	Uncertain significance
5899	c.1061A>T	rs1555592577	Benign	Uncertain significance
5900	c.1059G>C	rs80356935	Benign	Uncertain significance
5901	c.1054G>A	rs80357472	Pathogenic	Uncertain significance
5902	c.1052A>T		Pathogenic	Uncertain significance
5903	c.1043G>A	rs752198747	Pathogenic	Uncertain significance
5904	c.1042T>C	rs786201928	Benign	Uncertain significance
5905	c.1040T>A	rs757987511	Benign	Uncertain significance
5906	c.1037C>A	rs1555592620	Pathogenic	Uncertain significance
5907	c.1036C>G	rs80357015	Pathogenic	Uncertain significance
5908	c.1033G>T	rs80356961	Pathogenic	Uncertain significance
5909	c.1031C>A	rs876658636	Benign	Uncertain significance
5910	c.1031C>T	rs876658636	Benign	Uncertain significance
5911	c.1030G>A	rs79727659	Pathogenic	Uncertain significance
5912	c.1025T>A		Benign	Uncertain significance
5913	c.1025T>C	rs1555592634	Benign	Uncertain significance
5914	c.1022A>C	rs1567801162	Benign	Uncertain significance
5915	c.1021G>T	rs756987689	Pathogenic	Uncertain significance
5916	c.1021G>A	rs756987689	Pathogenic	Uncertain significance
5917	c.1017G>T	rs863224416	Benign	Uncertain significance
5918	c.1016A>C	rs587781737	Benign	Uncertain significance
5919	c.1012A>G	rs397508826	Benign	Uncertain significance
5920	c.1007C>G		Benign	Uncertain significance
5921	c.1005C>A	rs876660367	Benign	Uncertain significance
5922	c.1000C>A	rs1555592700	Benign	Uncertain significance
5923	c.998C>G	rs431825420	Benign	Uncertain significance
5924	c.998C>T	rs431825420	Benign	Uncertain significance
5925	c.994C>G		Benign	Uncertain significance
5926	c.993G>C	rs80357140	Benign	Uncertain significance
5927	c.988G>A	rs397507259	Pathogenic	Uncertain significance
5928	c.985A>T	rs786203732	Pathogenic	Uncertain significance
5929	c.983G>A		Pathogenic	Uncertain significance
5930	c.982T>G	rs748156170	Benign	Uncertain significance
5931	c.982T>C	rs748156170	Benign	Uncertain significance
5932	c.976G>C		Benign	Uncertain significance
5933	c.971G>A	rs1462728426	Pathogenic	Uncertain significance
5934	c.967G>A		Pathogenic	Uncertain significance
5935	c.954T>A	rs1135401826	Benign	Uncertain significance
5936	c.953A>G	rs776278453	Benign	Uncertain significance
5937	c.948C>G	rs1555592782	Benign	Uncertain significance
5938	c.947G>A	rs1465372282	Pathogenic	Uncertain significance
5939	c.945G>C		Benign	Uncertain significance

5940	c.944G>A	rs1555592792	Benign	Uncertain significance
5941	c.943A>G	rs80357050	Benign	Uncertain significance
5942	c.941C>T	rs863224766	Pathogenic	Uncertain significance
5943	c.932C>T	rs1301795658	Benign	Uncertain significance
5944	c.929A>C		Benign	Uncertain significance
5945	c.926A>C	rs80356877	Benign	Uncertain significance
5946	c.925A>G	rs879255498	Benign	Uncertain significance
5947	c.924C>T	rs1555592819	Pathogenic	Uncertain significance
5948	c.923G>A	rs561998108	Pathogenic	Uncertain significance
5949	c.923G>C	rs561998108	Benign	Uncertain significance
5950	c.922A>G	rs55767801	Benign	Uncertain significance
5951	c.919A>G	rs1216516227	Pathogenic	Uncertain significance
5952	c.914G>T		Benign	Uncertain significance
5953	c.914G>A	rs751124745	Pathogenic	Uncertain significance
5954	c.910T>C	rs1567801570	Benign	Uncertain significance
5955	c.903G>T		Benign	Uncertain significance
5956	c.902A>G	rs878854965	Benign	Uncertain significance
5957	c.900A>C	rs80356861	Benign	Uncertain significance
5958	c.898G>A	rs886040330	Pathogenic	Uncertain significance
5959	c.896T>C		Benign	Uncertain significance
5960	c.894T>C		Benign	Uncertain significance
5961	c.890T>A	rs80356924	Benign	Uncertain significance
5962	c.889A>G	rs80357196	Benign	Uncertain significance
5963	c.885C>G		Benign	Uncertain significance
5964	c.884A>G	rs772684048	Benign	Uncertain significance
5965	c.881A>G	rs1555592934	Benign	Uncertain significance
5966	c.880A>G	rs1567801705	Pathogenic	Uncertain significance
5967	c.878C>G	rs747172803	Benign	Uncertain significance
5968	c.878C>T	rs747172803	Benign	Uncertain significance
5969	c.877A>C	rs1555592945	Benign	Uncertain significance
5970	c.869T>C	rs730881468	Benign	Uncertain significance
5971	c.866G>C		Benign	Uncertain significance
5972	c.866G>A	rs776999497	Benign	Uncertain significance
5973	c.862A>G		Pathogenic	Uncertain significance
5974	c.861C>G	rs1064794692	Benign	Uncertain significance
5975	c.852G>C		Benign	Uncertain significance
5976	c.851A>G	rs80357039	Pathogenic	Uncertain significance
5977	c.845C>T	rs786203027	Benign	Uncertain significance
5978	c.842G>C		Benign	Uncertain significance
5979	c.841A>G	rs1300771163	Pathogenic	Uncertain significance
5980	c.839C>A		Benign	Uncertain significance
5981	c.836A>T	rs80357482	Benign	Uncertain significance
5982	c.836A>G	rs80357482	Pathogenic	Uncertain significance
5983	c.835C>T		Benign	Uncertain significance
5984	c.827C>A	rs80357436	Benign	Uncertain significance
5985	c.821G>A		Pathogenic	Uncertain significance
5986	c.820T>C		Benign	Uncertain significance
5987	c.817C>T		Benign	Uncertain significance
5988	c.812T>C	rs753099787	Benign	Uncertain significance
5989	c.812T>G	rs753099787	Benign	Uncertain significance
5990	c.811G>T	rs80357244	Benign	Uncertain significance
5991	c.811G>C	rs80357244	Benign	Uncertain significance
5992	c.809A>G		Benign	Uncertain significance
5993	c.808C>T		Benign	Uncertain significance

5994	c.804C>G	rs771076131	Benign	Uncertain significance
5995	c.803A>G		Benign	Uncertain significance
5996	c.802A>G	rs1555593066	Benign	Uncertain significance
5997	c.799T>C	rs587781496	Benign	Uncertain significance
5998	c.792T>G	rs80357214	Benign	Uncertain significance
5999	c.788G>A	rs397509319	Pathogenic	Uncertain significance
6000	c.787G>A	rs397509318	Pathogenic	Uncertain significance
6001	c.785A>G	rs1555593115	Pathogenic	Uncertain significance
6002	c.779A>G		Benign	Uncertain significance
6003	c.778A>C	rs786202263	Benign	Uncertain significance
6004	c.773C>G	rs80357225	Benign	Uncertain significance
6005	c.769C>T		Benign	Uncertain significance
6006	c.768G>C		Benign	Uncertain significance
6007	c.767G>A	rs11658785	Pathogenic	Uncertain significance
6008	c.766A>T	rs587781833	Pathogenic	Uncertain significance
6009	c.765G>C		Benign	Uncertain significance
6010	c.758C>T	rs1555593177	Pathogenic	Uncertain significance
6011	c.757G>A	rs80357293	Pathogenic	Uncertain significance
6012	c.748G>A	rs1567802220	Pathogenic	Uncertain significance
6013	c.745A>T	rs397507256	Benign	Uncertain significance
6014	c.743C>A	rs80357062	Benign	Uncertain significance
6015	c.742A>G	rs879255288	Benign	Uncertain significance
6016	c.735T>A	rs1567802284	Benign	Uncertain significance
6017	c.734A>T	rs80356865	Benign	Uncertain significance
6018	c.733G>C		Benign	Uncertain significance
6019	c.733G>T	rs147519994	Pathogenic	Uncertain significance
6020	c.729T>G	rs730881467	Benign	Uncertain significance
6021	c.727A>G	rs587782123	Pathogenic	Uncertain significance
6022	c.725G>A		Pathogenic	Uncertain significance
6023	c.724A>G	rs1555593258	Pathogenic	Uncertain significance
6024	c.722C>T	rs80357351	Benign	Uncertain significance
6025	c.721C>A	rs1555593260	Benign	Uncertain significance
6026	c.716A>T	rs80357396	Benign	Uncertain significance
6027	c.715C>T		Benign	Uncertain significance
6028	c.713A>G	rs587782094	Benign	Uncertain significance
6029	c.710A>C		Benign	Uncertain significance
6030	c.707C>G	rs80356990	Benign	Uncertain significance
6031	c.704A>G	rs1555593283	Benign	Uncertain significance
6032	c.701C>A	rs1555593290	Benign	Uncertain significance
6033	c.701C>T	rs1555593290	Benign	Uncertain significance
6034	c.700A>G		Benign	Uncertain significance
6035	c.695A>T	rs398122708	Benign	Uncertain significance
6036	c.694G>T	rs55975699	Pathogenic	Uncertain significance
6037	c.686C>T		Benign	Uncertain significance
6038	c.683T>A	rs191872612	Pathogenic	Uncertain significance
6039	c.677G>C		Benign	Uncertain significance
6040	c.677G>A	rs1376262238	Pathogenic	Uncertain significance
6041	c.671C>T	rs1060502335	Benign	Uncertain significance
6042	c.671-98G>C	rs273902785	Benign	Uncertain significance
6043	c.671-141T>A	rs273902782	Benign	Uncertain significance
6044	c.670G>A	rs431825419	Pathogenic	Uncertain significance
6045	c.665A>C		Benign	Uncertain significance
6046	c.665A>G	rs398122705	Benign	Uncertain significance
6047	c.662C>T		Pathogenic	Uncertain significance

6048	c.659C>G	rs431825418	Benign	Uncertain significance
6049	c.655G>C	rs273902779	Benign	Uncertain significance
6050	c.650G>C	rs774284145	Benign	Uncertain significance
6051	c.646A>G	rs398122704	Benign	Uncertain significance
6052	c.640G>T	rs786203797	Pathogenic	Uncertain significance
6053	c.640G>A	rs786203797	Benign	Uncertain significance
6054	c.639G>T	rs1131692078	Benign	Uncertain significance
6055	c.638G>A	rs1567803140	Pathogenic	Uncertain significance
6056	c.637A>G	rs80357081	Pathogenic	Uncertain significance
6057	c.631G>A	rs1192717688	Benign	Uncertain significance
6058	c.628C>G		Pathogenic	Uncertain significance
6059	c.626C>G		Benign	Uncertain significance
6060	c.626C>T	rs201596327	Benign	Uncertain significance
6061	c.625C>T	rs730881466	Benign	Uncertain significance
6062	c.619A>G		Benign	Uncertain significance
6063	c.617A>C	rs879255494	Benign	Uncertain significance
6064	c.616C>G	rs397509301	Benign	Uncertain significance
6065	c.616C>T	rs397509301	Benign	Uncertain significance
6066	c.611T>G		Benign	Uncertain significance
6067	c.607G>A	rs398122703	Pathogenic	Uncertain significance
6068	c.605A>C	rs1060502366	Benign	Uncertain significance
6069	c.604C>A		Pathogenic	Uncertain significance
6070	c.601G>A	rs80357109	Pathogenic	Uncertain significance
6071	c.599G>A		Pathogenic	Uncertain significance
6072	c.595G>A	rs1555593622	Pathogenic	Uncertain significance
6073	c.594-1G>T	rs757781708	Benign	Uncertain significance
6074	c.593G>A		Pathogenic	Uncertain significance
6075	c.587A>T	rs1555594039	Benign	Uncertain significance
6076	c.586T>C		Benign	Uncertain significance
6077	c.581C>T		Pathogenic	Uncertain significance
6078	c.577A>G	rs878854962	Pathogenic	Uncertain significance
6079	c.566A>G	rs1555594067	Benign	Uncertain significance
6080	c.564A>C		Benign	Uncertain significance
6081	c.562G>A	rs1555594069	Pathogenic	Uncertain significance
6082	c.557C>T	rs55688530	Benign	Uncertain significance
6083	c.556T>G	rs397509298	Pathogenic	Uncertain significance
6084	c.551C>T	rs878854961	Benign	Uncertain significance
6085	c.550T>A	rs1064795269	Benign	Uncertain significance
6086	c.548G>T	rs1555594081	Pathogenic	Uncertain significance
6087	c.548-4T>C		Benign	Uncertain significance
6088	c.548-6T>C		Benign	Uncertain significance
6089	c.547G>C	rs1135401931	Benign	Uncertain significance
6090	c.546G>T	rs1464752950	Benign	Uncertain significance
6091	c.544T>A	rs1060502339	Benign	Uncertain significance
6092	c.539T>C	rs1567805965	Benign	Uncertain significance
6093	c.538A>G		Benign	Uncertain significance
6094	c.538A>C	rs1555594803	Benign	Uncertain significance
6095	c.535T>C	rs587781761	Benign	Uncertain significance
6096	c.533T>A	rs876660085	Benign	Uncertain significance
6097	c.532G>C	rs1259369962	Benign	Uncertain significance
6098	c.530C>T		Benign	Uncertain significance
6099	c.530C>G	rs753940026	Benign	Uncertain significance
6100	c.529T>A	rs1555594827	Benign	Uncertain significance
6101	c.527C>T	rs587782747	Benign	Uncertain significance

6102	c.527C>G	rs587782747	Benign	Uncertain significance
6103	c.523A>G	rs1567806027	Benign	Uncertain significance
6104	c.521A>G	rs1567806035	Pathogenic	Uncertain significance
6105	c.521A>C	rs1567806035	Benign	Uncertain significance
6106	c.515A>G	rs1555594863	Benign	Uncertain significance
6107	c.506A>C	rs587780803	Benign	Uncertain significance
6108	c.503A>C	rs273901743	Benign	Uncertain significance
6109	c.502A>G	rs886040263	Benign	Uncertain significance
6110	c.499A>G		Benign	Uncertain significance
6111	c.493C>G		Benign	Uncertain significance
6112	c.491C>T	rs1555594889	Benign	Uncertain significance
6113	c.490A>C	rs80357384	Benign	Uncertain significance
6114	c.488G>C	rs1369043501	Benign	Uncertain significance
6115	c.485T>G		Benign	Uncertain significance
6116	c.484G>A	rs55816927	Pathogenic	Uncertain significance
6117	c.483T>G	rs1060504575	Benign	Uncertain significance
6118	c.478G>A	rs62625285	Pathogenic	Uncertain significance
6119	c.470C>G	rs80357045	Benign	Uncertain significance
6120	c.469T>C	rs80356897	Benign	Uncertain significance
6121	c.466C>T	rs587778115	Benign	Uncertain significance
6122	c.466C>A	rs587778115	Benign	Uncertain significance
6123	c.465A>T	rs864622260	Benign	Uncertain significance
6124	c.465A>C	rs864622260	Benign	Uncertain significance
6125	c.460G>A	rs1064793318	Pathogenic	Uncertain significance
6126	c.458G>A		Benign	Uncertain significance
6127	c.457A>G	rs28897674	Pathogenic	Uncertain significance
6128	c.455T>C	rs80357275	Benign	Uncertain significance
6129	c.445G>A	rs876658381	Benign	Uncertain significance
6130	c.442-1G>A		Pathogenic	Uncertain significance
6131	c.441+4A>G		Benign	Uncertain significance
6132	c.441G>T	rs748876625	Benign	Uncertain significance
6133	c.439T>G	rs794727800	Benign	Uncertain significance
6134	c.437C>T	rs1060502358	Benign	Uncertain significance
6135	c.436T>C	rs1555596290	Benign	Uncertain significance
6136	c.434C>T	rs886041143	Benign	Uncertain significance
6137	c.433C>A		Benign	Uncertain significance
6138	c.429A>C	rs397507228	Benign	Uncertain significance
6139	c.427G>C	rs80356991	Benign	Uncertain significance
6140	c.424C>G	rs397509156	Benign	Uncertain significance
6141	c.420T>G	rs730881448	Benign	Uncertain significance
6142	c.419G>A	rs1064793055	Benign	Uncertain significance
6143	c.416A>G	rs786202213	Pathogenic	Uncertain significance
6144	c.413T>C	rs200449040	Benign	Uncertain significance
6145	c.412C>G	rs587782724	Benign	Uncertain significance
6146	c.410T>A	rs751078452	Benign	Uncertain significance
6147	c.410T>C	rs751078452	Benign	Uncertain significance
6148	c.401C>A		Benign	Uncertain significance
6149	c.400G>A		Pathogenic	Uncertain significance
6150	c.398G>T		Benign	Uncertain significance
6151	c.395A>G		Benign	Uncertain significance
6152	c.391A>G	rs80357207	Pathogenic	Uncertain significance
6153	c.389A>G	rs56055578	Pathogenic	Uncertain significance
6154	c.386G>T	rs764231119	Benign	Uncertain significance
6155	c.385G>T	rs1555596362	Benign	Uncertain significance

6156	c.385G>A	rs1555596362	Pathogenic	Uncertain significance
6157	c.383T>C	rs1064793053	Benign	Uncertain significance
6158	c.382A>G	rs864622124	Pathogenic	Uncertain significance
6159	c.381T>G	rs945168715	Benign	Uncertain significance
6160	c.380G>T	rs80357189	Benign	Uncertain significance
6161	c.380G>A	rs80357189	Benign	Uncertain significance
6162	c.373A>G	rs587776489	Pathogenic	Uncertain significance
6163	c.372C>G	rs273900715	Pathogenic	Uncertain significance
6164	c.370A>C	rs80357448	Benign	Uncertain significance
6165	c.364G>C	rs1555596409	Benign	Uncertain significance
6166	c.362A>G	rs1555596419	Benign	Uncertain significance
6167	c.359A>G	rs587781491	Benign	Uncertain significance
6168	c.358G>A	rs587782882	Pathogenic	Uncertain significance
6169	c.352C>G	rs876659315	Benign	Uncertain significance
6170	c.350A>T		Benign	Uncertain significance
6171	c.349C>T	rs1042173379	Benign	Uncertain significance
6172	c.347A>G		Benign	Uncertain significance
6173	c.344C>T	rs876659528	Benign	Uncertain significance
6174	c.343C>T	rs1468589409	Benign	Uncertain significance
6175	c.343C>A	rs1468589409	Benign	Uncertain significance
6176	c.341C>G	rs786202620	Benign	Uncertain significance
6177	c.339C>G	rs587779367	Benign	Uncertain significance
6178	c.338A>G	rs587780800	Benign	Uncertain significance
6179	c.334A>G	rs587782017	Benign	Uncertain significance
6180	c.333A>C	rs1567810451	Benign	Uncertain significance
6181	c.332A>T	rs80357312	Pathogenic	Uncertain significance
6182	c.332A>C	rs80357312	Benign	Uncertain significance
6183	c.331G>A		Benign	Uncertain significance
6184	c.328A>G	rs878854946	Benign	Uncertain significance
6185	c.325A>G	rs1555596474	Benign	Uncertain significance
6186	c.319T>C		Benign	Uncertain significance
6187	c.319T>A	rs878854944	Pathogenic	Uncertain significance
6188	c.316A>C	rs786202937	Benign	Uncertain significance
6189	c.310A>C		Benign	Uncertain significance
6190	c.304G>A	rs1555596491	Pathogenic	Uncertain significance
6191	c.302A>G	rs587781798	Benign	Uncertain significance
6192	c.301T>C	rs1555596637	Benign	Uncertain significance
6193	c.296T>C	rs1567811021	Benign	Uncertain significance
6194	c.292G>C	rs80357409	Benign	Uncertain significance
6195	c.290C>T	rs431825393	Benign	Uncertain significance
6196	c.290C>G	rs431825393	Benign	Uncertain significance
6197	c.289A>G	rs1404795980	Benign	Uncertain significance
6198	c.286G>C	rs80357110	Benign	Uncertain significance
6199	c.286G>A	rs80357110	Pathogenic	Uncertain significance
6200	c.275C>G		Benign	Uncertain significance
6201	c.274G>C	rs863224755	Benign	Uncertain significance
6202	c.272G>C		Benign	Uncertain significance
6203	c.272G>A	rs1333635543	Pathogenic	Uncertain significance
6204	c.271T>C	rs786203939	Benign	Uncertain significance
6205	c.269T>C	rs80357174	Benign	Uncertain significance
6206	c.268A>G	rs1555596673	Pathogenic	Uncertain significance
6207	c.266T>C	rs80357097	Benign	Uncertain significance
6208	c.260T>G		Benign	Uncertain significance
6209	c.259T>G	rs80357091	Benign	Uncertain significance

6210	c.255G>T	rs756499058	Benign	Uncertain significance
6211	c.247G>A	rs1060502343	Pathogenic	Uncertain significance
6212	c.247G>T	rs1060502343	Benign	Uncertain significance
6213	c.242A>T	rs886040862	Pathogenic	Uncertain significance
6214	c.241C>G	rs80357350	Pathogenic	Uncertain significance
6215	c.235T>G		Benign	Uncertain significance
6216	c.230C>T	rs80357209	Benign	Uncertain significance
6217	c.230C>G	rs80357209	Benign	Uncertain significance
6218	c.228T>G		Benign	Uncertain significance
6219	c.224A>T		Pathogenic	Uncertain significance
6220	c.222A>C	rs730881465	Benign	Uncertain significance
6221	c.220C>A	rs80357234	Benign	Uncertain significance
6222	c.216C>G	rs80356967	Benign	Uncertain significance
6223	c.216C>A	rs80356967	Benign	Uncertain significance
6224	c.213-14C>G	rs1060502337	Benign	Uncertain significance
6225	c.212+11T>A		Benign	Uncertain significance
6226	c.207C>T		Benign	Uncertain significance
6227	c.199G>A	rs80357102	Pathogenic	Uncertain significance
6228	c.198T>A		Benign	Uncertain significance
6229	c.189A>T	rs80356956	Benign	Uncertain significance
6230	c.185C>G	rs786202286	Benign	Uncertain significance
6231	c.179A>G	rs373655067	Pathogenic	Uncertain significance
6232	c.176C>T		Benign	Uncertain significance
6233	c.172C>T	rs397508904	Benign	Uncertain significance
6234	c.172C>A	rs397508904	Benign	Uncertain significance
6235	c.172C>G	rs397508904	Benign	Uncertain significance
6236	c.170G>C		Benign	Uncertain significance
6237	c.169G>A	rs879255289	Pathogenic	Uncertain significance
6238	c.169G>C	rs879255289	Benign	Uncertain significance
6239	c.165G>A		Pathogenic	Uncertain significance
6240	c.161A>G	rs397507189	Pathogenic	Uncertain significance
6241	c.160C>A	rs80356864	Pathogenic	Uncertain significance
6242	c.155T>C	rs1060502346	Benign	Uncertain significance
6243	c.152T>C	rs1555597285	Benign	Uncertain significance
6244	c.150A>T	rs1555597289	Benign	Uncertain significance
6245	c.146T>G	rs273897660	Benign	Uncertain significance
6246	c.142A>G	rs879255477	Benign	Uncertain significance
6247	c.139T>C		Benign	Uncertain significance
6248	c.135A>T	rs80356883	Pathogenic	Uncertain significance
6249	c.135-4A>G		Pathogenic	Uncertain significance
6250	c.134A>C	rs80356863	Benign	Uncertain significance
6251	c.128T>C	rs1298544053	Benign	Uncertain significance
6252	c.127T>C	rs1555599214	Benign	Uncertain significance
6253	c.124A>C	rs80357163	Benign	Uncertain significance
6254	c.118G>A	rs879255290	Pathogenic	Uncertain significance
6255	c.114G>T	rs1800062	Benign	Uncertain significance
6256	c.107C>A	rs183557525	Benign	Uncertain significance
6257	c.106T>A	rs905812561	Benign	Uncertain significance
6258	c.103G>T	rs879255284	Benign	Uncertain significance
6259	c.101C>T	rs786203319	Benign	Uncertain significance
6260	c.100C>T	rs1064793357	Benign	Uncertain significance
6261	c.98A>C	rs876660844	Benign	Uncertain significance
6262	c.97G>C	rs80357066	Benign	Uncertain significance
6263	c.96G>C		Benign	Uncertain significance

6264	c.91A>G		Benign	Uncertain significance
6265	c.86A>G	rs773841328	Benign	Uncertain significance
6266	c.83T>C	rs80357266	Benign	Uncertain significance
6267	c.82C>G		Benign	Uncertain significance
6268	c.80G>C	rs1064793052	Benign	Uncertain significance
6269	c.77T>C		Benign	Uncertain significance
6270	c.76A>T		Benign	Uncertain significance
6271	c.74C>T	rs876660096	Benign	Uncertain significance
6272	c.71G>A	rs80357198	Pathogenic	Uncertain significance
6273	c.70T>G		Benign	Uncertain significance
6274	c.69G>C	rs766004110	Benign	Uncertain significance
6275	c.68A>C		Benign	Uncertain significance
6276	c.67G>C	rs372047427	Benign	Uncertain significance
6277	c.66A>C	rs786202533	Pathogenic	Uncertain significance
6278	c.62T>G	rs1135401834	Benign	Uncertain significance
6279	c.60A>C	rs202168814	Benign	Uncertain significance
6280	c.56A>G		Pathogenic	Uncertain significance
6281	c.54G>A		Pathogenic	Uncertain significance
6282	c.53T>G	rs80356929	Benign	Uncertain significance
6283	c.53T>A	rs80356929	Benign	Uncertain significance
6284	c.49G>T	rs1402064476	Benign	Uncertain significance
6285	c.48T>A	rs1555600963	Benign	Uncertain significance
6286	c.44T>C	rs80357316	Benign	Uncertain significance
6287	c.40G>T		Benign	Uncertain significance
6288	c.37A>G		Benign	Uncertain significance
6289	c.35A>G	rs1555601006	Benign	Uncertain significance
6290	c.34C>G		Benign	Uncertain significance
6291	c.32T>G	rs80357017	Benign	Uncertain significance
6292	c.32T>C	rs80357017	Benign	Uncertain significance
6293	c.31G>C	rs1555601019	Benign	Uncertain significance
6294	c.25G>C	rs1567823437	Benign	Uncertain significance
6295	c.20G>T		Benign	Uncertain significance
6296	c.19C>A	rs80356994	Benign	Uncertain significance
6297	c.16C>T		Benign	Uncertain significance
6298	c.14C>T	rs1335137805	Benign	Uncertain significance
6299	c.13G>C		Benign	Uncertain significance
6300	c.11C>T	rs786203152	Benign	Uncertain significance
6301	c.9A>T	rs780157871	Benign	Uncertain significance
6302	c.-1A>G	rs587781565	Benign	Uncertain significance
6303	c.-1A>C	rs587781565	Benign	Uncertain significance
6304	c.-2A>T	rs273899693	Pathogenic	Uncertain significance
6305	c.-3G>T	rs273900720	Pathogenic	Uncertain significance
6306	c.-3G>C	rs273900720	Benign	Uncertain significance
6307	c.-9C>G	rs1555601059	Benign	Uncertain significance
6308	c.-11A>C	rs273897654	Benign	Uncertain significance
6309	c.-13G>A	rs431825383	Pathogenic	Uncertain significance
6310	c.-14T>C	rs273897661	Benign	Uncertain significance
6311	c.-20+59G>T	rs1057517547	Benign	Uncertain significance
6312	c.-20+3A>T		Pathogenic	Uncertain significance
6313	c.-20+3A>G	rs1064795882	Pathogenic	Uncertain significance
6314	c.-20+2T>C	rs1567824383	Benign	Uncertain significance
6315	c.-20+1G>C		Benign	Uncertain significance
6316	c.-20G>A	rs886039588	Benign	Uncertain significance
6317	c.-22A>G	rs886052976	Benign	Uncertain significance

6318	c.-42T>G	rs544342552	Benign	Uncertain significance
6319	c.-44C>T	rs1262271602	Benign	Uncertain significance
6320	c.-61C>T	rs549332987	Benign	Uncertain significance
6321	c.-62G>A	rs776540382	Benign	Uncertain significance

(ClinVar)
ions of pathogenicity
ions of pathogenicity
ions of pathogenicity
ions of pathogenicity
ions of pathogenicity
ions of pathogenicity
ions of pathogenicity
ions of pathogenicity
ions of pathogenicity
ions of pathogenicity
ions of pathogenicity
ions of pathogenicity
ions of pathogenicity
ions of pathogenicity
ions of pathogenicity
ions of pathogenicity
ions of pathogenicity
ions of pathogenicity
ions of pathogenicity
ions of pathogenicity
ions of pathogenicity
ions of pathogenicity
ions of pathogenicity
ions of pathogenicity
ions of pathogenicity
ions of pathogenicity
ions of pathogenicity
ions of pathogenicity
ions of pathogenicity
ions of pathogenicity
ions of pathogenicity
ions of pathogenicity
ions of pathogenicity
ions of pathogenicity
ions of pathogenicity
ions of pathogenicity
ions of pathogenicity
ions of pathogenicity
ions of pathogenicity
ions of pathogenicity
ions of pathogenicity
ions of pathogenicity
ions of pathogenicity
ions of pathogenicity
ions of pathogenicity
ions of pathogenicity
ions of pathogenicity
ions of pathogenicity
ions of pathogenicity
ions of pathogenicity
ions of pathogenicity
ions of pathogenicity
ions of pathogenicity

ions of pathogenicity
ions of pathogenicity
ions of pathogenicity
ions of pathogenicity
ions of pathogenicity
ions of pathogenicity
ions of pathogenicity
ions of pathogenicity
ions of pathogenicity
ions of pathogenicity
ions of pathogenicity
ions of pathogenicity
ions of pathogenicity
ions of pathogenicity
ions of pathogenicity
ions of pathogenicity
ions of pathogenicity
ions of pathogenicity
ions of pathogenicity
ions of pathogenicity
ions of pathogenicity
ions of pathogenicity
ions of pathogenicity
ions of pathogenicity
ions of pathogenicity
ions of pathogenicity
ions of pathogenicity
ions of pathogenicity
ions of pathogenicity
ions of pathogenicity
ions of pathogenicity
ions of pathogenicity
ions of pathogenicity
ions of pathogenicity
ions of pathogenicity
ions of pathogenicity
ions of pathogenicity
ions of pathogenicity
ions of pathogenicity
ions of pathogenicity
ions of pathogenicity
ions of pathogenicity
ions of pathogenicity
ions of pathogenicity
ions of pathogenicity
ions of pathogenicity
ions of pathogenicity
ions of pathogenicity
ions of pathogenicity
ions of pathogenicity
ions of pathogenicity
ions of pathogenicity
ions of pathogenicity
ions of pathogenicity

ions of pathogenicity
ions of pathogenicity
ions of pathogenicity
ions of pathogenicity
ions of pathogenicity
ions of pathogenicity
ions of pathogenicity
ions of pathogenicity
ions of pathogenicity
ions of pathogenicity
ions of pathogenicity
ions of pathogenicity
ions of pathogenicity
ions of pathogenicity
ions of pathogenicity
ions of pathogenicity
ions of pathogenicity
ions of pathogenicity
ions of pathogenicity
ions of pathogenicity
ions of pathogenicity
ions of pathogenicity
ions of pathogenicity
ions of pathogenicity
ions of pathogenicity
ions of pathogenicity
ions of pathogenicity
ions of pathogenicity
ions of pathogenicity
ions of pathogenicity
ions of pathogenicity
ions of pathogenicity
ions of pathogenicity
ions of pathogenicity
ions of pathogenicity
ions of pathogenicity
ions of pathogenicity
ions of pathogenicity
ions of pathogenicity
ions of pathogenicity
ions of pathogenicity
ions of pathogenicity
ions of pathogenicity
ions of pathogenicity
ions of pathogenicity
ions of pathogenicity
ions of pathogenicity
ions of pathogenicity
ions of pathogenicity
ions of pathogenicity
ions of pathogenicity
ions of pathogenicity
ions of pathogenicity
ions of pathogenicity

ions of pathogenicity
ions of pathogenicity
ions of pathogenicity
ions of pathogenicity
ions of pathogenicity
ions of pathogenicity
ions of pathogenicity
ions of pathogenicity
ions of pathogenicity
ions of pathogenicity
ions of pathogenicity
ions of pathogenicity
ions of pathogenicity
ions of pathogenicity
ions of pathogenicity
ions of pathogenicity
ions of pathogenicity
ions of pathogenicity
ions of pathogenicity
ions of pathogenicity
ions of pathogenicity
ions of pathogenicity
ions of pathogenicity
ions of pathogenicity
ions of pathogenicity
ions of pathogenicity
ions of pathogenicity
ions of pathogenicity
ions of pathogenicity
ions of pathogenicity
ions of pathogenicity
ions of pathogenicity
ions of pathogenicity
ions of pathogenicity
ions of pathogenicity
ions of pathogenicity
ions of pathogenicity
ions of pathogenicity
ions of pathogenicity
ions of pathogenicity
ions of pathogenicity
ions of pathogenicity
ions of pathogenicity
ions of pathogenicity
ions of pathogenicity
ions of pathogenicity
ions of pathogenicity
ions of pathogenicity
ions of pathogenicity
ions of pathogenicity
ions of pathogenicity
ions of pathogenicity
ions of pathogenicity
ions of pathogenicity

ions of pathogenicity
ions of pathogenicity
ions of pathogenicity
ions of pathogenicity
ions of pathogenicity
ions of pathogenicity
ions of pathogenicity
ions of pathogenicity
ions of pathogenicity
ions of pathogenicity
ions of pathogenicity
ions of pathogenicity
ions of pathogenicity
ions of pathogenicity
ions of pathogenicity
ions of pathogenicity
ions of pathogenicity
ions of pathogenicity
ions of pathogenicity
ions of pathogenicity
ions of pathogenicity
ions of pathogenicity
ions of pathogenicity
ions of pathogenicity
ions of pathogenicity
ions of pathogenicity
ions of pathogenicity
ions of pathogenicity
ions of pathogenicity
ions of pathogenicity
ions of pathogenicity
ions of pathogenicity
ions of pathogenicity
ions of pathogenicity
ions of pathogenicity
ions of pathogenicity
ions of pathogenicity
ions of pathogenicity
ions of pathogenicity
ions of pathogenicity
ions of pathogenicity
ions of pathogenicity
ions of pathogenicity
ions of pathogenicity
ions of pathogenicity
ions of pathogenicity
ions of pathogenicity
ions of pathogenicity
ions of pathogenicity
